# Characteristics of enrolment in an intensive home-visiting programme among eligible first-time adolescent mothers in England: a linked administrative data cohort study

**DOI:** 10.1136/jech-2021-217986

**Published:** 2022-10-05

**Authors:** Francesca L Cavallaro, Ruth Gilbert, Linda PMM Wijlaars, Eilis Kennedy, Emma Howarth, Sally Kendall, Jan van der Meulen, Maria Andreea Calin, Lynne Reed, Katie Harron

**Affiliations:** 1 Population Policy and Practice, UCL Great Ormond Street Institute of Child Health, London, UK; 2 Children, Young Adults and Families Directorate, Tavistock and Portman NHS Foundation Trust, London, UK; 3 School of Psychology, University of East London, London, UK; 4 Centre for Health Services Studies, University of Kent, Canterbury, UK; 5 Department of Health Services Research & Policy, London School of Hygiene and Tropical Medicine, Faculty of Public Health and Policy, London, UK; 6 Clinical Effectiveness Unit, Royal College of Surgeons of England, London, UK; 7 Family Nurse Partnership and Intensive Parenting National Unit, Office for Health Improvement and Disparities, London, UK

**Keywords:** ADOLESCENT, CHILD HEALTH, PUBLIC HEALTH

## Abstract

**Background:**

Intensive home visiting for adolescent mothers may help reduce health disparities. Given limited resources, such interventions need to be effectively targeted. We evaluated which mothers were enrolled in the Family Nurse Partnership (FNP), an intensive home-visiting service for first-time young mothers commissioned in >130 local authorities in England since 2007.

**Methods:**

We created a population-based cohort of first-time mothers aged 13–19 years giving birth in English National Health Service hospitals between 1 April 2010 and 31 March 2017, using administrative hospital data linked with FNP programme, educational and social care data. Mothers living in a local authority with an active FNP site were eligible. We described variation in enrolment rates across sites, and identified maternal and FNP site characteristics associated with enrolment.

**Results:**

Of 110 520 eligible mothers, 25 680 (23.2% (95% CI: 23.0% to 23.5%)) were enrolled. Enrolment rates varied substantially across 122 sites (range: 11%–68%), and areas with greater numbers of first-time adolescent mothers achieved lower enrolment rates. Mothers aged 13–15 years were most likely to be enrolled (52%). However, only 26% of adolescent mothers with markers of vulnerability (including living in the most deprived areas and ever having been looked after as a child) were enrolled.

**Conclusion:**

A substantial proportion of first-time adolescent mothers with vulnerability markers were not enrolled in FNP. Variation in enrolment across sites indicates insufficient commissioning of places that is not proportional to level of need, with mothers in areas with large numbers of other adolescent mothers least likely to receive support.

**WHAT IS ALREADY KNOWN ON THIS TOPIC**

- Intensive home-visiting services (such as the Family Nurse Partnership (FNP)) have the potential to reduce adverse child outcomes associated with adolescent motherhood.
- In England, the FNP is only offered to a subset of eligible pregnant adolescents.
- There is a lack of evidence on the characteristics of mothers enrolling (and those who are not enrolled) in targeted interventions.

**WHAT THIS STUDY ADDS**

- Using data from more than 100 000 first-time adolescent mothers in England, we showed that 23% of eligible mothers are enrolled in the FNP.
- Lower enrolment rates were seen in areas with large populations of adolescent mothers; variation in enrolment rates across the country remained after adjusting for maternal risk factors in the eligible population.
- Only half of mothers aged 13–15 years, 44% of those ever looked after by social care services and 40% of those with a history of mental health or adversity-related hospital admissions, were enrolled.

**HOW THIS STUDY MIGHT AFFECT RESEARCH, PRACTICE OR POLICY**

- This research indicates a need for increased commissioning of targeted services relative to level of need in local areas.

## Introduction

Children of adolescent mothers are more likely to experience adverse health outcomes than children of older mothers, partly due to socioeconomic disadvantage.^1–3^ Interventions aiming to reduce these inequalities include the Family Nurse Partnership (FNP), an intensive home-visiting programme supporting first-time mothers, has a strong evidence base from three US randomised trials and is recommended within the UK’s Healthy Child Programme.^4 5^ The FNP aims to improve birth outcomes, child health and development.^6^ Although a randomised trial in England found no evidence of benefit on outcomes including birth weight and hospital admissions before age 2 years, improved development and educational outcomes were reported, and there remains strong support for the programme locally.^5 7–9^

FNP has been commissioned in >130 English local authorities (LAs) since 2007. While eligibility criteria suggest all first-time adolescent mothers are eligible, the service has been rationed to ~25%–30% of adolescent mothers^10^ as sufficient funding for all was not made available. Effective targeting to those with highest need is therefore key for FNP service, with local teams encouraged to decide who to prioritise for enrolment. Evidence suggests that young mothers living in the most deprived areas, with histories of mental health conditions and prior contact with children’s social care are at higher risk of poor infant outcomes.^11–13^ However, little is known about the extent or drivers of variation in the targeting of intensive support to mothers in England.

To address this evidence gap and inform commissioning and targeting of the FNP, we quantified the variation in enrolment rates across 122 FNP sites in England and evaluated maternal vulnerability indicators and site characteristics associated with enrolment. We used population-based administrative data for all eligible mothers in England to generate evidence for decision-makers of targeted preventive services.

## Methods

### Data sources and linkage

The FNP enrolled first-time mothers aged <20 years at their last menstrual period, ≤28 weeks of pregnancy for most of our study period. From November 2016, a few FNP sites enabled enrolment after 28 weeks’ gestation, and among mothers aged 20–24 years with vulnerability markers.^14^

Using data in Hospital Episode Statistics (HES), we constructed a cohort of first-time mothers aged 13–19 years at last menstrual period, living in England, and giving birth in a National Health Service (NHS) hospital between 1 April 2010 and 31 March 2017.^15^ HES is an administrative database with coded information on all admissions to English NHS hospitals.^16^ Each care episode includes ≤20 clinical diagnosis codes from the International Classification of Diseases (ICD-10). Pseudonymised HESIDs link admissions for the same person over time. Information on Accidents & Emergency (A&E) attendance was obtained from the HES A&E dataset.

Records for enrolled FNP participants from the FNP Information System (IS) were linked to HES by a trusted third party (NHS Digital) using a deterministic linkage algorithm including name, NHS number, sex, date of birth and postcode. Of 27 065 FNP participants giving birth in our study period, 27 035 (99.9%) were linked to HES via the NHS Digital algorithm. Another 25 were linked manually based on dates of birth and delivery, birth weight, and LA, or through HES mother–baby linkage developed previously,^15^ leading to >99.9% linkage of FNP mothers to HES.

We used FNP IS data to determine enrolment dates by lower-tier LA, and validated site dates and catchment area with the FNP National Unit.

HES records for all mothers in our cohort were linked to the National Pupil Database (NPD) by a trusted third party (Department for Education), to enrich our dataset with information on maternal education (including Special Educational Needs status and receipt of free school meals) and contact with children’s social care services (including child protection plans and child looked after). A total of 83.5% of adolescent mothers in our cohort were linked to NPD.

### Cohort definition

**Figure 1:**
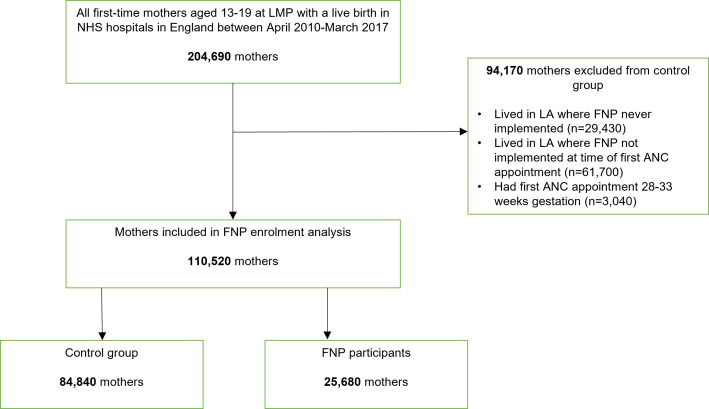
Flow diagram of cohort selection. Ninety mothers who linked to an HESID that contained no inpatient hospital records (eg, due to home delivery), and only A&E attendance records, were excluded from our cohort. Numbers have been rounded to the nearest 5 in accordance with NHS Digital’s statistical disclosure rules for subnational analyses; totals may not be equal to the sum of component categories. ‘First-time mothers’ refers to first live birth; mothers may have had a previous pregnancy ending in miscarriage or abortion (in line with FNP eligibility criteria). A&E, Accidents & Emergency; ANC, antenatal care; FNP, Family Nurse Partnership; HES, Hospital Episode Statistics; LA, local authority; LMP, last menstrual period; NHS, National Health Service.

Our study cohort included all 110 520 eligible mothers aged 13–19 years with a first birth between 1 April 2010 and 31 March 2017, and whose first antenatal booking appointment as recorded in HES (or estimated date of 28 weeks’ gestation, if missing) occurred while there was an active FNP site in their LA of residence (figure 1). Date of last menstrual period was estimated by subtracting gestational age at birth from the date of childbirth, or subtracting 40 weeks (the median gestational age at birth among adolescent mothers) from the date of childbirth, for 13% of mothers with missing data. Mothers whose antenatal booking appointment occurred between 28 and 33 weeks’ gestation were excluded as they would not have met eligibility criteria (figure 1). (We may have excluded a small number of eligible mothers within the few sites that allowed enrolment >28 weeks from November 2016.) We considered recorded gestational age at booking appointment of ≥33 weeks among 6% of mothers to be data errors, and recoded them to 28 weeks to retain these mothers in the cohort. We excluded mothers whose pregnancy ended in stillbirth and those enrolled in FNP for their second delivery (eg, following a previous stillbirth) to ensure comparability with unenrolled mothers. Ten FNP mothers who did not link to HES, and 90 who linked to an HESID with no inpatient hospital records, were also excluded.

### Outcome

Our outcome of interest was enrolment in FNP, as identified by linkage of a mother in FNP IS to a record in HES, regardless of the number of FNP visits received.

### Maternal risk factors

We selected potential risk factors for FNP enrolment based on maternal vulnerability characteristics associated with poor infant outcomes, and available in HES: maternal age, ethnicity and area-level deprivation (Index of Multiple Deprivation quintile).^11^ We also considered maternal unplanned hospital admissions within 2 years prior to 20 weeks’ gestation: mental health-related admissions (excluding self-harm and substance misuse), adversity-related admissions (violence, self-harm or substance misuse) and chronic condition admissions were identified based on published lists of ICD-10 diagnostic codes (online supplemental table 1).^11 17–19^ Having ≥1 A&E attendance within 2 years prior to 20 weeks’ gestation was also considered as a risk factor. We considered educational and social care characteristics before 20 weeks of pregnancy, including ever having a child protection plan or being looked after, having Special Educational Needs, receiving free school meals, living in the Income Deprivation Affecting Children Index (IDACI) bottom decile, school exclusion/pupil referral unit/alternative provision, and persistent absence. We also considered Key Stage 4 educational attainment.

10.1136/jech-2021-217986.supp1 Supplementary data - Supplementary tables and figures

We used 20 weeks’ gestation since 93% of all mothers attend an antenatal booking appointment by this stage.^20^ We defined vulnerability markers as maternal age 13–15 years, living in the most deprived quintile, ever having Special Educational Needs, ever being looked after or having a child protection plan, and previous mental health-related or adversity-related admission.

### Site characteristics

FNP sites consist of a team covering one or more (neighbouring) LAs. We classified sites into enrolment rate quartiles (‘high-enrolment sites’ as those in the highest quartile and ‘low-enrolment sites’ in the lowest quartile), and examined variation in enrolment according to enrolment quartile, geographical region, and year. We hypothesised low-enrolment sites would show more selective targeting of vulnerable mothers, and provide insight into prioritisation of mothers when resources are limited.

### Data analysis

We calculated enrolment rates as the percentage of FNP participants among eligible first-time adolescent mothers living in an LA with an active FNP site at the time of first antenatal appointment, including by site and maternal risk factors. Multilevel logistic regression models with mothers nested within FNP sites were used to calculate crude and adjusted ORs of enrolment. Multivariable models included all maternal risk factors; multicollinearity was assessed using Spearman correlation coefficients. To examine variation in maternal risk factors, we stratified the analysis by site characteristics: high/low-enrolment site, region and financial year of delivery, and tested for interaction with maternal risk factors. We explicitly classified mothers not linking to NPD as ‘unlinked’ in relevant variables to retain them in models.

Lastly, we built crude and adjusted funnel plots of enrolment rates by site according to the size of the eligible adolescent mother population, separately for ages 13–17 and 18–20 years at childbirth, to assess whether variation in enrolment rates was likely due to chance. The outer limits on the plots define the range of percentages that are within three SDs of the national average. If the observed variation was due to chance alone, we would expect only 1 in 500 sites to have a percentage that is outside these limits.

### Secondary analysis

We repeated the analysis for first-time mothers aged 20–24 years at last menstrual period in relevant FNP sites (detailed methods in online supplemental material 2).

10.1136/jech-2021-217986.supp2 Supplementary data - Secondary analysis

## Results

### Description of enrolment

**Figure 2:**
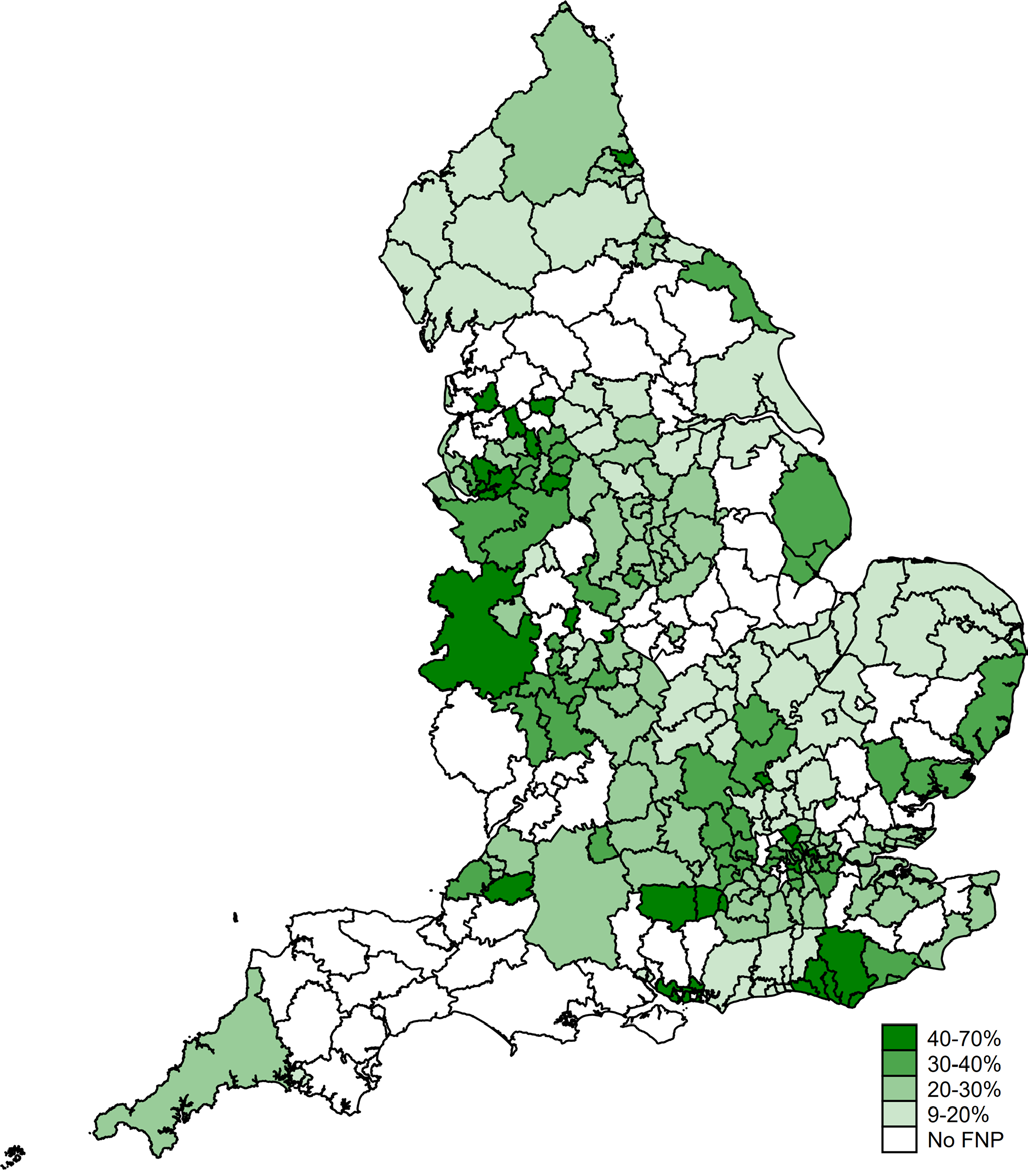
Percentage enrolment in the Family Nurse Partnership (FNP) among eligible adolescent mothers, living in a local authority (LA) with an active FNP site at the time of first antenatal appointment, by Local Authority–England, births between 1 April 2010 and 31 March 2017. The 122 FNP sites active during the study period covered 136 LAs (numbers and geographical boundaries of sites and LAs changed over the study period). Different sites were active for different periods within the 2010–2017 cohort; the FNP was never commissioned in 15 LAs (online supplemental figure 1).

Among all 110 520 eligible adolescent mothers giving birth between April 2010 and March 2017, 25 680 (23.2% (95% CI: 23.0% to 23.5%)) were enrolled in FNP. Enrolment rates ranged across 122 FNP sites from 11% in Cumbria to 68% in Wandsworth (figure 2). Online supplemental table 2 describes the 136 LAs, enrolment dates and rates by FNP site.

Overall, 12.5% (12.4%–12.7%) of all (25 680 of 204 690) first-time adolescent mothers giving birth in England in our study period were enrolled in FNP.

### Risk factors for enrolment

Most eligible first-time adolescent mothers were white (85%), aged 18–19 years at childbirth (55%) and living in the most deprived quintile (49%) (table 1). Five per cent of first-time adolescent mothers had ever been looked after before 20 weeks of pregnancy, 32% had ever been absent ≥10% school half-days within a term and 63% had attempted but not achieved 5 A*-C GCSEs. Characteristics of eligible mothers were similar to LAs having never commissioned FNP (online supplemental table 3). Overall, 52% of eligible adolescent mothers had at least one vulnerability marker.

**Table 1:** Risk factors for FNP enrolment among mothers aged 13–19 years at last menstrual period, living in a local authority with an active FNP site at the time of first antenatal appointment (England, births between April 2010 and March 2017)

	N eligible mothers	N enrolled in FNP	% enrolled in FNP	Crude OR (95% CI)	Adjusted* OR (95% CI)
Total	110 520	25 675	23.2	—	—
Maternal age at birth					
13–15	2380 (2.2)	1240	52.1	4.62 (4.24 to 5.02)	2.65 (2.39 to 2.94)
16–17	22 725 (20.6)	8720	38.4	2.50 (2.42 to 2.59)	1.80 (1.72 to 1.87)
18–19	61 090 (55.3)	12 875	21.1	1 (ref)	1 (ref)
20†	24 325 (22.0)	2840	11.7	0.48 (0.46 to 0.50)	0.56 (0.53 to 0.59)
Ethnicity					
White	93 730 (84.8)	21 845	23.3	1 (ref)	1 (ref)
South Asian	3170 (2.9)	535	16.9	0.55 (0.49 to 0.61)	0.74 (0.67 to 0.83)
Black	3970 (3.6)	1195	30.1	1.18 (1.09 to 1.28)	1.31 (1.21 to 1.43)
Mixed/other	5695 (5.2)	1335	23.4	0.89 (0.83 to 0.95)	0.97 (0.90 to 1.04)
Unknown	3950 (3.6)	770	19.5	0.69 (0.63 to 0.75)	0.84 (0.77 to 0.92)
Index of Multiple Deprivation (quintile)					
Least deprived	5550 (5.0)	1135	20.5	0.80 (0.74 to 0.87)	0.85 (0.78 to 0.92)
2	8565 (7.7)	1820	21.2	0.91 (0.86 to 0.98)	0.95 (0.88 to 1.01)
3	14 835 (13.4)	3330	22.4	1 (ref)	1 (ref)
4	27 520 (24.9)	6430	23.4	1.07 (1.02 to 1.13)	1.02 (0.97 to 1.08)
Most deprived	53 905 (48.8)	12 820	23.8	1.19 (1.14 to 1.25)	1.07 (1.01 to 1.12)
Unknown	145 (0.1)	145	100	—	—
Admission with diagnoses within 2 years before 20 weeks of pregnancy					
Mental health (excluding substance misuse and self-harm)	2420 (2.2)	955	39.5	2.20 (2.03 to 2.40)	1.41 (1.27 to 1.57)
Adversity related (self-harm, substance misuse, violence)	4460 (4.0)	1770	39.7	2.34 (2.20 to 2.50)	1.24 (1.13 to 1.36)
Any chronic condition	9580 (8.7)	3170	33.1	1.74 (1.66 to 1.83)	1.16 (1.09 to 1.25)
A&E visit	68 965 (62.4)	17 815	25.8	1.48 (1.43 to 1.53)	1.29 (1.25 to 1.34)
Gestational age at antenatal booking appointment					
Before 10 weeks	29 390 (26.6)	6810	23.2	1 (ref)	1 (ref)
10–20 weeks	40 640 (36.8)	9540	23.5	0.93 (0.90 to 0.97)	0.90 (0.87 to 0.94)
20 weeks or more	6095 (5.5)	1515	24.9	0.93 (0.87 to 0.99)	0.77 (0.71 to 0.82)
Unknown	34 390 (31.1)	7815	22.7	0.92 (0.89 to 0.96)	0.81 (0.78 to 0.85)
Linked to NPD					
Linked to NPD	92 260 (83.5)	22 980	24.9	1 (ref)	1 (ref)
Not linked to NPD	17 405 (15.7)	2570	14.8	0.46 (0.44 to 0.48)	0.86 (0.81 to 0.92)
Linked to NPD but not to NPD census	855 (0.8)	125	14.6	0.47 (0.38 to 0.56)	0.79 (0.64 to 0.96)
Ever had a child protection plan (CPP) or was looked after before 20 weeks of pregnancy					
No CPP or looked after	85 890 (77.7)	19 860	23.1	1 (ref)	1 (ref)
Looked after (CPP)	5540 (5.0)	2445	44.1	2.60 (2.46 to 2.76)	1.92 (1.81 to 2.04)
CPP, but not looked after	1685 (1.5)	800	47.5	2.95 (2.67 to 3.26)	1.62 (1.46 to 1.80)
Not linked to NPD	17 405 (15.7)	2570	14.8	0.51 (0.49 to 0.53)	—‡
Ever recorded as having Special Educational Needs before 20 weeks of pregnancy					
No	45 270 (41.0)	9190	20.3	1 (ref)	1 (ref)
Yes	46 990 (42.5)	13 790	29.3	1.61 (1.56 to 1.66)	1.22 (1.18 to 1.27)
Not linked to NPD	17 405 (15.7)	2570	14.8	0.60 (0.57 to 0.63)	—‡
Linked to NPD but not to NPD census	855 (0.8)	125	14.6	0.61 (0.50 to 0.74)	—‡
Ever recorded as receiving free school meals before 20 weeks of pregnancy					
No	41 455 (37.5)	8050	19.4	1 (ref)	1 (ref)
Yes	50 805 (46.0)	14 930	29.4	1.69 (1.63 to 1.74)	1.20 (1.16 to 1.24)
Not linked to NPD	17 405 (15.7)	2570	14.8	0.63 (0.60 to 0.66)	—‡
Linked to NPD but not to NPD census	855 (0.8)	125	14.6	0.64 (0.53 to 0.77)	—‡
Ever in IDACI bottom decile before 20 weeks of pregnancy					
No	59 765 (54.1)	13 760	23	1 (ref)	1 (ref)
Yes	32 495 (29.4)	9220	28.4	1.33 (1.29 to 1.38)	1.05 (1.01 to 1.09)
Not linked to NPD	17 405 (15.7)	2570	14.8	0.52 (0.49 to 0.54)	—‡
Linked to NPD but not to NPD census	855 (0.8)	125	14.6	0.53 (0.43 to 0.64)	—‡
Educational attainment before 20 weeks of pregnancy					
Attempted but did not achieve 5 A*-C GCSEs	69 345 (62.7)	16 365	23.6	1 (ref)	1 (ref)
5 A*-C GCSEs	16 960 (15.3)	3320	19.6	0.77 (0.73 to 0.80)	1.05 (1.00 to 1.10)
Not linked to NPD	17 405 (15.7)	2570	14.8	0.49 (0.47 to 0.52)	—‡
Had not attempted GCSEs prior to 20 weeks of pregnancy	6810 (6.2)	3420	50.2	3.56 (3.38 to 3.75)	1.54 (1.44 to 1.64)
Ever excluded, in pupil referral unit, or alternative provision					
No	65 620 (59.0)	14 640	22.3	1 (ref)	1 (ref)
Yes	28 105 (25.2)	8620	30.7	1.55 (1.51 to 1.61)	1.05 (1.01 to 1.08)
Not linked to NPD	17 515 (15.8)	2590	14.8	0.53 (0.51 to 0.56)	—‡
Ever persistently absent in a term (≥10% possible sessions)					
No	58 100 (52.6)	10 533	18.1	1 (ref)	1 (ref)
Yes	35 535 (32.2)	12 725	35.8	2.71 (2.63 to 2.80)	1.44 (1.39 to 1.50)
Not linked to NPD	17 515 (15.8)	2590	14.8	0.71 (0.67 to 0.74)	—‡

Numbers have been rounded to the nearest 5 in accordance with NHS Digital’s statistical disclosure rules for subnational analyses.

*Adjusted models included all variables in the table as covariates.

†Includes only mothers aged 19 years at last menstrual period.

‡Estimates omitted due to multicollinearity.

A&E, Accidents & Emergency; CPP, Child Protection Plan; FNP, Family Nurse Partnership; GCSE, General Certificate of Secondary Education; IDACI, Income Deprivation Affecting Children Index; NHS, National Health Service; NPD, National Pupil Database.

The enrolment rate was highest (52%) among those aged 13–15 years than 20 years at childbirth (12%), although those aged 13–15 years old accounted for only 2% of eligible mothers (table 1). The enrolment rate increased slightly from 21% in the least deprived quintile to 24% in the most deprived. A total of 40% of adolescent mothers with previous mental health or adversity-related admissions were enrolled, as well as 44% of mothers ever looked after and 29% of those ever identified as having Special Educational Needs. Overall, 26% of adolescent mothers with any vulnerability marker were enrolled.

Results from the adjusted model (table 1) showed younger mothers were prioritised for enrolment (the OR decreased from 2.65 (95% CI: 2.39 to 2.94) in mothers aged 13–15 years old to 0.56 (0.53 to 0.59) in mothers aged 20 years, compared with those aged 18–19 years old). Other important risk factors for enrolment included ever having been a child looked after (OR=1.92 (1.81 to 2.04)), ever had a child protection plan (OR=1.62 (1.46 to 1.80)) or ever having been identified as having Special Educational Needs (OR=1.22 (1.18 to 1.27)).

### Variation in maternal risk factors according to site characteristics

Low-enrolment FNP sites included 51% of all eligible mothers in their catchment areas but enrolled ≤21% of these mothers, while high-enrolment sites included only 9% of all eligible mothers and had rates >36% (online supplemental table 4).

The effect of age and ethnicity on enrolment was more pronounced in low-enrolment sites (online supplemental table 5) and varied across regions (online supplemental figure 2, online supplemental table 6). The age gradient appeared in all regions, but was particularly pronounced in the South-West, East Midlands and South-East. In five of nine regions, mothers living in the most deprived areas were more likely to be enrolled than those in the middle quintile of deprivation. Only in London was enrolment higher in the least deprived areas (OR=1.68 (1.08 to 2.63), compared with the middle quintile).

Risk factors for enrolment also varied over time (online supplemental table 7), partly due to changes in regional distribution of active sites.

### Funnel plots of variation in enrolment rates

**Figure 3:**
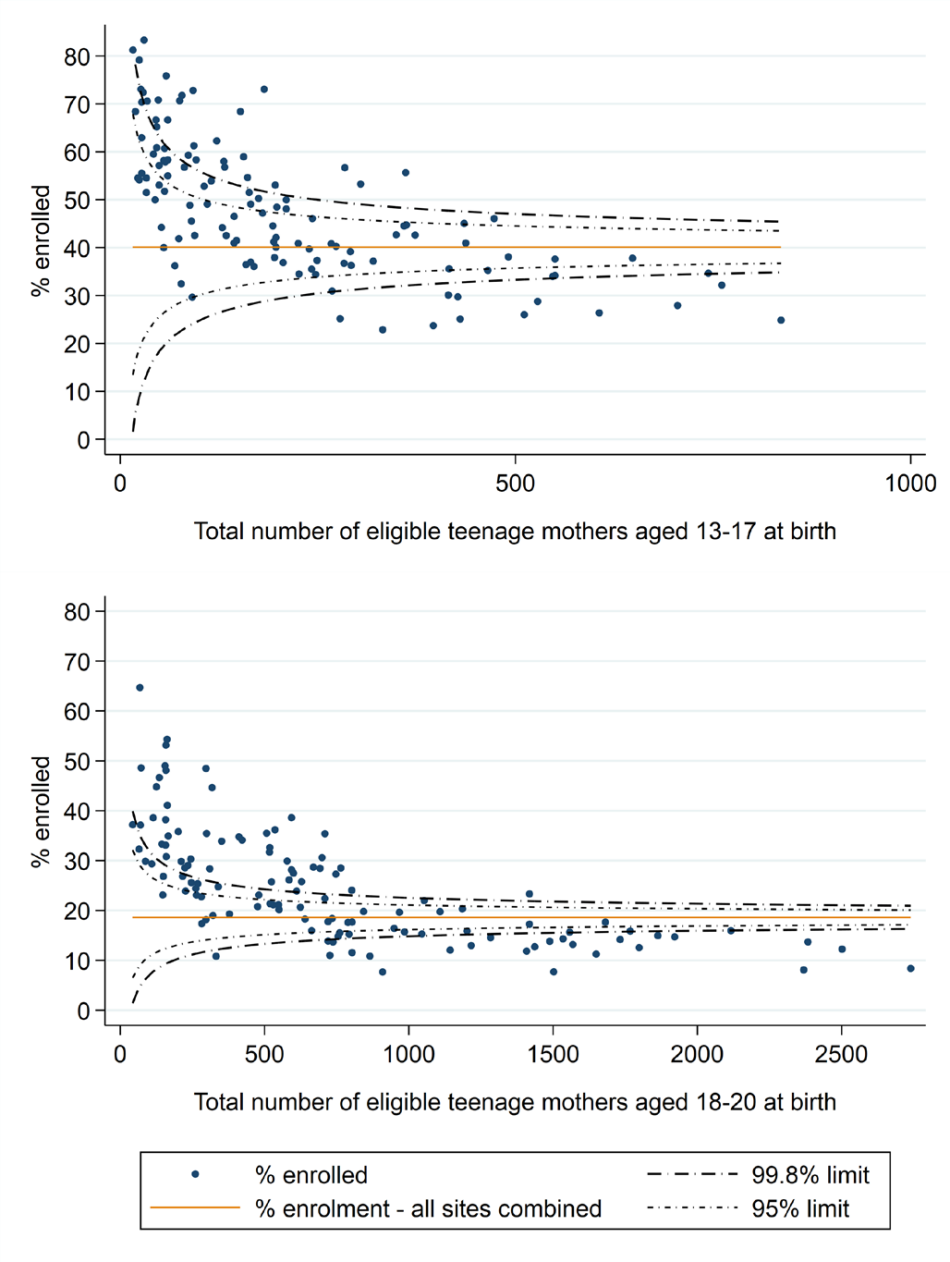
Unadjusted funnel plots of variation in FNP enrolment rates among eligible first-time adolescent mothers across FNP sites, by maternal age–England, births between April 2010 and March 2017. FNP, Family Nurse Partnership.

A substantial proportion of FNP sites’ enrolment rates fell outside the funnel plot limits, indicating that much of the variation in enrolment rates across sites for younger mothers (aged 13–17 years at childbirth) was unexplained by chance (figure 3). There was even more unexplained variation among mothers aged 18–20 years at childbirth, as indicated by the majority of FNP sites falling outside the funnel plot limits. Among both age groups, adjusted enrolment rates were lower than expected in sites with larger numbers of eligible adolescent mothers (online supplemental figure 3).

### Secondary analysis: enrolment among mothers aged 20–24 years

In FNP sites enrolling mothers aged 20–24 years at last menstrual period, the enrolment rate was 3.7% (165 of 4470; 3.1%–4.2%), varying between 2% and 11% across sites. Mothers who were ever looked after, recorded as receiving free school meals or having Special Educational Needs were more likely to be enrolled (online supplemental material 2).

## Discussion

Our study fills an important evidence gap on coverage and targeting of intensive home-visiting services such as FNP within England. We show the most vulnerable mothers are being targeted for FNP, especially the youngest teenagers, and those with prior contact with children’s social care. However, despite all first-time adolescent mothers living in FNP catchment areas being eligible for the programme, only 23% were enrolled between 2010/2011 and 2016/2017. Our findings indicate insufficient commissioning of FNP relative to need: areas with the greatest numbers of adolescent mothers had lower enrolment rates, and variation in enrolment rates remained after adjusting for known differences in maternal risk factors. Overall, only 26% of eligible adolescent mothers with vulnerability markers associated with adverse outcomes were enrolled, including 52% of those aged 13–15 years, 44% of those ever looked after, and 40% of those with prior mental health or adversity-related hospital admissions. These groups represent mothers and infants at significantly greater risk of low birth weight, unplanned hospital admissions for injury and infant mortality.^11^ Moreover, adolescent mothers had similar risks of young maternal age and having been looked after in areas where FNP was never commissioned, and where similar intensive support is not available.

A major strength of our study was the use of a population-based cohort of all first-time adolescent mothers giving birth in English NHS hospitals, linked to routine education and social care records, and >99.9% ascertainment of FNP mothers in HES. We used extensive data quality checks for LA-specific site activity dates and geographical coverage with the FNP National Unit to minimise misclassification of unenrolled eligible mothers. However, complex site histories (including different local areas within sites being commissioned at different times) mean we may have overestimated unenrolled eligible mothers and therefore underestimated some enrolment rates. Conversely, birth ascertainment in HES (97% of total births in English hospitals according to the Office for National Statistics)^15^ means some mothers may have been wrongly excluded from the denominator. Neither underascertainment nor overascertainment of unenrolled eligible mothers is likely to have biased the risk factor analysis, since they should not be associated with risk factors.

Some data limitations are worth noting. First, missing data on gestational age at booking appointment (32%) and birth (13%) required us to make assumptions to determine eligibility. Some mothers with missing gestational age at first antenatal appointment may have booked after 28 weeks but were retained in our cohort (2.6% were excluded due to known booking appointment after 28 weeks, vs 7% expected based on primary care records—S Syed, personal communication). Second, the NPD does not identify mothers who had contact with social services only before starting school, likely underestimating history of social care and effect estimates. The administrative data we used did not capture information on all relevant maternal characteristics or service contacts (eg, community mental health service use). Linkage to primary care and the Maternity Services Dataset could enable investigation of a wider range of risk factors. Given limited resources, individual-level or household-level deprivation measures (including those available through Unique Property Reference Numbers) could allow for more effective targeting and follow-up of the many young mothers experiencing socioeconomic disadvantage.^21^

To our knowledge, this is the first study examining enrolment in a targeted intensive home-visiting programme for expectant mothers. Similar to our findings, two previous studies have shown high variation in acceptance rates for universal home visiting between sites, and higher acceptance rates among higher-risk mothers.^22 23^ In a similar targeted home-visiting intervention for vulnerable families in Canada, unenrolled mothers were more vulnerable than enrolled mothers—contrary to our findings—nonetheless illustrating similar difficulties in enrolling the most vulnerable groups.^24^

Budget cuts since the inception of the FNP in England in 2007 mean that the programme, costing approximately £3000 per client per year, has been offered to a more select group of mothers over time.^25 26^ Our study demonstrates FNP places are not commissioned proportionately to the level of need within local areas, with particularly insufficient commissioning in LAs with higher numbers of adolescent births, contrary to stated aims of prioritising ‘areas with […]the highest numbers of eligible population’.^27^ Pregnant adolescents living in areas with many adolescent mothers are less likely to receive support than those in areas with few adolescent mothers, with important implications for equity. Commissioning an intensive service for only some eligible mothers has potential knock-on effects on those unenrolled, that is, if most adolescent mothers are assumed to be receiving additional support.

Young age is the main eligibility criterion for FNP in England, based on ease of identification, associations with social adversity, disrupted education and employment,^2 28^ and other factors contributing to poor health outcomes among their children.^29–31^ However, only half of mothers aged 13–15 years were enrolled in FNP. Furthermore, other countries additionally focus on low educational level or unemployment,^32–34^ based on evidence of higher effectiveness in socioeconomically deprived groups in the USA.^35–37^ Given strict caseload limits (maximum 25 mothers per family nurse), many sites in England have explicit policies of prioritising younger adolescent mothers. Overall, 74% of mothers with vulnerability markers were not enrolled in FNP, reflecting a failure of policy and commissioning to address vulnerable mothers’ needs, with important variation in who is offered intensive services across England.

There are several explanations and implications of this. First, identifying vulnerable mothers may be challenging, due to limited and variable information on vulnerabilities available to referring health providers and FNP teams. Although FNP teams may prioritise mothers with additional vulnerabilities not captured in administrative data we used (homelessness or community mental health service use), we show that only 44% of mothers looked after as children were enrolled. Some vulnerabilities (such as family violence) may be disclosed only after a trusting relationship is built with their family nurse or health visitor,^38–40^ underscoring the need for effective universal services. Second, there is uncertainty regarding which vulnerable mothers are likely to accept and benefit from intensive support. Ongoing evaluations of the FNP will determine which subgroups of young mothers benefit the most from FNP and inform decisions by referring clinicians.^41^ Given insufficient funding for universal offer, family nurses and referring providers need standardised, real-time information on vulnerabilities for all mothers to support decision-making and better target the FNP. Work on defining vulnerabilities by the FNP National Unit will support sites to determine priority criteria for their local context.

Third, vulnerable mothers may have higher refusal rates. FNP’s fidelity target is to enrol 75% of mothers offered FNP; aggregate site data suggest not all sites meet this target. We were unable to determine whether under-representation of some groups, for example, South Asian adolescent mothers, was because they were less likely to be offered a place, accept it or both. FNP teams report most mothers who decline feel socially well supported, although some decliners are especially vulnerable (eg, involved with social care services).^42^ Last, more vulnerable mothers may be unknown to midwifery services, due to enrolling after 28 weeks (eg, due to moving between LAs in pregnancy). Individual-level information on who is approached and who accepts would help inform strategies to increase uptake among especially vulnerable mothers.

To successfully reduce social inequalities, interventions need to be commissioned for all families with higher need. In 2010–2017, most adolescent mothers at highest risk of adverse outcomes were not receiving intensive support during and after pregnancy. Due to variation in service delivery across England, eligible adolescent mothers living in areas with many adolescent mothers were least likely to receive FNP support. Moreover, adolescent mothers with similar vulnerabilities had no access to equivalent support in areas where FNP was not commissioned. Commissioning should aim to provide adequate support to meet the needs of all adolescent mothers (not just a subset of them), through increased provision of intensive services in line with local need, including for mothers not eligible for FNP. With limited resources and pressure on health visiting services, decisions about the appropriate level of care for each family should be based on continuous evaluations of who is most likely to benefit, supported by more complete recording of vulnerabilities antenatally and real-time linkage of routine health and social care data.^11 43^

## Data Availability

Data may be obtained from a third party and are not publicly available. We are unable to share the individual data used for this study. HES and FNP data can be requested through NHS Digital, NPD data can be requested through the Department for Education.

## References

[1] Lawlor D , Shaw M , Johns S . Teenage pregnancy is not a public health problem. BMJ 2001;323:1428. 10.1136/bmj.323.7326.1428 PMC1121872 11744574

[2] Wiggins M , Oakley A , Sawtell M . Teenage parenthood and social exclusion: a multi-method study - Summary report of findings, 2005. Available: https://discovery.ucl.ac.uk/id/eprint/10003007/1/Wiggins2005TeenageParenthood.pdf [Accessed Dec 2020].

[3] Basu S , Gorry D . Consequences of teenage childbearing on child health. Econ Hum Biol 2021;42:101019. 10.1016/j.ehb.2021.101019 34091239

[4] Marmot M . Fair Society, Hearly lives: the Marmot review, 2010. Available: http://www.instituteofhealthequity.org/resources-reports/fair-society-healthy-lives-the-marmot-review/fair-society-healthy-lives-full-report-pdf.pdf [Accessed Jan 2021].

[5] Robling M , Bekkers M-J , Bell K , et al . Effectiveness of a nurse-led intensive home-visitation programme for first-time teenage mothers (building blocks): a pragmatic randomised controlled trial. Lancet 2016;387:146–55. 10.1016/S0140-6736(15)00392-X 26474809 PMC4707160

[6] Olds DL , Hill PL , O'Brien R , et al . Taking preventive intervention to scale: the nurse-family partnership. Cogn Behav Pract 2003;10:278–90. 10.1016/S1077-7229(03)80046-9

[7] Robling M , Lugg-Widger F , Cannings-John R , et al . The family nurse partnership to reduce maltreatment and improve child health and development in young children: the BB:2–6 routine data-linkage follow-up to earlier RCT. Public Health Research 2021;9:1–160. 10.3310/phr09020 33570895

[8] Olds D . Building evidence to improve maternal and child health. Lancet 2016;387:105–7. 10.1016/S0140-6736(15)00476-6 26474806

[9] Barlow J , Barnes J , Sylva K , et al . Questioning the outcome of the building blocks trial. The Lancet 2016;387:1615–6. 10.1016/S0140-6736(16)30201-X 27116069

[10] Harron K , Mc Grath-Lone L , Mason S . Using linked administrative data for monitoring and evaluating the family nurse partnership in England: a scoping report, 2016. Available: http://repository.tavistockandportman.ac.uk/1448/ [Accessed Dec 2020].

[11] Harron K , Gilbert R , Fagg J , et al . Associations between pre-pregnancy psychosocial risk factors and infant outcomes: a population-based cohort study in England. Lancet Public Health 2021;6:e97–105. 10.1016/S2468-2667(20)30210-3 33516292 PMC7848754

[12] Brown HK , Cobigo V , Lunsky Y , et al . Maternal and offspring outcomes in women with intellectual and developmental disabilities: a population-based cohort study. BJOG 2017;124:757–65. 10.1111/1471-0528.14120 27222439

[13] Botchway SK , Quigley MA , Gray R . Pregnancy-Associated outcomes in women who spent some of their childhood looked after by local authorities: findings from the UK millennium cohort study. BMJ Open 2014;4:e005468. 10.1136/bmjopen-2014-005468 PMC4267074 25510884

[14] Family Nurse Partnership National Unit & Dartington Service Design Lab . FNP adapt: using evidence, pragmatism and collaboration to change the FNP programme in London, 2020. Available: https://www.fnp.nhs.uk/media/1359/fnp_adapt_report_web.pdf [Accessed Dec 2020].

[15] Harron K , Gilbert R , Cromwell D , et al . Linking data for mothers and babies in De-Identified electronic health data. PLoS One 2016;11:e0164667. 10.1371/journal.pone.0164667 27764135 PMC5072610

[16] Herbert A , Wijlaars L , Zylbersztejn A , et al . Data resource profile: Hospital episode statistics admitted patient care (hES APC). Int J Epidemiol 2017;46:1093–1093i. 10.1093/ije/dyx015 28338941 PMC5837677

[17] Hardelid P , Dattani N , Gilbert R , et al . Estimating the prevalence of chronic conditions in children who die in England, Scotland and Wales: a data linkage cohort study. BMJ Open 2014;4:e005331. 10.1136/bmjopen-2014-005331 PMC4127921 25085264

[18] Herbert A , Gilbert R , González-Izquierdo A , et al . Violence, self-harm and drug or alcohol misuse in adolescents admitted to hospitals in England for injury: a retrospective cohort study. BMJ Open 2015;5:e006079. 10.1136/bmjopen-2014-006079 PMC4322205 25667148

[19] Pearson RJ , Jay MA , Wijlaars LPMM , et al . Association between health indicators of maternal adversity and the rate of infant entry to local authority care in England: a longitudinal ecological study. BMJ Open 2020;10:e036564. 10.1136/bmjopen-2019-036564 PMC7430489 32792438

[20] NHS Digital . Maternity Services Monthly Statistics November 2019, experimental statistics - Gestational age at booking appointment, 2020. Available: https://digital.nhs.uk/data-and-information/publications/statistical/maternity-services-monthly-statistics/november-2019/analysis [Accessed Feb 2021].

[21] Geospatial Commission . Unlocking the power of location. The UK’s Geospatial Strategy, 2020 to 2025, 2020.

[22] Sword WA , Krueger PD , Watt MS . Predictors of acceptance of a postpartum public health nurse home visit: findings from an Ontario survey. Can J Public Health 2006;97:191–6. 16827404 10.1007/BF03405582 PMC6976240

[23] Alonso-Marsden S , Dodge KA , O'Donnell KJ , et al . Family risk as a predictor of initial engagement and follow-through in a universal nurse home visiting program to prevent child maltreatment. Child Abuse Negl 2013;37:555–65. 10.1016/j.chiabu.2013.03.012 23660409 PMC3760480

[24] Chartier MJ , Brownell MD , Isaac MR , et al . Is the families first home visiting program effective in reducing child maltreatment and improving child development? Child Maltreat 2017;22:121–31. 10.1177/1077559517701230 28413917 PMC5802547

[25] Corbacho B , Bell K , Stamuli E , et al . Cost-Effectiveness of the family nurse partnership (FNP) programme in England: evidence from the building blocks trial. J Eval Clin Pract 2017;23:1367–74. 10.1111/jep.12799 28799197

[26] Bell K , Corbacho B , Ronaldson S , et al . Costs and consequences of the family nurse partnership (FNP) programme in England: evidence from the building blocks trial. F1000Res 2019;8:1640. 10.12688/f1000research.20149.1 31632654 PMC6784875

[27] 0-5 Transfer Team . Transfer of 0-5 children’s public health commissioning to Local Authorities: Equality Analysis, 2015. Available: https://assets.publishing.service.gov.uk/government/uploads/system/uploads/attachment_data/file/417429/Equality_analysis.pdf [Accessed Jul 2021].

[28] Lawlor D , Shaw M , Johns S . Teenage pregnancy is not a public health problem. BMJ 2001;323:1428–9. 10.1136/bmj.323.7326.1428 PMC1121872 11744574

[29] Crawford C , Cribb J , Kelly E . Teenage pregnancy in England, 2013. Available: https://www.ifs.org.uk/caytpubs/caytreport06.pdf [Accessed Dec 2020].

[30] Wellings K , Palmer MJ , Geary RS , et al . Changes in conceptions in women younger than 18 years and the circumstances of young mothers in England in 2000–12: an observational study. The Lancet 2016;388:586–95. 10.1016/S0140-6736(16)30449-4 PMC4976101 27229190

[31] Bellis MA , Hughes K , Leckenby N , et al . National household survey of adverse childhood experiences and their relationship with resilience to health-harming behaviors in England. BMC Med 2014;12:72. 10.1186/1741-7015-12-72 24886026 PMC4234527

[32] Mejdoubi J , van den Heijkant SCCM , van Leerdam FJM , et al . Effects of nurse home visitation on cigarette smoking, pregnancy outcomes and breastfeeding: a randomized controlled trial. Midwifery 2014;30:688–95. 10.1016/j.midw.2013.08.006 24041564

[33] Kitzman H , Olds DL , Henderson CR , et al . Effect of prenatal and infancy home visitation by nurses on pregnancy outcomes, childhood injuries, and repeated childbearing. A randomized controlled trial. JAMA 1997;278:644–52. 10.1001/jama.1997.03550080054039 9272896

[34] Olds DL , Robinson J , O'Brien R , et al . Home visiting by paraprofessionals and by nurses: a randomized, controlled trial. Pediatrics 2002;110:486–96. 10.1542/peds.110.3.486 12205249

[35] Olds DL , Holmberg JR , Donelan-McCall N , et al . Effects of home visits by paraprofessionals and by nurses on children: follow-up of a randomized trial at ages 6 and 9 years. JAMA Pediatr 2014;168:114–21. 10.1001/jamapediatrics.2013.3817 24296904 PMC4217160

[36] Olds DL , Kitzman H , Hanks C , et al . Effects of nurse home visiting on maternal and child functioning: age-9 follow-up of a randomized trial. Pediatrics 2007;120:e832–45. 10.1542/peds.2006-2111 17908740 PMC2839449

[37] Olds DL , Eckenrode J , Henderson CR , et al . Long-Term effects of home visitation on maternal life course and child abuse and neglect. Fifteen-year follow-up of a randomized trial. JAMA 1997;278:637–43. 9272895

[38] Feder GS , Hutson M , Ramsay J , et al . Women exposed to intimate partner violence: expectations and experiences when they encounter health care professionals: a meta-analysis of qualitative studies. Arch Intern Med 2006;166:22–37. 10.1001/archinte.166.1.22 16401807

[39] McTavish JR , Kimber M , Devries K , et al . Children's and caregivers' perspectives about mandatory reporting of child maltreatment: a meta-synthesis of qualitative studies. BMJ Open 2019;9:e025741. 10.1136/bmjopen-2018-025741 PMC6500368 30948587

[40] Lewis NV , Feder GS , Howarth E , et al . Identification and initial response to children's exposure to intimate partner violence: a qualitative synthesis of the perspectives of children, mothers and professionals. BMJ Open 2018;8:e019761. 10.1136/bmjopen-2017-019761 PMC5931305 29705757

[41] Cavallaro FL , Gilbert R , Wijlaars L , et al . Evaluating the real-world implementation of the family nurse partnership in England: protocol for a data linkage study. BMJ Open 2020;10:e038530. 10.1136/bmjopen-2020-038530 PMC7239518 32430455

[42] Sanders J , Channon S , Gobat N , et al . Implementation of the family nurse partnership programme in England: experiences of key health professionals explored through trial parallel process evaluation. BMC Nurs 2019;18:13. 10.1186/s12912-019-0338-y 30976196 PMC6444391

[43] Dheensa S . Recording and sharing information about domestic violence/abuse in the health service, 2020. Available: https://www.standingtogether.org.uk/blog-3/recording-and-sharing-information-about-dva-in-the-health-service-report [Accessed Feb 2021].

