# Supplementary material for: Characteristics of enrolment in an intensive home-visiting programme among eligible first-time adolescent mothers in England: a linked administrative data cohort study
Source: J Epidemiol Community Health. 2022 Oct 5;76(12):991–8. doi: 10.1136/jech-2021-217986 (PMC9664100; doi:10.1136/jech-2021-217986)
Supplement: Supplementary data [file jech-2021-217986supp001.pdf]

Supplementary Materials 1

Figure S1. Number of years Family Nurse Partnership site active within the study period (April 2010-March 2017), by English Local Authority

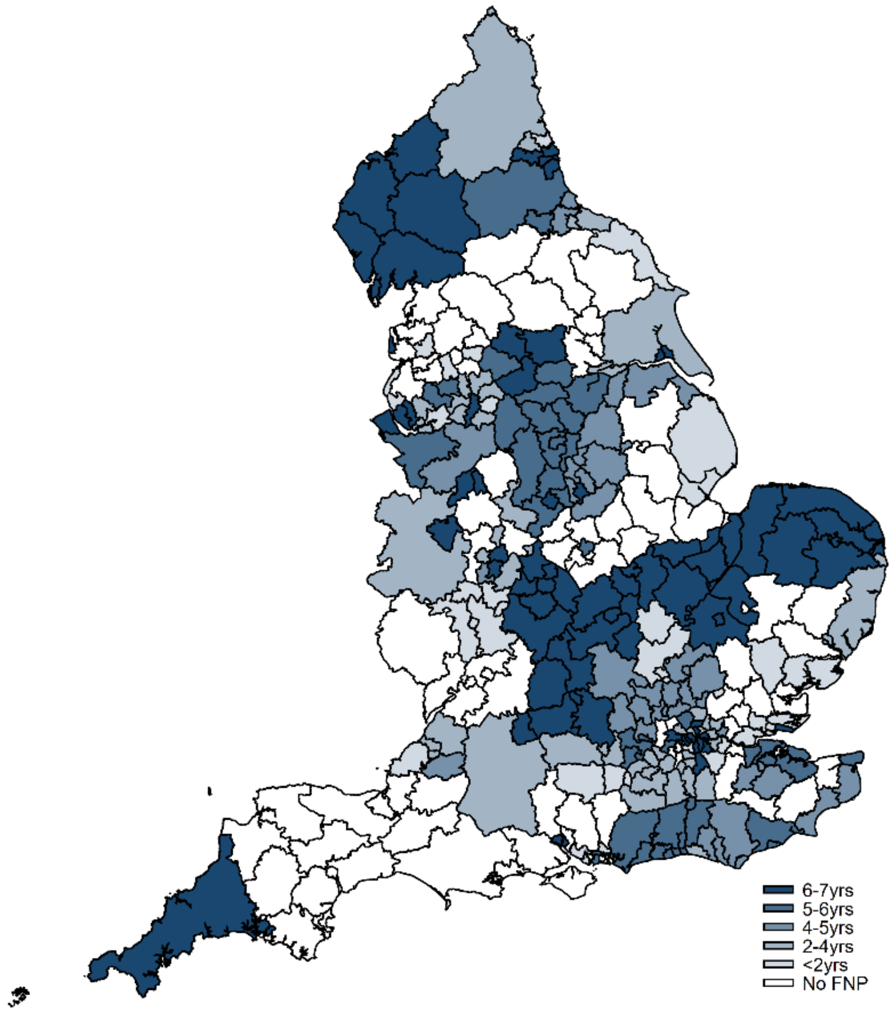

Figure S2. Adjusted odds ratios for FNP enrolment and 95% confidence intervals for maternal age, ethnicity and area-level deprivation, by English region – mothers aged 13-19, giving birth between 1 April 2010 and 31 March 2017

A. Maternal age

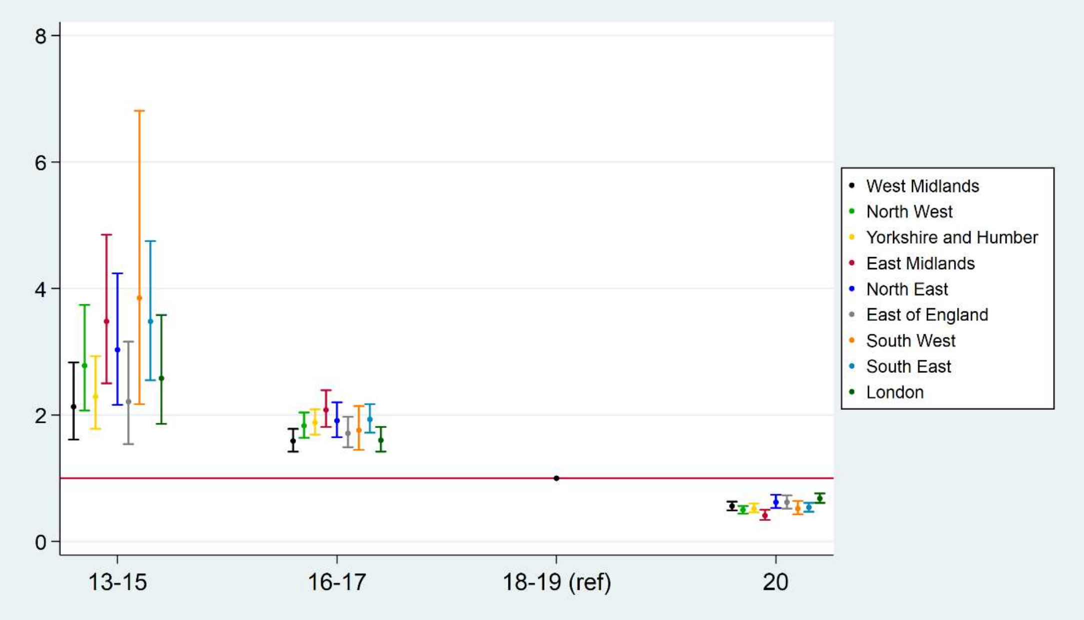

B. Ethnicity

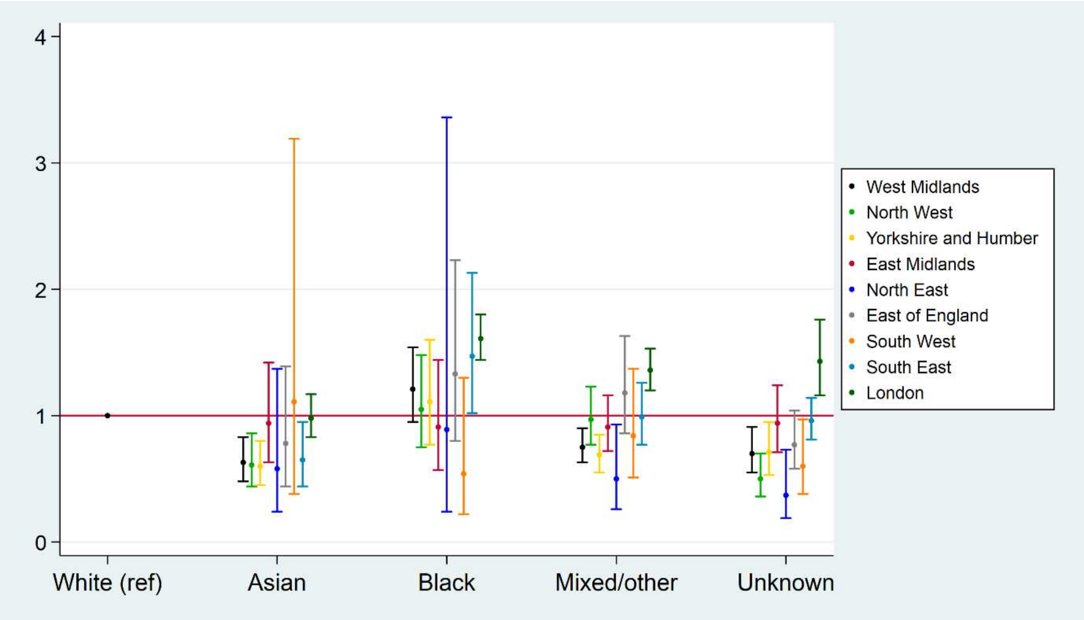

C. Area-level deprivation

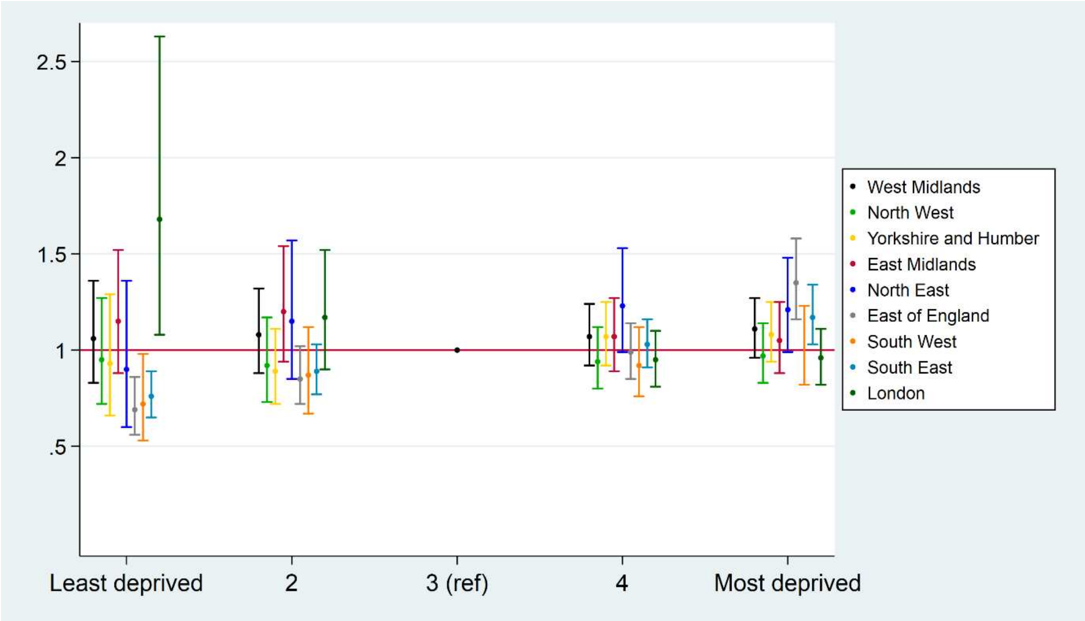

Figure S3. Adjusted funnel plots of variation in FNP enrolment among eligible first-time teenage mothers across FNP sites, by maternal age at birth – England, births between April 2010-March 2017

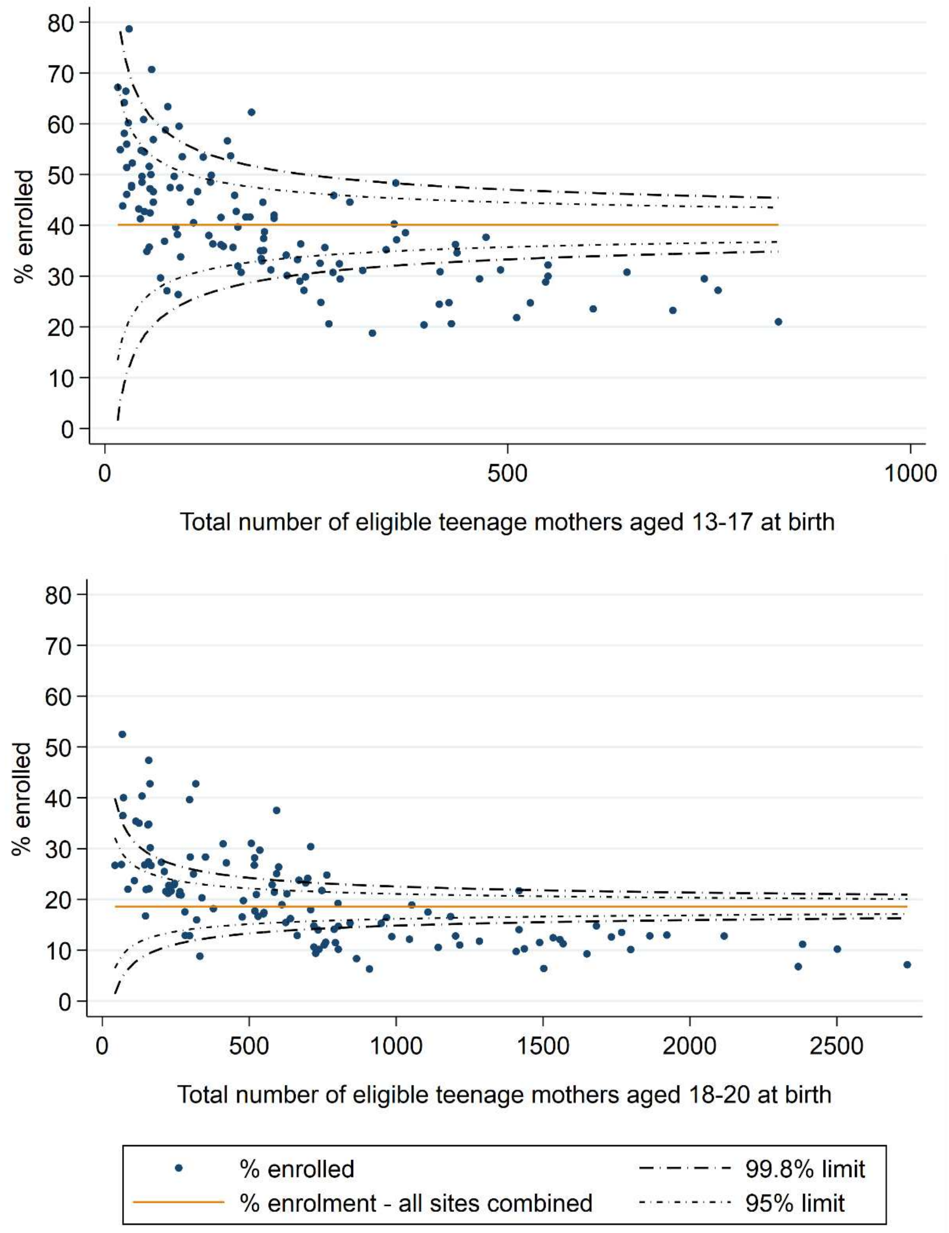

Note: adjusted percentages of eligible mothers enrolled adjust for all maternal characteristics (maternal age at birth, ethnicity, area-level deprivation quintile, any unplanned mental health-related, adversity-related, and chronic condition-related hospital admission in 2 years before 20 weeks gestation, and any A&E visit in 2 years before 20 weeks gestation)

Table S1. ICD-10 code lists for risk factors relating to adversity, mental health, and chronic conditions

Code lists were derived from the following studies:

Harron K, Gilbert R, Fagg J, Guttmann A, van der Meulen J. Associations between pre-pregnancy psychosocial risk factors and infant outcomes: a population-based cohort study in England. *The Lancet Public Health* 2021; 6(2): e97-e105.

Herbert A, Gilbert R, González-Izquierdo A, et al. Violence, self-harm and drug or alcohol misuse in adolescents admitted to hospitals in England for injury: a retrospective cohort study. BMJ Open. 2015;5(2):e006079.

Hardelid P, Dattani N, Gilbert R. Estimating the prevalence of chronic conditions in children who die in England, Scotland and Wales: a data linkage cohort study. BMJ Open. 2014;4(8).

Pearson RJ, Jay MA, Wijlaars LPMM, et al. Association between health indicators of maternal adversity and the rate of infant entry to local authority care in England: a longitudinal ecological study. *BMJ Open* 2020; 10(8): e036564.

Group	Description	ICD10 Code
**Adversity-related admissions**		
Violence	Maltreatment syndromes	T74
	Effects of other deprivation (extreme neglect)	T73
	Perpetrator of neglect and other maltreatment syndromes	Y06, Y07
	Assault by bodily force and sexual assault	Y04, Y05
	Other types of assault	X85-Y03, Y08-Y09
	Events of undetermined intent	Y20-Y34
	Examination and observation following other inflicted injury	Z04.5
	Examination and observation for other reasons: request for expert evidence	Z04.8
Self-harm	Sequelae of intentional self-harm	Y87.0
	Intentional self-poisoning by and exposure to … drugs	X60-X63
	…other and unspecified drugs, medicaments and biological substances	X64
	…alcohol	X65
	…organic solvents and halogenated hydrocarbons and their vapours	X66
	…other gases and vapours	X67
	…pesticides	X68
	…other and unspecified chemicals and noxious substances	X69
	Intentional self-harm by… hanging, strangulation and suffocation	X70
	…drowning and submersion	X71
	…firearm discharge	X72-X74
	…explosive material	X75
	…smoke, fire and flames, or steam, hot vapours and hot objects	X76-X77
	…sharp/blunt objects	X78-X79
	...jumping from a high place	X80
	…jumping or lying before a moving object, or crashing a motor vehicle	X81-82
	…other specified means	X83
	…unspecified means	X84
Substance misuse	Mental and behavioural disorders due to psychoactive substance use	F11-F16, F19
	Finding of drugs not normally found in blood	R78.1-R78.5

	Poisoning by drugs, medicaments and biological substances	T36-T50 (not T50.6)
	Poisoning, undetermined intent	Y10-Y14
	Drug rehabilitation	Z50.3
	Drug abuse counselling and surveillance	Z71.5
	Drug use	Z72.2
	Mental and behavioural disorders due to use of volatile solvents	F18
	Accidental poisoning by and exposure to noxious substances	X40–X44, X46-X49
	Poisoning by chemical or noxious substance, undetermined intent	Y16-Y19
	Special epileptic syndromes - (related to alcohol, drugs, etc.)	G40.5
	Blood-alcohol and blood-drug test	Z04.0
	Alcohol-induced pseudo-Cushing's syndrome	E24.4
	Mental and behavioural disorders due to use of alcohol	F10
	Degeneration of nervous system due to alcohol	G31.2
	Alcoholic polyneuropathy	G62.1
	Alcoholic myopathy	G72.1
	Alcoholic cardiomyopathy	I42.6
	Alcoholic gastritis	K29.2
	Alcoholic liver disease	K70
	Alcohol-induced acute pancreatitis	K85.2
	Alcohol-induced chronic pancreatitis	K86.0
	Maternal care for (suspected) damage to fetus from alcohol	O35.4
	Finding of alcohol in blood	R78.0
	Poisoning: antidotes and chelating agents, not elsewhere classified	T50.6
	Toxic effect of alcohol	T51
	Accidental poisoning by exposure to alcohol	X45
	Poisoning by exposure to alcohol, undetermined intent	Y15
	Evidence of alcohol involvement determined by blood alcohol level	Y90
	Evidence of alcohol involvement determined by level of intoxication	Y91
	Alcohol rehabilitation	Z50.2
	Alcohol abuse counselling and surveillance	Z71.4
	Alcohol use	Z72.1
**Mental health conditions / behavioural disorders (excluding those falling under adversity or chronic conditions)**		
	Organic, including symptomatic, mental disorders	F00-F09*
	Schizophrenia, schizotypal and delusional disorders	F20-F29
	Mood [affective] disorders	F30-F39
	Neurotic, stress-related and somatoform disorders	F40-F48
	Behavioural syndromes associated with physiological disturbances and physical factors	F50-F59**
	Disorders of adult personality and behaviour	F60-F69***
	Mental retardation	F70-F79
	Disorders of psychological development	F80-F89
	Behavioural and emotional disorders with onset usually occurring in childhood and adolescence	F90-F98
	Sedatives, hypnotics and antianxiety drugs	Y47
	Psychotropic drugs, not elsewhere classified	Y49
**Chronic conditions (except those falling under mental health conditions)**		
Cancer/blood disorders	Neoplasms	C00-C97, D00-D02, D05-D09, D12, D13, D14.1-D14.4, D15, D20, D32-D35, D37-D48, D63.0, E34.0, E88.3, G13.0, G13.1, G53.3, G55.0, G63.1, G73.1, G73.2, G94.1, M36.0, M36.1, M49.5, M82.0, M90.6, M90.7, N08.1, N16.1, Y43.1-Y43.3, Y84.2, Z08, Z51.0-Z51.2, Z54.1, Z54.2, Z85, Z86.0, Z92.3

	Immunological disorders	D80-D84, G53.2, Q98.0
	Anaemia and other blood disorders	D50, D56.0-D56.2, D56.4, D56.8, D56.9, D57.0-D57.2, D57.8, D58, D61.0, D61.9, D64, D66, D67, D68.0-D68.2, D68.4-D68.9, D69, D70-D76, M36.2-M36.4, M90.4, N08.2, Z86.2
Chronic infections	HIV	B20-B24, F02.4, R75, Z21
	Tuberculosis	A15-A19, E35.0, K23.0, K67.3, K93.0, M01.1, M49.0, P37.0
	Other	A50, A81, B18, B37.1, B37.5, B37.6, B37.7, B38.1, B39.1, B40.1, B44.0, B44.7, B45, B46, B48.7, B50.0, B50.8, B51.0, B51.8, B52.8, B52.0, B55, B57.2-B57.5, B58.0, B59, B67, B69, B73, B74, B78.7, B90-B94, F02.1, K23.1, K93.1, M00, N33.0, P35.0- P35.2, P35.8, P35.9, P37.1
Respiratory	Asthma and chronic lower respiratory disease	J41-J47
	Cystic fibrosis	E84, P75
	Injuries	S17, S27, S28, T27, T91.4
	Congenital anomalies	Q30-Q37, Q79.0
	Other	G47.3, J60-J70, J80-J86, J96.1, J98, P27, Y55.6, Z43.0, Z93.0, Z94.2
Metabolic/endocrine /digestive /renal /genitourinary	Diabetes	E10-E14, G59.0, G63.2, I79.2, M14.2, N08.3, O24, Y42.3
	Other endocrine	E00, E03.0, E03.1, E07.1, E22.0, E23.0, E25, E26.8, E29.1, E31, E34.1, E34.2, E34.5, E34.8, G13.2, G73.5, Y42.1
	Digestive	K20, K21.0, K22, K23.8, K25-K28, K29.0, K29.1, K29.3-K29.9, K31, K50-K52, K55, K57, K59.2, K63.0-K63.3, K66, K72-K76, K80-K83, K85.0, K85.1, K85.8, K85.9, K86.1-K86.9, K87.0, K90, M07.4, M07.5, M09.1, M09.2, T86.4, Z43.2-Z43.4, Z46.5, Z90.3, Z90.4, Z93.2-Z93.5
	Renal/genitourinary	D63.8, G63.8, G99.8, I68.8, M90.8, N08.4, N00-N05, N07, N11-N15, N16.0, N16.2, N16.4, N16.5, N16.8, N18, N19, N20-N23, N25, N26, N28, N29, N31, N32, N33.8, N35, N36, N39.1, N39.3, N39.4, N40-N42, N70-N74, N80-N82, N85, N86, N87,N88, P96.0, T82.4, T83.1, T83.2, T83.4-T83.9, T85.5, T86.1, Y60.2, Y61.2, Y62.2, Y84.1, Z49, Z93.6, Z94.0, Z99.2
	Congenital anomalies of the digestive/renal/genitourinary system	Q38.0, Q38.3, Q38.4, Q38.6-Q38.8, Q39, Q40.2, Q40.3, Q40.8, Q40.9, Q41, Q42, Q43.1, Q43.3-Q43.7, Q43.9, Q44, Q45, Q50.0, Q51, Q52.0-Q52.2, Q52.4, Q54.0-Q54.3, Q54.8, Q54.9, Q55.0, Q55.5, Q56, Q60.1, Q60.2, Q60.4-Q60.6, Q61, Q62.0-Q62.6, Q62.8, Q63.0-Q63.2, Q63.8, Q63.9, Q64, Q79.2-Q79.5, Q87.8, Q89.1, Q89.2
	Injuries	S36, S37, S38, S39.6, S39.7, T06.5, T28, T91.5
	Other/unspecified	E66, G63.3, G99.0, M14.5, N92, Z86.3, Z93.8
Musculoskeletal/ skin	Musculoskeletal/connective tissue	G55.1-G55.3, G63.5, G63.6, G73.7, J99.0, J99.1, L62.0, M05, M06, M07.0-M07.3, M07.6, M08, M09.8, M10-M13, M14.0, M14.6, M14.8, M30-M35, M40-M43, M45-M48,M50-M54, M60-M62, M63.8, M80.1-M80.9, M81.1-M81.9, M82.1, M82.8, M84.0-M84.2, M84.8, M84.9, M85, M86.3-M86.6, M89, M90.0, M91-M94, N08.5, Y45.4
	Skeletal injuries/amputations	S13, S22.0-S22.2, S22.5, S23, S32, S33, S68.3, S68.4, S68.8, S77, S78, S87, S88, S97, S98.0, S98.2-S98.4, T02, T04, T05, T20.3, T20.7, T21.3, T21.7, T22.3, T22.7, T23.2, T23.3, T23.6, T23.7, T24.3, T24.7, T25.2, T25.3, T25.6, T25.7, T29.3, T29.7, T30.3, T30.7, T31.2-T31.9, T32.2- T32.9, T87.3-T87.6, T91.2 T91.8, T92.6, T93.1, T93.4, T93.6, T94.0, T94.1, T95.0, T95.1,

		T95.4, T95.8, T95.9, Y83.5, Z89.1, Z89.2, Z89.5-Z89.8, Z97.1
	Chronic skin disorders	L10, L11.0, L11.8, L11.9, L12-L14, L28, L40-L45, L57, L58.1, L59, L87, L88, L90, 3 L92, L95, L93, L98.5, M09.0, Q80, Q81, Q87.0-Q87.5, Q89.4
	Congenital anomalies	Q18.8, Q65.0-Q65.2, Q65.8, Q65.9, Q67.5, Q68.2, Q68.3-Q68.5, Q71-Q73, Q74, Q75.3-Q75.9, Q76.1-Q76.4, Q77, Q78, Q79.6, Q79.8, Q82.0-Q82.4, Q82.9, Q86.2, Q89.7-Q89.9
Neurological	Epilepsy	F80.3, G40.0-G40.4, G40.6-G40.9, G41, R56.8, Y46.0-Y46.6
	Cerebral palsy	G80-G83
	Injuries of brain, nerves, eyes or ears	S05-S08, S12, S14, S24, S34, S44, S54, S64, S74, S84, S94, T06.0- T06.2, T26, T90.4, T90.5, T91.1, T91.3, T92.4
	Chronic eye conditions	H05.1-H05.9, H13.3, H17, H18, H19.3, H19.8, H21, H26, H27, H28.0-H28.2, H31, H32.8, H33, H34, H35, H40, H42.0, H43, H44, H47, H54.0- H54.2, H54.4, T85.2, T85.3, Z44.2
	Chronic ear conditions	H60.2, H65.2-H65.4, H66.1-H66.3, H69.0, H70.1, H73.1, H74.0-H74.3, H75.0, H80, H81.0, H81.4, H83.0, H83.2, H90.0, H90.3, H90.5, H90.6, H91, Z45.3
	Perinatal conditions	P10, P21.0, P52, P57, P90, P91.1, P91.2, P91.6
	Congenital anomalies of neurological or sensory systems	Q00-Q07, Q10.4, Q10.7, Q11-Q12, Q13.0-Q13.4, Q13.8, Q13.9, Q14-Q16, Q75.0, Q75.1, Q85, Q86.0, Q86.1, Q86.8, Q90-Q93, Q95.2, Q95.3, Q97, Q99
	Other	F02.2, F02.3,G00-G09, G10-G12, G13.8, G14, G20-G23, G24.1-G24.9, G25-G30, G31.0-G31.1, G31.8, G31.9, G32-G37, G43-G46, G47.0-G47.2, G47.4-G47.9, G50- G52, G53.0, G53.1, G53.8, G54, G55.8, G56-G58, G59.8, G60, G61, G62.0, G62.2- G62.9, G64, G70, G71,G72.2-G72.9, G73.0, G73.3, G90-G93, G94.2, G94.8, G95, G96, G98, G99.1, G99.2, I60-I67, I68.0, I68.2, I69, I72.0, I72.5, T85.0, T85.1, Y46.7- Y46.8, Z98.2
Cardiovascular	Congenital heart disease	Q20-Q26, Q89.3
	Other	I00-I28, I31-I39, I41, I42.0-I42.5, I42.7-I42.9, I43.0, I43.1, I43.2-I43.8, I44.1-I44.7, I45.1-I45.9, I46-I51, I52.8, I70-I71, I72.1-I72.4, I72.8, I72.9, I73-I77, I79.0, I79.1, I79.8, I81-I82, I98-I99, M03.6, N08.8, Q27, Q28, S26, T82.0-T82.3, T82.5-T82.9, T86.2, Y60.5, Y61.5, Y62.5, Y84.0, Z45.0, Z50.0, Z94.1, Z95
Codes indicating non-specific chronic conditions	-	R62, R63.3, Z43.1, Z51.5, Z75.5, Z93.1, Z99.3

* Excluding F020-F024 (dementia codes)
** Excluding F51 (nonorganic sleep disorders) and F52 (sexual dysfunction, not caused by organic disorder or disease)
*** Excluding F64 (gender identity disorders (including transsexualism, transvestism, and “gender disorders”)

Table S2. Description of enrolment in the Family Nurse Partnership among first-time mothers aged 13-19 at last menstrual period by FNP site – England, births between April 2010-March 2017

FNP site name	Local authority(ies)	Start date*	End date*	N enrolled	N eligible	% enrolled	Median monthly caseload	Maximum monthly caseload
**Total**	**-**	**-**	**-**	**25,675**	**110,520**	**23.2**	**-**	**-**
**North East**								
Durham & Darlington	County Durham, Darlington,	2009m8	2015m10	560	3,065	18.3	143	212
Gateshead	Gateshead,	2010m2	2019m3	185	885	20.9	56	71
Hartlepool	Hartlepool, Stockton-on-Tees,	2012m2<br>2012m4	2017m2<br>2017m12	225	985	22.8	80	111
Middlesbrough	Middlesbrough, Redcar and Cleveland,	2012m3<br>2012m4	2015m11<br>2018m3	180	960	18.8	51	107
Newcastle upon Tyne	Newcastle-upon-Tyne,	2014m4	2016m9	90	380	23.7	60	79
North Tyneside	North Tyneside,	2014m3	2016m3	90	195	46.2	56	75
Northumberland	Northumberland,	2014m3	2016m9	90	360	25	58	76
South Tyneside	South Tyneside,	2010m1	2018m1	215	815	26.4	60	86
Sunderland	Sunderland,	2009m8	2019m3	315	1,615	19.5	94	122
**North West**								
Blackburn with Darwen	Blackburn with Darwen,	2015m5	2016m4	55	100	55	34	57
Blackpool	Blackpool,	2009m8	2019m3	245	1,045	23.4	68	133
Bolton	Bolton,	2011m11	2019m3	240	880	27.3	94	123
Bury	Bury,	2014m12	2019m3	80	160	50	54	73
Cheshire East	Cheshire East,	2012m9	2019m3	210	530	39.6	94	114
Cheshire West	Cheshire West & Chester,	2012m2	2019m3	215	675	31.9	75	123
Cumbria	Allerdale, Barrow-in-Furness, Carlisle, Copeland, Eden, South Lakeland,	2009m8	2016m8	205	1,875	10.9	53	86
Halton	Halton,	2014m7	2019m3	90	220	40.9	64	87
Knowsley	Knowsley,	2009m8	2019m3	255	740	34.5	62	95
Lancashire	Burnley, Preston,	2015m4	2017m4	105	215	48.8	80	100
Liverpool	Liverpool,	2009m8	2019m3	430	1,965	21.9	116	149
Manchester	Manchester,	2009m8	2016m9	530	2,540	20.9	146	192
Oldham	Oldham,	2014m8	2019m3	95	310	30.6	61	88
Rochdale	Rochdale,	2014m8	2017m2	95	275	34.5	52	84
Salford	Salford,	2014m12	2019m3	85	250	34	63	78
Sefton	Sefton,	2014m12	2016m9	45	170	26.5	24	37
St Helens	St Helens,	2014m12	2019m3	105	200	52.5	44	89
Stockport	Stockport,	2014m8	2019m3	100	185	54.1	52	88
Tameside	Tameside,	2015m3	2019m3	70	195	35.9	62	79
Trafford	Trafford,	2015m1	2017m3	20	65	30.8	26	30

FNP site name	Local authority(ies)	Start date*	End date*	N enrolled	N eligible	% enrolled	Median monthly caseload	Maximum monthly caseload
Warrington	Warrington,	2015m4	2019m3	75	145	51.7	69	79
Wigan	Wigan,	2011m12	2019m3	205	895	22.9	79	116
Wirral	Wirral,	2009m8	2019m3	345	1,335	25.8	103	125
**Yorkshire and Humber**								
Barnsley	Barnsley,	2009m8	2015m11	275	970	28.4	89	107
Bradford & Airedale	Bradford,	2010m2	2019m3	430	2,415	17.8	110	183
Calderdale	Calderdale,	2009m8	2016m2	155	935	16.6	51	84
Doncaster	Doncaster,	2009m8	2016m3	285	1,850	15.4	73	128
East Riding	East Riding of Yorkshire,	2013m6	2019m3	60	400	15	33	60
Hull	Kingston upon Hull, city of,	2009m8	2017m6	345	2,060	16.7	103	127
Kirklees	Kirklees,	2009m8	2019m3	345	1,950	17.7	80	138
Leeds	Leeds,	2009m8	2016m4	400	3,360	11.9	127	171
North & North East Lincolnshire	North East Lincolnshire, North Lincolnshire,	2012m3	2017m4	175	1,130	15.5	69	88
North Yorkshire	Scarborough,	2013m6	2014m12	40	110	36.4	16	35
Rotherham	Rotherham,	2011m11	2016m12	200	920	21.7	69	93
Sheffield	Sheffield,	2009m8	2016m2	380	2,135	17.8	95	191
Wakefield	Wakefield,	2011m11	2019m3	290	1,235	23.5	106	147
**East Midlands**								
Derby City	Derby,	2009m8	2019m3	415	1,350	30.7	106	185
Derbyshire	Amber Valley, Bolsover, Chesterfield, Derbyshire Dales, Erewash, High Peak, North East Derbyshire, South Derbyshire,	2011m10	2019m3	355	1,775	20	95	166
Leicester City	Leicester,	2011m9	2017m5	210	970	21.6	71	95
Lincolnshire	Boston, East Lindsey,	2014m8	2016m2	65	200	32.5	47	58
Northamptonshire	Corby, Daventry, East Northamptonshire, Kettering, Northampton, South Northamptonshire, Wellingborough,	2009m8	2019m3	375	3,000	12.5	96	129
Nottingham City	Nottingham,	2009m8	2019m3	435	1,815	24	124	179
Nottinghamshire	Ashfield, Bassetlaw, Broxtowe, Gedling, Mansfield, Newark and Sherwood, Rushcliffe,	2012m10	2019m3	370	1,500	24.7	170	294
**West Midlands**								
Birmingham	Birmingham,	2013m1	2016m5	450	2,235	20.1	89	238

FNP site name	Local authority(ies)	Start date*	End date*	N enrolled	N eligible	% enrolled	Median monthly caseload	Maximum monthly caseload
Coventry	Coventry,	2009m8	2019m3	290	1,610	18	87	115
Dudley	Dudley,	2013m2	2019m3	240	645	37.2	108	139
East Staffordshire	East Staffordshire,	2013m3	2017m4	85	210	40.5	30	44
Sandwell	Sandwell,	2009m8	2017m1	395	2,020	19.6	84	167
Shropshire	Shropshire,	2014m11	2019m3	95	175	54.3	69	106
Solihull	Birmingham, Solihull,	2009m8	2019m3	290	855	33.9	80	115
Staffordshire - Cannock and Tamworth - Closed	Cannock Chase, Tamworth,	2013m3	2016m8	155	380	40.8	74	106
Stoke-on-Trent	Newcastle-under-Lyme, Stoke-on-Trent,	2009m8	2016m10	325	2,155	15.1	109	170
Telford & Wrekin	Telford and Wrekin,	2009m8	2019m3	215	975	22.1	54	76
Walsall	Walsall,	2009m8	2016m9	340	1,835	18.5	100	126
Warwickshire North	North Warwickshire, Nuneaton and Bedworth,	2010m11	2019m3	200	765	26.1	49	94
Warwickshire South & Rugby	Rugby, Stratford-on-Avon, Warwick,	2010m11	2019m3	175	790	22.2	58	82
Wolverhampton	Wolverhampton,	2014m11	2017m7	90	295	30.5	63	82
Worcestershire	Bromsgrove, Malvern Hills, Redditch, Worcester, Wychavon, Wyre Forest,	2015m9	2018m4	95	315	30.2	60	91
**East of England**								
Bedford & Bedfordshire	Bedford, Central Bedfordshire,	2015m1	2017m1	90	265	34	44	81
Hertfordshire	Broxbourne, Dacorum, East Hertfordshire, Hertsmere, North Hertfordshire, St Albans, Stevenage, Three Rivers, Watford, Welwyn Hatfield,	2011m9	2016m2	215	1,455	14.8	74	104
Luton	Luton,	2015m9	2017m8	60	140	42.9	38	63
Norfolk	Breckland, Broaland, Great Yarmouth, King's Lynn and West Norfolk, North Norfolk, Norwich, South Norfolk,	2010m1	2019m3	545	3,140	17.4	155	210
North Essex	Braintree, Colchester, Harlow, Tendring,	2014m11	2016m11	135	430	31.4	72	103
Peterborough & Cambridgeshire	Cambridge, East Cambridgeshire, Fenland, Huntingdonshire, Peterborough, South Cambridgeshire,	2010m1 2011m12	2019m3	440	2,375	18.5	142	182
South-East Essex	Basildon, Castle Point, Rochford, Thurrock,	2009m8 2015m2	2016m10 2017m2	145	585	24.8	27	65
Southend	Southend-on-Sea,	2009m8	2019m3	170	625	27.2	45	67

FNP site name	Local authority(ies)	Start date*	End date*	N enrolled	N eligible	% enrolled	Median monthly caseload	Maximum monthly caseload
Suffolk	Ipswich, Suffolk Coastal, Waveney,	2010m10<br>2013m8	2018m3<br>2018m5<br>2019m3	285	870	32.8	91	133
**London**								
Barking & Dagenham	Barking & Dagenham,	2010m11	2015m11	185	655	28.2	45	71
Barnet	Barnet,	2011m11	2019m3	175	360	48.6	60	80
Brent	Brent,	2014m6	2016m7	75	270	27.8	38	49
Bromley & Bexley	Bexley, Bromley,	2014m9<br>2014m10	2016m8<br>2019m3	90	270	33.3	49	61
Camden	Camden,	2015m1	2018m7	30	55	54.5	10	19
Croydon	Croydon,	2010m10	2019m3	200	1,010	19.8	69	97
Ealing	Ealing,	2009m8	2019m3	220	720	30.6	55	70
Enfield	Enfield,	2013m11	2019m3	110	385	28.6	46	78
Greenwich	Greenwich,	2014m4	2019m3	110	360	30.6	70	86
Hackney	Hackney,	2014m5	2019m3	85	300	28.3	41	54
Haringey	Haringey,	2010m7	2019m3	200	725	27.6	58	91
Hounslow	Hounslow,	2011m7	2019m3	155	435	35.6	53	76
Islington	Islington,	2009m8	2019m3	175	470	37.2	34	63
Lambeth	Lambeth,	2009m8	2019m3	270	950	28.4	72	107
Lewisham	Lewisham,	2010m1	2019m3	280	950	29.5	84	105
Merton	Merton,	2013m9	2019m3	45	135	33.3	25	33
Newham	Newham,	2013m9	2019m3	90	425	21.2	47	68
Southwark	Southwark,	2009m8	2019m3	270	840	32.1	73	86
Sutton	Sutton,	2014m1	2017m6	60	160	37.5	25	45
Tower Hamlets	Tower Hamlets,	2009m8	2019m3	275	645	42.6	79	109
Waltham Forest & Redbridge	Redbridge, Waltham Forest,	2009m8<br>2013m10	2019m3	220	1,085	20.3	48	73
Wandsworth	Wandsworth,	2015m5	2019m3	65	95	68.4	49	73
West Central London	Hammersmith and Fulham, Kensington and Chelsea, Westminster,	2010m2	2019m3	220	725	30.3	47	70
**South East**								
Berkshire East	Bracknell Forest, Slough, Windsor and Maidenhead,	2009m8	2016m2	335	855	39.2	96	114
Berkshire West	Reading, West Berkshire, Wokingham,	2012m11	2016m6	170	610	27.9	66	84
Brighton & Hove	Brighton & Hove,	2012m10	2016m3	125	270	46.3	52	89
Buckinghamshire	Aylesbury Vale, Chiltern, South Buckinghamshire, Wycombe,	2012m7	2019m3	225	620	36.3	93	128

FNP site name	Local authority(ies)	Start date*	End date*	N enrolled	N eligible	% enrolled	Median monthly caseload	Maximum monthly caseload
East Sussex - East	Hastings, Rother,	2009m8	2015m11<br>2015m12	245	795	30.8	72	107
East Sussex - West	Eastbourne, Lewes, Wealden,	2010m11<br>2011m3<br>2011m12	2015m6<br>2015m11<br>2015m12	250	620	40.3	95	118
Hampshire	Basingstoke and Deane, Fareham, Gosport, Hart, Havant, Rushmoor,	2015m3<br>2015m4<br>2015m8<br>2016m1	2017m4<br>2018m3<br>2018m8<br>2019m3	190	360	52.8	145	179
Kent North	Gravesham, Swale, Thanet,	2012m2<br>2014m12	2017m3<br>2018m6	215	1,045	20.6	83	101
Kent South	Dover, Maidstone, Shepway, Tonbridge and Malling,	2013m2<br>2014m12	2017m10<br>2018m6	170	660	25.8	58	101
Medway	Medway,	2009m8	2015m12	265	1,280	20.7	75	118
Milton Keynes	Milton Keynes,	2009m8	2016m10	165	995	16.6	42	67
Oxfordshire	Cherwell, Oxford, South Oxfordshire, Vale of White Horse, West Oxfordshire,	2010m2	2019m3	475	1,730	27.5	131	180
Portsmouth	Portsmouth,	2011m11	2019m3	290	665	43.6	111	155
Southampton	Southampton,	2009m8	2019m3	225	1,200	18.8	81	98
Surrey	Elmbridge, Epsom and Ewell, Guildford, Mole Valley, Reigate and Banstead, Runnymede, Spelthorne, Surrey Heath, Tandridge, Waverley, Woking,	2014m11	2019m3	110	420	26.2	58	83
West Sussex	Adur, Arun, Chichester, Crawley, Horsham, Mid Sussex, Worthing,	2011m9	2019m3	265	1,490	17.8	101	118
**South West**								
Bath & North East Somerset	Bath & North East Somerset,	2013m2	2019m3	100	175	57.1	51	67
Bristol & South Gloucestershire	Bristol, city of, South Gloucestershire,	2014m5	2019m3	150	560	26.8	91	124
Bristol and North Somerset	North Somerset,	2014m8	2015m8	30	80	37.5	20	27
Cornwall & Isles of Scilly	Cornwall,	2009m8	2017m11	470	2,105	22.3	130	167
Plymouth	Plymouth,	2009m8	2019m3	225	1,310	17.2	61	99
Swindon	Swindon,	2009m8	2019m3	340	920	37	96	132
Wiltshire	Wiltshire,	2014m11	2019m3	85	285	29.8	51	65

Note: numbers have been rounded to the nearest 5 in accordance with NHS Digital’s statistical disclosure rules for sub-national analyses.

Table S3. Comparison of maternal characteristics among eligible adolescent mothers living in an area with an active FNP site at the time of first antenatal appointment and those living in an area where FNP was never commissioned – England, births between March 2010-April 2017

	N eligible mothers	N enrolled in FNP	% enrolled in FNP	N mothers in Local Authorities where FNP was never commissioned (%)
**Total**	**110,520**	**25,675**	**23.2**	**100,455**
**Maternal age at birth**				
13-15	2380 (2.2)	1,240	52.1	2160 (2.2)
16-17	22725 (20.6)	8,720	38.4	20040 (19.9)
18-19	61090 (55.3)	12,875	21.1	56310 (56.1)
20[b]	24325 (22.0)	2,840	11.7	21945 (21.8)
**Ethnicity**				
White	93730 (84.8)	21,845	23.3	88895 (88.5)
South Asian	3170 (2.9)	535	16.9	2325 (2.3)
Black	3970 (3.6)	1,195	30.1	2705 (2.7)
Mixed/other	5695 (5.2)	1,335	23.4	3905 (3.9)
Unknown	3950 (3.6)	770	19.5	2620 (2.6)
**Index of Multiple Deprivation (quintile)**				
Least deprived	5550 (5.0)	1,135	20.5	8460 (8.4)
2	8565 (7.7)	1,820	21.2	12825 (12.8)
3	14835 (13.4)	3,330	22.4	18065 (18.0)
4	27520 (24.9)	6,430	23.4	25630 (25.5)
Most deprived	53905 (48.8)	12,820	23.8	34890 (34.7)
Unknown	145 (0.1)	145	100	580 (0.6)
**Admission with diagnoses within 2 years before 20 weeks pregnancy**				
Mental health (excluding substance misuse and self-harm)	2420 (2.2)	955	39.5	1950 (1.9)
Adversity-related (self-harm, substance misuse, violence)	4460 (4.0)	1,770	39.7	3910 (3.9)
Any chronic condition	9580 (8.7)	3,170	33.1	8105 (8.1)
A&E visit	68965 (62.4)	17,815	25.8	61255 (61.0)
**Gestational age at antenatal booking appointment**				
Before 10 weeks	29390 (26.6)	6,810	23.2	25840 (25.7)
10-20 weeks	40640 (36.8)	9,540	23.5	36325 (36.2)
20 weeks or more	6095 (5.5)	1,515	24.9	10180 (10.1)
Unknown	34390 (31.1)	7,815	22.7	28105 (28.0)
**Linked to NPD**				
Linked to NPD	92260 (83.5)	22,980	24.9	84385 (84.0)
Not linked to NPD	17405 (15.7)	2,570	14.8	14970 (14.9)
Linked to NPD but not to NPD census	855 (0.8)	125	14.6	1100 (1.1)
**Ever had a child protection plan or was looked after before 20 weeks pregnancy**				
No CPP or looked after	85890 (77.7)	19,860	23.1	79965 (79.6)
Looked after (CPP)	5540 (5.0)	2,445	44.1	4690 (4.7)
Child protection plan, but not looked after	1685 (1.5)	800	47.5	14970 (14.9)
**Ever recorded as having Special Educational Needs before 20 weeks pregnancy**				
No	45270 (41.0)	9,190	20.3	47740 (47.5)
Yes	46990 (42.5)	13,790	29.3	36645 (36.5)
**Ever recorded as receiving free school meals before 20 weeks pregnancy**				
No	41455 (37.5)	8,050	19.4	47570 (47.4)
Yes	50805 (46.0)	14,930	29.4	36820 (36.7)
**Ever in IDACI bottom decile before 20 weeks pregnancy**				
No	59765 (54.1)	13,760	23	65105 (64.8)

Yes	32495 (29.4)	9,220	28.4	19280 (19.2)
**Educational attainment before 20 weeks pregnancy**				
Attempted but did not achieve 5 A*-C GCSEs	69345 (62.7)	16,365	23.6	64420 (64.1)
5 A*-C GCSEs	16960 (15.3)	3,320	19.6	19280 (19.2)
Had not attempted GCSEs prior to 20 weeks pregnancy	6810 (6.2)	3,420	50.2	5785 (5.8)
**Ever excluded, in pupil referral unit, or alternative provision**				
No	65620 (59.0)	14,640	22.3	61000 (60.7)
Yes	28105 (25.2)	8,620	30.7	24485 (24.4)
**Ever persistently absent in a term (>=10% possible sessions)**				
No	58100 (52.6)	10,533	18.1	53210 (53.0)
Yes	35535 (32.2)	12,725	35.8	32275 (32.1)

Note: numbers have been rounded to the nearest 5 in accordance with NHS Digital's statistical disclosure rules for sub-national analyses.

Percentages for Ever recorded as having Special Educational Needs, Free School Meals, or in IDACI bottom decile are of those who linked to the NPD Census. Percentages for Ever had a child protection plan or child looked after, Educational attainment, Ever excluded, Ever persistently absent are of those who linked to NPD.

Table S4. Distribution of FNP sites, first-time teenage mothers enrolled in FNP, eligible mothers, and median and maximum caseloads by quartile of enrolment rates – England, births between March 2010-April 2017

Percentage of eligible mothers enrolled (quartiles)	N FNP sites	N mothers enrolled in FNP	N eligible mothers	Median monthly caseload	Median maximum monthly caseload
Low-enrolment sites (≤21%)	30	9,610	56,020	87	126
21-28%	30	6,840	28,470	67	95
28-36%	30	5,170	16,530	61	86
High-enrolment sites (36-67%)	30	4,070	9,500	58	84

Note: numbers have been rounded to the nearest 5 in accordance with NHS Digital's statistical disclosure rules for sub-national analyses.

Table S5. Predictors of enrolment in the Family Nurse Partnership in high- and low-enrolment sites, among mothers aged 13-19 at last menstrual period, living in a Local Authority with an active FNP site at the time of first antenatal appointment – England, births between April 2010-March 2017

	High enrolment sites (>=36%)					Low enrolment sites (<=21%)				
	N eligible mothers	N enrolled in FNP	% enrolled in FNP	Crude odds ratio (95% CI)	Adjusted[a] odds ratio (95% CI)	N eligible mothers	N enrolled in FNP	% enrolled in FNP	Crude odds ratio (95% CI)	Adjusted[a] odds ratio (95% CI)
**Total**	9,495	4,065	42.8	-	-	56,020	9,605	17.1	-	-
**Maternal age at birth**										
13-15	150 (1.6)	105	70	3.62 (2.53-5.19)	2.14 (1.39-3.29)	1295 (2.3)	585	45.2	4.66 (4.16-5.23)	2.60 (2.25-3.01)
16-17	1785 (18.8)	1145	64.1	2.59 (2.32-2.90)	1.92 (1.68-2.19)	11755 (21.0)	3610	30.7	2.52 (2.40-2.65)	1.78 (1.67-1.89)
18-19	5345 (56.3)	2200	41.2	1 (ref)	1 (ref)	30650 (54.7)	4610	15	1 (ref)	1 (ref)
20[b]	2215 (23.3)	615	27.8	0.53 (0.48-0.59)	0.61 (0.55-0.69)	12320 (22.0)	800	6.5	0.39 (0.36-0.42)	0.47 (0.44-0.51)
**Ethnicity**										
White	7775 (81.9)	3425	44.1	1 (ref)	1 (ref)	48735 (87.0)	8555	17.6	1 (ref)	1 (ref)
South Asian	500 (5.3)	175	35	0.61 (0.49-0.75)	0.78 (0.62-0.99)	1670 (3.0)	160	9.6	0.44 (0.37-0.52)	0.63 (0.53-0.75)
Black	310 (3.3)	135	43.5	0.98 (0.77-1.25)	1.09 (0.84-1.42)	1370 (2.4)	290	21.2	1.13 (0.99-1.30)	1.31 (1.13-1.53)
Mixed/other	475 (5.0)	190	40	0.82 (0.67-0.99)	0.93 (0.75-1.14)	2585 (4.6)	400	15.5	0.77 (0.69-0.86)	0.85 (0.75-0.95)
Unknown	435 (4.6)	140	32.2	0.57 (0.47-0.71)	0.72 (0.58-0.90)	1660 (3.0)	200	12	0.61 (0.53-0.71)	0.78 (0.66-0.91)
**Index of Multiple Deprivation (quintile)**										
Least deprived	745 (7.8)	265	35.6	0.77 (0.64-0.93)	0.80 (0.66-0.98)	2410 (4.3)	300	12.4	0.79 (0.68-0.90)	0.84 (0.73-0.97)
2	1040 (11.0)	425	40.9	0.98 (0.84-1.16)	1.00 (0.84-1.19)	4395 (7.8)	620	14.1	0.89 (0.80-0.99)	0.93 (0.83-1.03)
3	1540 (16.2)	640	41.6	1 (ref)	1 (ref)	7465 (13.3)	1160	15.5	1 (ref)	1 (ref)
4	2490 (26.2)	1080	43.4	1.10 (0.97-1.26)	1.03 (0.90-1.18)	13100 (23.4)	2210	16.9	1.11 (1.02-1.20)	1.06 (0.98-1.15)
Most deprived	3650 (38.4)	1630	44.7	1.20 (1.05-1.36)	1.07 (0.93-1.23)	28625 (51.1)	5295	18.5	1.25 (1.16-1.35)	1.12 (1.04-1.21)
Unknown	25 (0.3)	25	100	-	-	25 (0.0)	25	100	-	-
**Admission with diagnoses within 2 years before 20 weeks gestation**										
Mental health (excluding substance misuse and self-harm)	260 (2.7)	160	61.5	2.19 (1.70-2.84)	1.22 (0.89-1.68)	1170 (2.1)	365	31.2	2.25 (1.98-2.55)	1.46 (1.25-1.72)
Adversity-related	420 (4.4)	270	64.3	2.55 (2.07-3.13)	1.27 (0.96-1.68)	2245 (4.0)	705	31.4	2.33 (2.12-2.55)	1.20 (1.05-1.38)
Any chronic condition	945 (10.0)	540	57.1	1.88 (1.64-2.16)	1.30 (1.07-1.57)	4700 (8.4)	1195	25.4	1.76 (1.64-1.88)	1.14 (1.02-1.27)
A&E visits	6010 (63.3)	2805	46.7	1.55 (1.42-1.69)	1.31 (1.19-1.44)	33925 (60.6)	6525	19.2	1.47 (1.40-1.54)	1.27 (1.21-1.34)
**Gestational age at antenatal booking appointment**										
Before 10 weeks	2190 (23.1)	950	43.4	1 (ref)	1 (ref)	15375 (27.4)	2700	17.6	1 (ref)	1 (ref)
10-20 weeks	3745 (39.4)	1650	44.1	1.01 (0.91-1.13)	0.99 (0.88-1.12)	19430 (34.7)	3300	17	0.92 (0.87-0.98)	0.88 (0.82-0.93)
20 weeks or more	830 (8.7)	345	41.6	0.97 (0.82-1.16)	0.84 (0.69-1.02)	2555 (4.6)	390	15.3	0.79 (0.70-0.89)	0.63 (0.55-0.71)
Unknown	2730 (28.8)	1120	41	0.89 (0.79-1.01)	0.79 (0.69-0.91)	18660 (33.3)	3215	17.2	0.95 (0.89-1.02)	0.82 (0.77-0.88)
**Linked to NPD**										
Linked to NPD	7860 (82.8)	3600	45.8	1 (ref)	1 (ref)	47410 (84.6)	8800	18.6	1 (ref)	1 (ref)
Not linked to NPD	1555 (16.4)	440	28.3	0.44 (0.38-0.49)	0.71 (0.60-0.84)	8190 (14.6)	765	9.3	0.43 (0.40-0.46)	0.88 (0.80-0.97)
Linked to NPD but not to NPD census	85 (0.9)	20	23.5	0.38 (0.23-0.63)	0.66 (0.39-1.10)	420 (0.7)	40	9.5	0.46 (0.33-0.63)	0.78 (0.56-1.09)
**Ever had a child protection plan or was looked after before 20 weeks pregnancy**										
No CPP or looked after	7210 (75.9)	3155	43.8	1 (ref)	1 (ref)	44470 (79.4)	7595	17.1	1 (ref)	1 (ref)
Looked after (CPP)	545 (5.7)	350	64.2	2.28 (1.90-2.74)	1.63 (1.33-1.98)	2640 (4.7)	970	36.7	2.79 (2.57-3.03)	2.07 (1.89-2.26)
Child protection plan, but not looked after	190 (2.0)	120	63.2	2.06 (1.53-2.77)	1.08 (0.78-1.48)	720 (1.3)	275	38.2	3.04 (2.61-3.54)	1.63 (1.38-1.92)

	High enrolment sites (>=36%)					Low enrolment sites (<=21%)				
	N eligible mothers	N enrolled in FNP	% enrolled in FNP	Crude odds ratio (95% CI)	Adjusted[a] odds ratio (95% CI)	N eligible mothers	N enrolled in FNP	% enrolled in FNP	Crude odds ratio (95% CI)	Adjusted[a] odds ratio (95% CI)
Not linked to NPD	1555 (16.4)	440	28.3	0.48 (0.42-0.54)	-[c]	8190 (14.6)	765	9.3	0.48 (0.44-0.52)	-[c]
**Ever recorded as having Special Educational Needs before 20 weeks pregnancy**										
No	3615 (38.1)	1425	39.4	1 (ref)	1 (ref)	24000 (42.8)	3570	14.9	1 (ref)	1 (ref)
Yes	4245 (44.7)	2175	51.2	1.60 (1.46-1.75)	1.25 (1.13-1.39)	23405 (41.8)	5230	22.3	1.65 (1.57-1.73)	1.22 (1.15-1.28)
Not linked to NPD	1555 (16.4)	440	28.3	0.56 (0.49-0.65)	-[c]	8190 (14.6)	765	9.3	0.56 (0.52-0.61)	-[c]
Linked to NPD but not to NPD census	85 (0.9)	20	23.5	0.50 (0.30-0.82)	-[c]	420 (0.7)	40	9.5	0.60 (0.43-0.82)	-[c]
**Ever recorded as receiving free school meals before 20 weeks of pregnancy**										
No	3370 (35.5)	1330	39.5	1 (ref)	1 (ref)	22330 (39.9)	3055	13.7	1 (ref)	1 (ref)
Yes	4490 (47.3)	2270	50.6	1.54 (1.40-1.69)	1.15 (1.04-1.28)	25080 (44.8)	5740	22.9	1.84 (1.75-1.93)	1.24 (1.17-1.31)
Not linked to NPD	1555 (16.4)	440	28.3	0.56 (0.49-0.65)	-[c]	8190 (14.6)	765	9.3	0.61 (0.56-0.67)	-[c]
Linked to NPD but not to NPD census	85 (0.9)	20	23.5	0.49 (0.30-0.81)	-[c]	420 (0.7)	40	9.5	0.66 (0.48-0.91)	-[c]
**Ever in IDACI bottom decile before 20 weeks of pregnancy**										
No	5540 (58.3)	2445	44.1	1 (ref)	1 (ref)	31310 (55.9)	5235	16.7	1 (ref)	1 (ref)
Yes	2320 (24.4)	1160	50	1.22 (1.10-1.36)	0.99 (0.88-1.11)	16100 (28.7)	3565	22.1	1.38 (1.31-1.45)	1.05 (1.00-1.12)
Not linked to NPD	1555 (16.4)	440	28.3	0.47 (0.41-0.54)	-[c]	8190 (14.6)	765	9.3	0.49 (0.45-0.53)	-[c]
Linked to NPD but not to NPD census	85 (0.9)	20	23.5	0.41 (0.25-0.67)	-[c]	420 (0.7)	40	9.5	0.52 (0.38-0.72)	-[c]
**Educational attainment before 20 weeks of pregnancy**										
Attempted but did not achieve 5 A*-C GCSEs	5830 (61.4)	2660	45.6	1 (ref)	1 (ref)	35715 (63.8)	6130	17.2	1 (ref)	1 (ref)
5 A*-C GCSEs	1615 (17.0)	595	36.8	0.69 (0.61-0.77)	0.92 (0.81-1.04)	8470 (15.1)	1150	13.6	0.76 (0.71-0.81)	1.06 (0.98-1.14)
Not linked to NPD	1555 (16.4)	440	28.3	0.44 (0.39-0.50)	-[c]	8190 (14.6)	765	9.3	0.47 (0.44-0.51)	-[c]
Had not attempted GCSEs prior to 20 weeks pregnancy	500 (5.3)	370	74	3.53 (2.86-4.35)	1.47 (1.13-1.90)	3645 (6.5)	1560	42.8	3.62 (3.37-3.89)	1.58 (1.44-1.73)
**Ever excluded, in pupil referral unit, or alternative provision**										
No	5460 (57.5)	2320	42.5	1 (ref)	1 (ref)	33555 (59.9)	5470	16.3	1 (ref)	1 (ref)
Yes	2480 (26.1)	1300	52.4	1.50 (1.36-1.65)	1.04 (0.93-1.16)	14275 (25.5)	3370	23.6	1.58 (1.51-1.66)	1.03 (0.97-1.08)
Not linked to NPD	1555 (16.4)	440	28.3	0.50 (0.44-0.57)	-[c]	8190 (14.6)	765	9.3	0.50 (0.46-0.55)	-[c]
**Ever persistently absent in a term (>=10% possible sessions)**										
No	5140 (54.1)	1925	37.5	1 (ref)	1 (ref)	29320 (52.3)	3590	12.2	1 (ref)	1 (ref)
Yes	2805 (29.5)	1700	60.6	2.64 (2.40-2.90)	1.47 (1.31-1.66)	18510 (33.0)	5250	28.4	2.87 (2.73-3.01)	1.44 (1.36-1.53)
Not linked to NPD	1555 (16.4)	440	28.3	0.62 (0.54-0.70)	-[c]	8190 (14.6)	765	9.3	0.70 (0.65-0.76)	-[c]

[a]Adjusted models included all variables in the table as covariates

[b]Includes only mothers aged 19 at last menstrual period

[c]Estimates omitted due to multicollinearity

Note: numbers have been rounded to the nearest 5 in accordance with NHS Digital's statistical disclosure rules for sub-national analyses. NPD – National Pupil Database

Table S6. Predictors of enrolment in the Family Nurse Partnership by English region, among mothers aged 13-19 at last menstrual period, living in a Local Authority with an active FNP site at the time of first antenatal appointment – England, births between April 2010-March 2017

	North-East					North-West					Yorkshire and the Humber				
	N eligible mothers	N enrolled in FNP	% enrolled in FNP	Crude odds ratio (95% CI)	Adjusted[a] odds ratio (95% CI)	N eligible mothers	N enrolled in FNP	% enrolled in FNP	Crude odds ratio (95% CI)	Adjusted[a] odds ratio (95% CI)	N eligible mothers	N enrolled in FNP	% enrolled in FNP	Crude odds ratio (95% CI)	Adjusted[a] odds ratio (95% CI)
**Total**	9,260	1,940	21	-	-	14,970	3,895	26	-	-	19,530	3,440	17.6	-	-
**Maternal age at birth**															
13-15	240 (2.6)	130	54.2	5.42 (4.15-7.06)	3.03 (2.16-4.24)	295 (2.0)	160	54.2	4.13 (3.24-5.26)	2.78 (2.07-3.74)	475 (2.4)	190	40	3.83 (3.15-4.65)	2.29 (1.78-2.93)
16-17	2065 (22.3)	710	34.4	2.48 (2.21-2.79)	1.91 (1.65-2.20)	3050 (20.4)	1300	42.6	2.48 (2.26-2.71)	1.83 (1.64-2.04)	4110 (21.0)	1305	31.8	2.60 (2.39-2.84)	1.88 (1.69-2.09)
18-19	5070 (54.8)	895	17.7	1 (ref)	1 (ref)	8365 (55.9)	2015	24.1	1 (ref)	1 (ref)	10695 (54.8)	1635	15.3	1 (ref)	1 (ref)
20[b]	1890 (20.4)	205	10.8	0.56 (0.47-0.65)	0.62 (0.53-0.74)	3260 (21.8)	415	12.7	0.45 (0.40-0.50)	0.50 (0.44-0.56)	4250 (21.8)	310	7.3	0.42 (0.37-0.48)	0.52 (0.46-0.60)
**Ethnicity**															
White	8950 (96.7)	1905	21.3	1 (ref)	1 (ref)	13480 (90.0)	3635	27	1 (ref)	1 (ref)	17280 (88.5)	3180	18.4	1 (ref)	1 (ref)
South Asian	55 (0.6)	-	-	0.45 (0.20-1.01)	0.58 (0.24-1.37)	315 (2.1)	45	14.3	0.40 (0.29-0.56)	0.61 (0.44-0.86)	650 (3.3)	55	8.5	0.42 (0.32-0.55)	0.60 (0.45-0.80)
Black	20 (0.2)	-	-	0.68 (0.19-2.37)	0.89 (0.24-3.36)	240 (1.6)	50	20.8	0.81 (0.59-1.12)	1.05 (0.75-1.48)	270 (1.4)	40	14.8	0.87 (0.62-1.23)	1.11 (0.77-1.60)
Mixed/other	110 (1.2)	10	9.1	0.44 (0.24-0.80)	0.50 (0.26-0.93)	545 (3.6)	120	22	0.81 (0.65-1.00)	0.97 (0.77-1.23)	860 (4.4)	110	12.8	0.64 (0.52-0.79)	0.69 (0.55-0.85)
Unknown	125 (1.3)	10	8	0.33 (0.17-0.63)	0.37 (0.19-0.73)	390 (2.6)	45	11.5	0.40 (0.29-0.54)	0.50 (0.36-0.70)	465 (2.4)	60	12.9	0.59 (0.44-0.78)	0.71 (0.53-0.95)
**Index of Multiple Deprivation (quintile)**															
Least deprived	240 (2.6)	40	16.7	0.89 (0.60-1.32)	0.90 (0.60-1.36)	385 (2.6)	100	26	0.87 (0.66-1.14)	0.95 (0.72-1.27)	410 (2.1)	55	13.4	0.86 (0.63-1.19)	0.93 (0.66-1.29)
2	465 (5.0)	90	19.4	1.14 (0.85-1.53)	1.15 (0.85-1.57)	670 (4.5)	165	24.6	0.91 (0.73-1.14)	0.92 (0.73-1.17)	1270 (6.5)	175	13.8	0.88 (0.71-1.08)	0.89 (0.72-1.11)
3	835 (9.0)	140	16.8	1 (ref)	1 (ref)	1295 (8.7)	320	24.7	1 (ref)	1 (ref)	1940 (9.9)	315	16.2	1 (ref)	1 (ref)
4	2295 (24.8)	490	21.4	1.33 (1.08-1.63)	1.23 (0.99-1.53)	2635 (17.6)	665	25.2	0.99 (0.85-1.17)	0.94 (0.80-1.12)	4340 (22.2)	765	17.6	1.11 (0.96-1.28)	1.07 (0.92-1.25)
Most deprived	5420 (58.5)	1175	21.7	1.35 (1.11-1.63)	1.21 (0.99-1.48)	9960 (66.5)	2615	26.3	1.12 (0.97-1.29)	0.97 (0.83-1.14)	11570 (59.2)	2130	18.4	1.26 (1.10-1.44)	1.08 (0.94-1.25)
Unknown	-	-	-	-	-	25 (0.2)	25	100	-	-	-	-	-	-	-
**Admission with diagnoses within 2 years before 20 weeks gestation**															
Mental health (excluding substance misuse and self-harm)	180 (1.9)	60	33.3	1.93 (1.41-2.64)	1.41 (0.96-2.06)	430 (2.9)	200	46.5	2.35 (1.93-2.87)	1.56 (1.21-2.00)	305 (1.6)	95	31.1	2.13 (1.66-2.74)	1.57 (1.16-2.13)
Adversity-related	445 (4.8)	145	32.6	1.96 (1.60-2.41)	1.15 (0.84-1.57)	795 (5.3)	365	45.9	2.56 (2.21-2.98)	1.51 (1.22-1.88)	725 (3.7)	225	31	2.17 (1.84-2.56)	1.09 (0.86-1.40)
Any chronic condition	880 (9.5)	245	27.8	1.52 (1.30-1.78)	1.09 (0.85-1.39)	1665 (11.1)	615	36.9	1.73 (1.55-1.93)	1.01 (0.86-1.20)	1545 (7.9)	395	25.6	1.67 (1.48-1.89)	1.17 (0.98-1.41)
A&E visits	6115 (66.0)	1390	22.7	1.35 (1.21-1.51)	1.24 (1.10-1.40)	10170 (67.9)	2910	28.6	1.52 (1.40-1.65)	1.30 (1.18-1.43)	12090 (61.9)	2375	19.6	1.44 (1.33-1.56)	1.26 (1.16-1.38)
**Gestational age at antenatal booking appointment**															
Before 10 weeks	3170 (34.2)	685	21.6	1 (ref)	1 (ref)	4245 (28.4)	1155	27.2	1 (ref)	1 (ref)	4070 (20.8)	785	19.3	1 (ref)	1 (ref)
10-20 weeks	4140 (44.7)	830	20	0.92 (0.82-1.04)	0.83 (0.74-0.94)	5975 (39.9)	1560	26.1	0.92 (0.84-1.01)	0.89 (0.81-0.99)	5555 (28.4)	865	15.6	0.75 (0.67-0.84)	0.74 (0.65-0.83)
20 weeks or more	490 (5.3)	80	16.3	0.74 (0.57-0.95)	0.51 (0.39-0.67)	740 (4.9)	200	27	0.91 (0.76-1.09)	0.72 (0.59-0.88)	785 (4.0)	195	24.8	1.21 (1.00-1.46)	0.98 (0.80-1.20)
Unknown	1465 (15.8)	345	23.5	1.28 (1.09-1.50)	1.08 (0.91-1.28)	4010 (26.8)	985	24.6	0.80 (0.72-0.89)	0.69 (0.61-0.77)	9120 (46.7)	1595	17.5	1.01 (0.90-1.13)	0.87 (0.77-0.99)
**Linked to NPD**															
Linked to NPD	8195 (88.5)	1780	21.7	1 (ref)	1 (ref)	12400 (82.8)	3475	28	1 (ref)	1 (ref)	16760 (85.8)	3195	19.1	1 (ref)	1 (ref)
Not linked to NPD	1030 (11.1)	155	15	0.66 (0.55-0.78)	1.15 (0.91-1.44)	2480 (16.6)	410	16.5	0.50 (0.45-0.56)	0.91 (0.78-1.07)	2645 (13.5)	240	9.1	0.43 (0.38-0.50)	0.86 (0.72-1.02)
Linked to NPD but not to NPD census	35 (0.4)	-	-	0.48 (0.17-1.35)	1.22 (0.42-3.53)	90 (0.6)	10	11.1	0.28 (0.14-0.57)	0.41 (0.20-0.85)	120 (0.6)	-	-	0.27 (0.13-0.58)	0.45 (0.21-0.99)
**Ever had a child protection plan or was looked after before 20 weeks pregnancy**															

	North-East					North-West					Yorkshire and the Humber				
	N eligible mothers	N enrolled in FNP	% enrolled in FNP	Crude odds ratio (95% CI)	Adjusted[a] odds ratio (95% CI)	N eligible mothers	N enrolled in FNP	% enrolled in FNP	Crude odds ratio (95% CI)	Adjusted[a] odds ratio (95% CI)	N eligible mothers	N enrolled in FNP	% enrolled in FNP	Crude odds ratio (95% CI)	Adjusted[a] odds ratio (95% CI)
No CPP or looked after	7590 (82.0)	1530	20.2	1 (ref)	1 (ref)	11440 (76.4)	2955	25.8	1 (ref)	1 (ref)	15830 (81.1)	2785	17.6	1 (ref)	1 (ref)
Looked after (CPP)	475 (5.1)	185	38.9	2.58 (2.12-3.13)	2.02 (1.63-2.49)	795 (5.3)	400	50.3	2.92 (2.51-3.40)	2.20 (1.86-2.59)	765 (3.9)	295	38.6	3.03 (2.60-3.54)	2.31 (1.96-2.73)
Child protection plan, but not looked after	170 (1.8)	65	38.2	2.37 (1.73-3.26)	1.37 (0.98-1.93)	255 (1.7)	125	49	2.58 (2.00-3.34)	1.52 (1.15-1.99)	290 (1.5)	125	43.1	3.49 (2.74-4.44)	1.95 (1.51-2.51)
Not linked to NPD	1030 (11.1)	155	15	0.72 (0.60-0.86)	-[c]	2480 (16.6)	410	16.5	0.56 (0.50-0.63)	-[c]	2645 (13.5)	240	9.1	0.48 (0.42-0.55)	-[c]
**Ever recorded as having Special Educational Needs before 20 weeks pregnancy**															
No	4345 (46.9)	795	18.3	1 (ref)	1 (ref)	6235 (41.6)	1435	23	1 (ref)	1 (ref)	8630 (44.2)	1290	14.9	1 (ref)	1 (ref)
Yes	3850 (41.6)	980	25.5	1.51 (1.36-1.68)	1.16 (1.02-1.31)	6160 (41.1)	2040	33.1	1.60 (1.48-1.74)	1.23 (1.12-1.36)	8130 (41.6)	1905	23.4	1.75 (1.62-1.90)	1.23 (1.12-1.34)
Not linked to NPD	1030 (11.1)	155	15	0.81 (0.67-0.97)	-[c]	2480 (16.6)	410	16.5	0.65 (0.57-0.73)	-[c]	2645 (13.5)	240	9.1	0.58 (0.50-0.68)	-[c]
Linked to NPD but not to NPD census	35 (0.4)	-	-	0.58 (0.20-1.65)	-[c]	90 (0.6)	10	11.1	0.36 (0.18-0.73)	-[c]	120 (0.6)	-	-	0.36 (0.17-0.78)	-[c]
**Ever recorded as receiving free school meals before 20 weeks of pregnancy**															
No	3340 (36.1)	530	15.9	1 (ref)	1 (ref)	4660 (31.1)	1015	21.8	1 (ref)	1 (ref)	7855 (40.2)	1150	14.6	1 (ref)	1 (ref)
Yes	4855 (52.4)	1250	25.7	1.79 (1.60-2.01)	1.24 (1.09-1.41)	7740 (51.7)	2455	31.7	1.62 (1.48-1.76)	1.18 (1.07-1.31)	8910 (45.6)	2040	22.9	1.74 (1.60-1.88)	1.15 (1.05-1.26)
Not linked to NPD	1030 (11.1)	155	15	0.95 (0.78-1.15)	-[c]	2480 (16.6)	410	16.5	0.70 (0.61-0.79)	-[c]	2645 (13.5)	240	9.1	0.59 (0.51-0.69)	-[c]
Linked to NPD but not to NPD census	35 (0.4)	-	-	0.68 (0.24-1.94)	-[c]	90 (0.6)	10	11.1	0.39 (0.19-0.78)	-[c]	120 (0.6)	-	-	0.37 (0.17-0.79)	-[c]
**Ever in IDACI bottom decile before 20 weeks of pregnancy**															
No	4655 (50.3)	895	19.2	1 (ref)	1 (ref)	6170 (41.2)	1585	25.7	1 (ref)	1 (ref)	10525 (53.9)	1810	17.2	1 (ref)	1 (ref)
Yes	3540 (38.2)	885	25	1.38 (1.23-1.53)	1.10 (0.97-1.24)	6230 (41.6)	1885	30.3	1.30 (1.19-1.41)	1.02 (0.92-1.12)	6235 (31.9)	1380	22.1	1.44 (1.33-1.56)	1.11 (1.02-1.22)
Not linked to NPD	1030 (11.1)	155	15	0.76 (0.63-0.92)	-[c]	2480 (16.6)	410	16.5	0.58 (0.51-0.66)	-[c]	2645 (13.5)	240	9.1	0.50 (0.43-0.58)	-[c]
Linked to NPD but not to NPD census	35 (0.4)	-	-	0.55 (0.19-1.56)	-[c]	90 (0.6)	10	11.1	0.33 (0.16-0.66)	-[c]	120 (0.6)	-	-	0.31 (0.14-0.67)	-[c]
**Educational attainment before 20 weeks of pregnancy**															
Attempted but did not achieve 5 A*-C GCSEs	5905 (63.8)	1155	19.6	1 (ref)	1 (ref)	9290 (62.1)	2475	26.6	1 (ref)	1 (ref)	12640 (64.7)	2285	18.1	1 (ref)	1 (ref)
5 A*-C GCSEs	1660 (17.9)	290	17.5	0.88 (0.76-1.01)	1.16 (1.00-1.36)	2345 (15.7)	545	23.2	0.75 (0.67-0.84)	1.04 (0.92-1,18)	2935 (15.0)	380	12.9	0.65 (0.58-0.74)	0.95 (0.83-1.08)
Not linked to NPD	1030 (11.1)	155	15	0.75 (0.62-0.90)	-[c]	2480 (16.6)	410	16.5	0.53 (0.47-0.60)	-[c]	2645 (13.5)	240	9.1	0.46 (0.40-0.53)	-[c]
Had not attempted GCSEs prior to 20 weeks pregnancy	665 (7.2)	335	50.4	4.13 (3.50-4.87)	1.93 (1.56-2.39)	855 (5.7)	465	54.4	3.47 (2.99-4.02)	1.50 (1.25-1.80)	1310 (6.7)	530	40.5	3.13 (2.77-3.54)	1.33 (1.14-1.56)
**Ever excluded, in pupil referral unit, or alternative provision**															
No	6260 (67.6)	1240	19.8	1 (ref)	1 (ref)	8710 (58.2)	2210	25.4	1 (ref)	1 (ref)	12025 (61.6)	2005	16.7	1 (ref)	1 (ref)
Yes	1975 (21.3)	540	27.3	1.56 (1.38-1.75)	1.09 (0.95-1.24)	3780 (25.3)	1270	33.6	1.58 (1.45-1.72)	1.02 (0.92-1.12)	4860 (24.9)	1195	24.6	1.67 (1.54-1.81)	1.10 (1.00-1.21)
Not linked to NPD	1030 (11.1)	155	15	0.74 (0.62-0.89)	-[c]	2480 (16.6)	410	16.5	0.58 (0.52-0.66)	-[c]	2645 (13.5)	240	9.1	0.51 (0.45-0.59)	-[c]
**Ever persistently absent in a term (>=10% possible sessions)**															
No	5035 (54.4)	805	16	1 (ref)	1 (ref)	7790 (52.0)	1610	20.7	1 (ref)	1 (ref)	10270 (52.6)	1295	12.6	1 (ref)	1 (ref)
Yes	3200 (34.6)	980	30.6	2.35 (2.11-2.62)	1.19 (1.03-1.36)	4700 (31.4)	1875	39.9	2.72 (2.51-2.96)	1.41 (1.27-1.56)	6615 (33.9)	1905	28.8	2.88 (2.66-3.12)	1.46 (1.32-1.62)
Not linked to NPD	1030 (11.1)	155	15	0.97 (0.80-1.16)	-[c]	2480 (16.6)	410	16.5	0.77 (0.69-0.88)	-[c]	2645 (13.5)	240	9.1	0.72 (0.62-0.83)	-[c]

Table S6. Predictors of enrolment in the Family Nurse Partnership by English region, among mothers aged 13-19 at last menstrual period, living in a Local Authority with an active FNP site at the time of first antenatal appointment – England, births between April 2010-March 2017 (continued)

	East Midlands					West Midlands					East of England				
	N eligible mothers	N enrolled in FNP	% enrolled in FNP	Crude odds ratio (95% CI)	Adjusted[a] odds ratio (95% CI)	N eligible mothers	N enrolled in FNP	% enrolled in FNP	Crude odds ratio (95% CI)	Adjusted[a] odds ratio (95% CI)	N eligible mothers	N enrolled in FNP	% enrolled in FNP	Crude odds ratio (95% CI)	Adjusted[a] odds ratio (95% CI)
**Total**	10,545	2,160	20.5	-	-	15,250	3,435	22.5	-	-	9,890	2,085	21.1	-	-
**Maternal age at birth**															
13-15	255 (2.4)	145	56.9	6.57 (5.05-8.55)	3.48 (2.50-4.85)	330 (2.2)	160	48.5	3.99 (3.18-5.01)	2.13 (1.61-2.83)	200 (2.0)	95	47.5	4.09 (3.06-5.48)	2.21 (1.54-3.16)
16-17	2235 (21.2)	865	38.7	3.06 (2.74-3.42)	2.08 (1.81-2.39)	3275 (21.5)	1160	35.4	2.21 (2.02-2.42)	1.59 (1.42-1.78)	2090 (21.1)	740	35.4	2.44 (2.18-2.74)	1.71 (1.49-1.97)
18-19	5710 (54.1)	995	17.4	1 (ref)	1 (ref)	8270 (54.2)	1735	21	1 (ref)	1 (ref)	5505 (55.7)	1030	18.7	1 (ref)	1 (ref)
20[b]	2340 (22.2)	155	6.6	0.33 (0.28-0.40)	0.41 (0.34-0.50)	3375 (22.1)	380	11.3	0.47 (0.42-0.54)	0.56 (0.49-0.63)	2095 (21.2)	220	10.5	0.52 (0.45-0.61)	0.62 (0.52-0.73)
**Ethnicity**															
White	9215 (87.4)	1895	20.6	1 (ref)	1 (ref)	12680 (83.1)	3005	23.7	1 (ref)	1 (ref)	9080 (91.8)	1920	21.1	1 (ref)	1 (ref)
South Asian	205 (1.9)	35	17.1	0.68 (0.47-0.98)	0.94 (0.63-1.42)	575 (3.8)	70	12.2	0.47 (0.36-0.60)	0.63 (0.48-0.83)	110 (1.1)	15	13.6	0.54 (0.31-0.94)	0.78 (0.44-1.39)
Black	165 (1.6)	25	15.2	0.67 (0.44-1.02)	0.91 (0.57-1.44)	445 (2.9)	105	23.6	1.02 (0.81-1.28)	1.21 (0.95-1.54)	90 (0.9)	25	27.8	1.11 (0.68-1.81)	1.33 (0.80-2.23)
Mixed/other	520 (4.9)	120	23.1	0.89 (0.71-1.10)	0.91 (0.72-1.16)	1020 (6.7)	170	16.7	0.67 (0.57-0.80)	0.75 (0.63-0.90)	250 (2.5)	60	24	1.05 (0.77-1.41)	1.18 (0.86-1.63)
Unknown	440 (4.2)	80	18.2	0.76 (0.59-0.98)	0.94 (0.71-1.24)	530 (3.5)	85	16	0.57 (0.45-0.73)	0.70 (0.55-0.91)	360 (3.6)	65	18.1	0.68 (0.52-0.90)	0.77 (0.58-1.04)
**Index of Multiple Deprivation (quintile)**															
Least deprived	675 (6.4)	105	15.6	0.95 (0.73-1.22)	1.15 (0.88-1.52)	525 (3.4)	125	23.8	0.96 (0.76-1.22)	1.06 (0.83-1.36)	1045 (10.6)	150	14.4	0.67 (0.55-0.83)	0.69 (0.56-0.86)
2	830 (7.9)	145	17.5	1.04 (0.83-1.31)	1.20 (0.94-1.54)	920 (6.0)	230	25	1.06 (0.87-1.28)	1.08 (0.88-1.32)	1530 (15.5)	270	17.6	0.85 (0.72-1.01)	0.85 (0.72-1.02)
3	1455 (13.8)	260	17.9	1 (ref)	1 (ref)	1785 (11.7)	395	22.1	1 (ref)	1 (ref)	2330 (23.6)	470	20.2	1 (ref)	1 (ref)
4	2825 (26.8)	565	20	1.10 (0.93-1.30)	1.07 (0.89-1.27)	3220 (21.1)	730	22.7	1.11 (0.96-1.28)	1.07 (0.92-1.24)	2660 (26.9)	560	21.1	0.99 (0.86-1.14)	0.99 (0.85-1.14)
Most deprived	4745 (45.0)	1075	22.7	1.20 (1.02-1.40)	1.05 (0.88-1.25)	8785 (57.6)	1940	22.1	1.21 (1.06-1.39)	1.11 (0.96-1.27)	2325 (23.5)	635	27.3	1.35 (1.17-1.56)	1.35 (1.16-1.58)
Unknown	10 (0.1)	10	100	-	-	15 (0.1)	15	100	-	-	-	-	-	-	-
**Admission with diagnoses within 2 years before 20 weeks gestation**															
Mental health (excluding substance misuse and self-harm)	255 (2.4)	80	31.4	1.89 (1.43-2.48)	1.15 (0.81-1.64)	310 (2.0)	125	40.3	2.30 (1.82-2.91)	1.44 (1.07-1.94)	250 (2.5)	90	36	2.30 (1.76-3.00)	1.36 (0.96-1.94)
Adversity-related	470 (4.5)	175	37.2	2.49 (2.04-3.03)	1.61 (1.18-2.20)	550 (3.6)	205	37.3	2.09 (1.74-2.51)	0.97 (0.75-1.25)	365 (3.7)	140	38.4	2.62 (2.10-3.27)	1.30 (0.95-1.80)
Any chronic condition	945 (9.0)	270	28.6	1.68 (1.44-1.96)	0.99 (0.77-1.28)	1250 (8.2)	420	33.6	1.77 (1.56-2.02)	1.32 (1.10-1.58)	820 (8.3)	250	30.5	1.80 (1.53-2.11)	1.17 (0.92-1.49)
A&E visits	6200 (58.8)	1420	22.9	1.45 (1.31-1.61)	1.31 (1.17-1.47)	9325 (61.1)	2330	25	1.40 (1.29-1.52)	1.25 (1.14-1.36)	5785 (58.5)	1360	23.5	1.47 (1.33-1.63)	1.26 (1.12-1.40)
**Gestational age at antenatal booking appointment**															
Before 10 weeks	3485 (33.0)	650	18.7	1 (ref)	1 (ref)	3515 (23.0)	750	21.3	1 (ref)	1 (ref)	3400 (34.4)	695	20.4	1 (ref)	1 (ref)
10-20 weeks	3870 (36.7)	800	20.7	0.99 (0.88-1.11)	0.96 (0.84-1.09)	4545 (29.8)	1065	23.4	1.01 (0.90-1.13)	1.00 (0.89-1.12)	3420 (34.6)	655	19.2	0.93 (0.82-1.05)	0.91 (0.80-1.03)
20 weeks or more	790 (7.5)	200	25.3	1.05 (0.86-1.28)	0.83 (0.67-1.02)	945 (6.2)	265	28	1.02 (0.85-1.23)	0.84 (0.69-1.03)	435 (4.4)	85	19.5	0.92 (0.72-1.19)	0.83 (0.64-1.10)
Unknown	2400 (22.8)	510	21.3	0.95 (0.82-1.09)	0.86 (0.74-1.00)	6250 (41.0)	1355	21.7	0.75 (0.66-0.85)	0.73 (0.64-0.83)	2635 (26.6)	650	24.7	1.13 (0.99-1.28)	1.03 (0.90-1.18)
**Linked to NPD**															
Linked to NPD	9070 (86.0)	1995	22	1 (ref)	1 (ref)	13130 (86.1)	3190	24.3	1 (ref)	1 (ref)	8420 (85.1)	1920	22.8	1 (ref)	1 (ref)
Not linked to NPD	1400 (13.3)	155	11.1	0.41 (0.34-0.49)	0.90 (0.72-1.12)	1990 (13.0)	225	11.3	0.41 (0.35-0.47)	0.74 (0.62-0.89)	1410 (14.3)	160	11.3	0.42 (0.35-0.50)	0.79 (0.64-0.98)
Linked to NPD but not to NPD census	75 (0.7)	10	13.3	0.39 (0.19-0.83)	0.60 (0.26-1.38)	130 (0.9)	15	11.5	0.52 (0.31-0.87)	0.81 (0.47-1.38)	55 (0.6)	-	-	0.56 (0.25-1.26)	0.93 (0.41-2.13)
**Ever had a child protection plan or was looked after before 20 weeks pregnancy**															
No CPP or looked after	8460 (80.2)	1710	20.2	1 (ref)	1 (ref)	12290 (80.6)	2805	22.8	1 (ref)	1 (ref)	7860 (79.5)	1670	21.2	1 (ref)	1 (ref)

	East Midlands					West Midlands					East of England				
	**N eligible mothers**	**N enrolled in FNP**	**% enrolled in FNP**	**Crude odds ratio (95% CI)**	**Adjusted[a] odds ratio (95% CI)**	**N eligible mothers**	**N enrolled in FNP**	**% enrolled in FNP**	**Crude odds ratio (95% CI)**	**Adjusted[a] odds ratio (95% CI)**	**N eligible mothers**	**N enrolled in FNP**	**% enrolled in FNP**	**Crude odds ratio (95% CI)**	**Adjusted[a] odds ratio (95% CI)**
Looked after (CPP)	500 (4.7)	190	38	2.47 (2.04-3.00)	1.69 (1.37-2.10)	745 (4.9)	315	42.3	2.43 (2.08-2.84)	1.89 (1.60-2.23)	485 (4.9)	200	41.2	2.66 (2.19-3.22)	1.92 (1.56-2.36)
Child protection plan, but not looked after	190 (1.8)	105	55.3	5.27 (3.91-7.11)	2.60 (1.87-3.61)	225 (1.5)	90	40	2.16 (1.63-2.84)	1.24 (0.92-1.66)	130 (1.3)	55	42.3	2.46 (1.71-3.52)	1.25 (0.85-1.84)
Not linked to NPD	1400 (13.3)	155	11.1	0.46 (0.38-0.54)	-[c]	1990 (13.0)	225	11.3	0.44 (0.38-0.51)	-[c]	1410 (14.3)	160	11.3	0.46 (0.39-0.55)	-[c]
**Ever recorded as having Special Educational Needs before 20 weeks pregnancy**															
No	4210 (39.9)	695	16.5	1 (ref)	1 (ref)	6920 (45.4)	1515	21.9	1 (ref)	1 (ref)	4175 (42.2)	770	18.4	1 (ref)	1 (ref)
Yes	4860 (46.1)	1305	26.9	1.88 (1.69-2.08)	1.34 (1.19-1.51)	6210 (40.7)	1675	27	1.31 (1.21-1.42)	1.05 (0.96-1.15)	4245 (42.9)	1150	27.1	1.74 (1.56-1.93)	1.34 (1.19-1.50)
Not linked to NPD	1400 (13.3)	155	11.1	0.59 (0.49-0.71)	-[c]	1990 (13.0)	225	11.3	0.47 (0.40-0.54)	-[c]	1410 (14.3)	160	11.3	0.57 (0.47-0.68)	-[c]
Linked to NPD but not to NPD census	75 (0.7)	10	13.3	0.57 (0.27-1.20)	-[c]	130 (0.9)	15	11.5	0.60 (0.36-1.00)	-[c]	55 (0.6)	-	-	0.76 (0.34-1.70)	-[c]
**Ever recorded as receiving free school meals before 20 weeks of pregnancy**															
No	4295 (40.7)	645	15	1 (ref)	1 (ref)	5750 (37.7)	1130	19.7	1 (ref)	1 (ref)	4245 (42.9)	760	17.9	1 (ref)	1 (ref)
Yes	4775 (45.3)	1350	28.3	2.06 (1.85-2.29)	1.40 (1.24-1.58)	7380 (48.4)	2060	27.9	1.61 (1.48-1.75)	1.22 (1.11-1.34)	4175 (42.2)	1155	27.7	1.70 (1.53-1.89)	1.18 (1.05-1.33)
Not linked to NPD	1400 (13.3)	155	11.1	0.63 (0.52-0.76)	-[c]	1990 (13.0)	225	11.3	0.54 (0.47-0.64)	-[c]	1410 (14.3)	160	11.3	0.56 (0.46-0.67)	-[c]
Linked to NPD but not to NPD census	75 (0.7)	10	13.3	0.61 (0.29-1.28)	-[c]	130 (0.9)	15	11.5	0.71 (0.42-1.19)	-[c]	55 (0.6)	-	-	0.75 (0.33-1.66)	-[c]
**Ever in IDACI bottom decile before 20 weeks of pregnancy**															
No	6135 (58.2)	1170	19.1	1 (ref)	1 (ref)	8465 (55.5)	2000	23.6	1 (ref)	1 (ref)	6830 (69.1)	1465	21.4	1 (ref)	1 (ref)
Yes	2935 (27.8)	830	28.3	1.40 (1.25-1.56)	1.04 (0.91-1.18)	4665 (30.6)	1190	25.5	1.26 (1.15-1.37)	1.04 (0.94-1.15)	1590 (16.1)	450	28.3	1.36 (1.20-1.55)	0.98 (0.85-1.14)
Not linked to NPD	1400 (13.3)	155	11.1	0.47 (0.39-0.56)	-[c]	1990 (13.0)	225	11.3	0.45 (0.38-0.52)	-[c]	1410 (14.3)	160	11.3	0.45 (0.37-0.53)	-[c]
Linked to NPD but not to NPD census	75 (0.7)	10	13.3	0.46 (0.22-0.96)	-[c]	130 (0.9)	15	11.5	0.58 (0.35-0.97)	-[c]	55 (0.6)	-	-	0.60 (0.27-1.34)	-[c]
**Educational attainment before 20 weeks of pregnancy**															
Attempted but did not achieve 5 A*-C GCSEs	6805 (64.5)	1405	20.6	0.64 (0.55-0.75)	1 (ref)	9740 (63.9)	2190	22.5	1 (ref)	1 (ref)	6370 (64.4)	1375	21.6	1 (ref)	1 (ref)
5 A*-C GCSEs	1615 (15.3)	230	14.2	0.45 (0.37-0.53)	0.94 (0.79-1.11)	2500 (16.4)	530	21.2	0.89 (0.80-1.00)	1.13 (1.00-1.27)	1495 (15.1)	265	17.7	0.76 (0.66-0.89)	1.11 (0.94-1.30)
Not linked to NPD	1400 (13.3)	155	11.1	4.17 (3.55-4.90)	-[c]	1990 (13.0)	225	11.3	0.45 (0.39-0.53)	-[c]	1410 (14.3)	160	11.3	0.45 (0.38-0.54)	-[c]
Had not attempted GCSEs prior to 20 weeks pregnancy	720 (6.8)	370	51.4	0.64 (0.55-0.75)	1.41 (1.15-1.73)	1020 (6.7)	490	48	3.46 (3.03-3.96)	1.76 (1.48-2.08)	615 (6.2)	285	46.3	3.26 (2.75-3.88)	1.55 (1.24-1.92)
**Ever excluded, in pupil referral unit, or alternative provision**															
No	6390 (60.6)	1270	19.9	1 (ref)	1 (ref)	9275 (60.8)	2030	21.9	1 (ref)	1 (ref)	5930 (60.0)	1205	20.3	1 (ref)	1 (ref)
Yes	2755 (26.1)	735	26.7	1.49 (1.34-1.66)	0.90 (0.80-1.02)	3985 (26.1)	1175	29.5	1.46 (1.34-1.59)	1.06 (0.96-1.16)	2550 (25.8)	720	28.2	1.58 (1.42-1.76)	1.05 (0.93-1.18)
Not linked to NPD	1400 (13.3)	155	11.1	0.47 (0.39-0.56)	-[c]	1990 (13.0)	225	11.3	0.46 (0.40-0.54)	-[c]	1410 (14.3)	160	11.3	0.49 (0.41-0.58)	-[c]
**Ever persistently absent in a term (>=10% possible sessions)**															
No	5545 (52.6)	735	13.3	1 (ref)	1 (ref)	8095 (53.1)	1455	18	1 (ref)	1 (ref)	5145 (52.0)	845	16.4	1 (ref)	1 (ref)
Yes	3600 (34.1)	1270	35.3	3.67 (3.30-4.08)	1.59 (1.40-1.82)	5165 (33.9)	1750	33.9	2.48 (2.28-2.69)	1.40 (1.26-1.55)	3330 (33.7)	1080	32.4	2.51 (2.26-2.79)	1.43 (1.25-1.63)
Not linked to NPD	1400 (13.3)	155	11.1	0.77 (0.63-0.92)	-[c]	1990 (13.0)	225	11.3	0.61 (0.52-0.71)	-[c]	1410 (14.3)	160	11.3	0.63 (0.53-0.76)	-[c]

Table S6. Predictors of enrolment in the Family Nurse Partnership by English region, among mothers aged 13-19 at last menstrual period, living in a Local Authority with an active FNP site at the time of first antenatal appointment – England, births between April 2010-March 2017 (continued)

	London					South-East					South-West				
	**N eligible mothers**	**N enrolled in FNP**	**% enrolled in FNP**	**Crude odds ratio (95% CI)**	**Adjusted[a] odds ratio (95% CI)**	**N eligible mothers**	**N enrolled in FNP**	**% enrolled in FNP**	**Crude odds ratio (95% CI)**	**Adjusted[a] odds ratio (95% CI)**	**N eligible mothers**	**N enrolled in FNP**	**% enrolled in FNP**	**Crude odds ratio (95% CI)**	**Adjusted[a] odds ratio (95% CI)**
**Total**	12,010	3,600	30	-	-	13,625	3,730	27.4	-	-	5,440	1,395	25.6	-	-
**Maternal age at birth**															
13-15	235 (2.0)	135	57.4	4.02 (3.07-5.27)	2.58 (1.86-3.58)	275 (2.0)	170	61.8	5.99 (4.62-7.76)	3.48 (2.55-4.75)	80 (1.5)	50	62.5	7.20 (4.44-11.68)	3.85 (2.17-6.81)
16-17	2205 (18.4)	980	44.4	2.07 (1.87-2.29)	1.60 (1.42-1.81)	2690 (19.7)	1230	45.7	2.77 (2.52-3.05)	1.93 (1.72-2.17)	1000 (18.4)	435	43.5	2.61 (2.24-3.05)	1.76 (1.45-2.14)
18-19	6680 (55.6)	1905	28.5	1 (ref)	1 (ref)	7750 (56.9)	1930	24.9	1 (ref)	1 (ref)	3050 (56.1)	735	24.1	1 (ref)	1 (ref)
20[b]	2890 (24.1)	585	20.2	0.62 (0.56-0.69)	0.68 (0.61-0.76)	2910 (21.4)	395	13.6	0.45 (0.40-0.50)	0.54 (0.47-0.61)	1315 (24.2)	175	13.3	0.45 (0.38-0.55)	0.52 (0.43-0.64)
**Ethnicity**															
White	6025 (50.2)	1690	28	1 (ref)	1 (ref)	11895 (87.3)	3290	27.7	1 (ref)	1 (ref)	5125 (94.2)	1330	26	1 (ref)	1 (ref)
South Asian	1000 (8.3)	265	26.5	0.80 (0.68-0.94)	0.98 (0.83-1.17)	235 (1.7)	35	14.9	0.42 (0.29-0.60)	0.65 (0.44-0.95)	25 (0.5)	-	-	0.54 (0.20-1.47)	1.11 (0.38-3.19)
Black	2525 (21.0)	885	35	1.54 (1.38-1.71)	1.61 (1.44-1.80)	170 (1.2)	55	32.4	1.27 (0.91-1.78)	1.47 (1.02-2.13)	45 (0.8)	-	-	0.45 (0.20-1.02)	0.54 (0.22-1.30)
Mixed/other	1880 (15.7)	605	32.2	1.27 (1.13-1.43)	1.36 (1.20-1.53)	405 (3.0)	110	27.2	0.92 (0.73-1.15)	0.99 (0.77-1.26)	110 (2.0)	25	22.7	0.74 (0.47-1.16)	0.84 (0.51-1.37)
Unknown	580 (4.8)	160	27.6	0.99 (0.81-1.20)	1.43 (1.16-1.76)	920 (6.8)	240	26.1	0.85 (0.72-1.00)	0.96 (0.81-1.14)	140 (2.6)	25	17.9	0.49 (0.32-0.76)	0.60 (0.38-0.97)
**Index of Multiple Deprivation (quintile)**															
Least deprived	105 (0.9)	45	42.9	1.79 (1.18-2.71)	1.68 (1.08-2.63)	1785 (13.1)	410	23	0.73 (0.63-0.84)	0.76 (0.65-0.89)	380 (7.0)	100	26.3	0.67 (0.50-0.89)	0.72 (0.53-0.98)
2	395 (3.3)	135	34.2	1.13 (0.88-1.45)	1.17 (0.90-1.52)	1865 (13.7)	445	23.9	0.81 (0.71-0.93)	0.89 (0.77-1.03)	615 (11.3)	170	27.6	0.88 (0.69-1.11)	0.87 (0.67-1.12)
3	1305 (10.9)	390	29.9	1 (ref)	1 (ref)	2795 (20.5)	770	27.5	1 (ref)	1 (ref)	1100 (20.2)	275	25	1 (ref)	1 (ref)
4	4155 (34.6)	1195	28.8	1.00 (0.87-1.16)	0.95 (0.81-1.10)	3770 (27.7)	1080	28.6	1.09 (0.97-1.22)	1.03 (0.91-1.16)	1625 (29.9)	385	23.7	1.01 (0.84-1.21)	0.92 (0.76-1.12)
Most deprived	5990 (49.9)	1775	29.6	1.03 (0.90-1.19)	0.96 (0.82-1.11)	3395 (24.9)	1020	30	1.25 (1.11-1.41)	1.17 (1.03-1.34)	1715 (31.5)	460	26.8	1.19 (0.99-1.43)	1.00 (0.82-1.23)
Unknown	65 (0.5)	65	100	-	-	10 (0.1)	10	100	-	-	-	-	-	-	-
**Admission with diagnoses within 2 years before 20 weeks gestation**															
Mental health (excluding substance misuse and self-harm)	175 (1.5)	90	51.4	2.61 (1.92-3.54)	1.33 (0.92-1.93)	330 (2.4)	140	42.4	2.21 (1.76-2.77)	1.40 (1.05-1.87)	175 (3.2)	70	40	2.02 (1.48-2.77)	1.43 (0.94-2.17)
Adversity-related	355 (3.0)	190	53.5	2.81 (2.26-3.48)	1.33 (0.99-1.79)	500 (3.7)	220	44	2.32 (1.93-2.80)	1.14 (0.88-1.49)	255 (4.7)	105	41.2	2.22 (1.71-2.89)	1.24 (0.84-1.83)
Any chronic condition	755 (6.3)	345	45.7	2.13 (1.83-2.48)	1.38 (1.12-1.71)	1165 (8.6)	440	37.8	1.77 (1.56-2.02)	1.26 (1.04-1.53)	555 (10.2)	190	34.2	1.64 (1.35-1.99)	1.10 (0.81-1.49)
A&E visits	7795 (64.9)	2665	34.2	1.85 (1.69-2.02)	1.53 (1.39-1.68)	8390 (61.6)	2475	29.5	1.39 (1.28-1.51)	1.24 (1.13-1.36)	3110 (57.2)	885	28.5	1.42 (1.25-1.62)	1.26 (1.09-1.46)
**Gestational age at antenatal booking appointment**															
Before 10 weeks	1725 (14.4)	515	29.9	1 (ref)	1 (ref)	3280 (24.1)	930	28.4	1 (ref)	1 (ref)	2500 (46.0)	645	25.8	1 (ref)	1 (ref)
10-20 weeks	5710 (47.5)	1730	30.3	1.04 (0.92-1.17)	1.02 (0.90-1.16)	5430 (39.9)	1540	28.4	0.95 (0.86-1.05)	0.91 (0.81-1.01)	1995 (36.7)	505	25.3	0.87 (0.76-1.00)	0.79 (0.68-0.92)
20 weeks or more	1060 (8.8)	285	26.9	0.90 (0.75-1.07)	0.82 (0.68-0.99)	610 (4.5)	150	24.6	0.72 (0.59-0.89)	0.64 (0.51-0.80)	240 (4.4)	55	22.9	0.76 (0.55-1.05)	0.64 (0.45-0.90)
Unknown	3515 (29.3)	1075	30.6	1.03 (0.90-1.17)	0.89 (0.77-1.02)	4300 (31.6)	1110	25.8	0.84 (0.75-0.93)	0.71 (0.63-0.81)	700 (12.9)	195	27.9	0.84 (0.69-1.04)	0.74 (0.60-0.93)
**Linked to NPD**															
Linked to NPD	7680 (63.9)	2690	35	1 (ref)	1 (ref)	11810 (86.7)	3445	29.2	1 (ref)	1 (ref)	4795 (88.1)	1295	27	1 (ref)	1 (ref)
Not linked to NPD	4140 (34.5)	865	20.9	0.47 (0.43-0.51)	0.80 (0.68-0.93)	1705 (12.5)	260	15.2	0.43 (0.37-0.49)	0.79 (0.66-0.94)	600 (11.0)	95	15.8	0.44 (0.35-0.55)	0.84 (0.64-1.11)
Linked to NPD but not to NPD census	190 (1.6)	45	23.7	0.56 (0.40-0.79)	0.94 (0.65-1.35)	110 (0.8)	20	18.2	0.55 (0.34-0.88)	0.89 (0.54-1.47)	45 (0.8)	-	-	0.44 (0.20-1.00)	0.80 (0.34-1.88)
**Ever had a child protection plan or was looked after before 20 weeks pregnancy**															
No CPP or looked after	6985 (58.2)	2285	32.7	1 (ref)	1 (ref)	10980 (80.6)	3005	27.4	1 (ref)	1 (ref)	4455 (81.9)	1120	25.1	1 (ref)	1 (ref)

	London					South-East					South-West				
	N eligible mothers	N enrolled in FNP	% enrolled in FNP	Crude odds ratio (95% CI)	Adjusted[a] odds ratio (95% CI)	N eligible mothers	N enrolled in FNP	% enrolled in FNP	Crude odds ratio (95% CI)	Adjusted[a] odds ratio (95% CI)	N eligible mothers	N enrolled in FNP	% enrolled in FNP	Crude odds ratio (95% CI)	Adjusted[a] odds ratio (95% CI)
Looked after (CPP)	760 (6.3)	380	50	2.15 (1.85-2.51)	1.52 (1.29-1.80)	705 (5.2)	330	46.8	2.46 (2.10-2.88)	1.77 (1.48-2.10)	315 (5.8)	155	49.2	3.08 (2.43-3.90)	2.38 (1.83-3.10)
Child protection plan, but not looked after	125 (1.0)	70	56	2.56 (1.78-3.68)	1.60 (1.08-2.35)	230 (1.7)	130	56.5	3.54 (2.69-4.66)	1.76 (1.31-2.36)	70 (1.3)	30	42.9	2.31 (1.42-3.74)	1.09 (0.65-1.82)
Not linked to NPD	4140 (34.5)	865	20.9	0.52 (0.48-0.58)	-[c]	1705 (12.5)	260	15.2	0.47 (0.41-0.54)	-[c]	600 (11.0)	95	15.8	0.49 (0.38-0.62)	-[c]
**Ever recorded as having Special Educational Needs before 20 weeks pregnancy**															
No	3020 (25.1)	910	30.1	1 (ref)	1 (ref)	5160 (37.9)	1235	23.9	1 (ref)	1 (ref)	2575 (47.3)	545	21.2	1 (ref)	1 (ref)
Yes	4660 (38.8)	1780	38.2	1.45 (1.31-1.60)	1.19 (1.07-1.33)	6650 (48.8)	2210	33.2	1.65 (1.52-1.80)	1.20 (1.09-1.33)	2220 (40.8)	750	33.8	1.87 (1.63-2.13)	1.42 (1.22-1.66)
Not linked to NPD	4140 (34.5)	865	20.9	0.59 (0.53-0.66)	-[c]	1705 (12.5)	260	15.2	0.58 (0.50-0.67)	-[c]	600 (11.0)	95	15.8	0.60 (0.47-0.77)	-[c]
Linked to NPD but not to NPD census	190 (1.6)	45	23.7	0.70 (0.50-1.00)	-[c]	110 (0.8)	20	18.2	0.73 (0.45-1.18)	-[c]	45 (0.8)	-	-	0.60 (0.27-1.37)	-[c]
**Ever recorded as receiving free school meals before 20 weeks of pregnancy**															
No	2655 (22.1)	820	30.9	1 (ref)	1 (ref)	6080 (44.6)	1440	23.7	1 (ref)	1 (ref)	2575 (47.3)	560	21.7	1 (ref)	1 (ref)
Yes	5020 (41.8)	1870	37.3	1.29 (1.17-1.43)	1.03 (0.92-1.15)	5730 (42.1)	2010	35.1	1.77 (1.63-1.92)	1.21 (1.10-1.33)	2220 (40.8)	740	33.3	1.79 (1.57-2.04)	1.18 (1.02-1.38)
Not linked to NPD	4140 (34.5)	865	20.9	0.56 (0.50-0.63)	-[c]	1705 (12.5)	260	15.2	0.57 (0.50-0.67)	-[c]	600 (11.0)	95	15.8	0.59 (0.46-0.75)	-[c]
Linked to NPD but not to NPD census	190 (1.6)	45	23.7	0.66 (0.47-0.94)	-[c]	110 (0.8)	20	18.2	0.73 (0.45-1.18)	-[c]	45 (0.8)	-	-	0.59 (0.26-1.33)	-[c]
**Ever in IDACI bottom decile before 20 weeks of pregnancy**															
No	3355 (27.9)	1065	31.7	1 (ref)	1 (ref)	9775 (71.7)	2755	28.2	1 (ref)	1 (ref)	3860 (71.0)	1015	26.3	1 (ref)	1 (ref)
Yes	4325 (36.0)	1625	37.6	1.24 (1.12-1.37)	1.11 (0.99-1.24)	2035 (14.9)	695	34.2	1.40 (1.25-1.56)	0.99 (0.88-1.12)	935 (17.2)	280	29.9	1.41 (1.19-1.67)	1.06 (0.88-1.29)
Not linked to NPD	4140 (34.5)	865	20.9	0.54 (0.48-0.60)	-[c]	1705 (12.5)	260	15.2	0.46 (0.40-0.52)	-[c]	600 (11.0)	95	15.8	0.47 (0.37-0.59)	-[c]
Linked to NPD but not to NPD census	190 (1.6)	45	23.7	0.64 (0.45-0.90)	-[c]	110 (0.8)	20	18.2	0.58 (0.36-0.94)	-[c]	45 (0.8)	-	-	0.47 (0.21-1.08)	-[c]
**Educational attainment before 20 weeks of pregnancy**															
Attempted but did not achieve 5 A*-C GCSEs	5970 (49.7)	2010	33.7	1 (ref)	1 (ref)	9005 (66.1)	2530	28.1	1 (ref)	1 (ref)	3620 (66.5)	940	26	1 (ref)	1 (ref)
5 A*-C GCSEs	1355 (11.3)	400	29.5	0.82 (0.72-0.93)	1.00 (0.87-1.15)	2120 (15.6)	480	22.6	0.72 (0.65-0.81)	1.01 (0.89-1.14)	935 (17.2)	200	21.4	0.80 (0.67-0.96)	1.13 (0.93-1.37)
Not linked to NPD	4140 (34.5)	865	20.9	0.50 (0.46-0.55)	-[c]	1705 (12.5)	260	15.2	0.45 (0.39-0.52)	-[c]	600 (11.0)	95	15.8	0.47 (0.37-0.60)	-[c]
Had not attempted GCSEs prior to 20 weeks pregnancy	540 (4.5)	325	60.2	3.04 (2.53-3.65)	1.41 (1.13-1.76)	795 (5.8)	460	57.9	3.95 (3.39-4.60)	1.44 (1.19-1.74)	290 (5.3)	165	56.9	4.46 (3.47-5.74)	1.72 (1.25-2.36)
**Ever excluded, in pupil referral unit, or alternative provision**															
No	5130 (42.7)	1645	32.1	1 (ref)	1 (ref)	7900 (58.0)	2060	26.1	1 (ref)	1 (ref)	3630 (66.7)	865	23.8	1 (ref)	1 (ref)
Yes	2735 (22.8)	1090	39.9	1.42 (1.28-1.56)	1.05 (0.94-1.17)	4020 (29.5)	1410	35.1	1.59 (1.46-1.73)	1.06 (0.96-1.16)	1215 (22.3)	435	35.8	1.71 (1.48-1.97)	1.04 (0.88-1.22)
Not linked to NPD	4140 (34.5)	865	20.9	0.54 (0.49-0.60)	-[c]	1705 (12.5)	260	15.2	0.51 (0.44-0.59)	-[c]	600 (11.0)	95	15.8	0.51 (0.40-0.65)	-[c]
**Ever persistently absent in a term (>=10% possible sessions)**															
No	5215 (43.4)	1510	29	1 (ref)	1 (ref)	7475 (54.9)	1560	20.9	1 (ref)	1 (ref)	3205 (58.9)	645	20.1	1 (ref)	1 (ref)
Yes	2655 (22.1)	1225	46.1	2.19 (1.98-2.41)	1.38 (1.22-1.55)	4445 (32.6)	1910	43	3.06 (2.81-3.33)	1.62 (1.46-1.80)	1640 (30.1)	660	40.2	2.86 (2.49-3.27)	1.51 (1.27-1.79)
Not linked to NPD	4140 (34.5)	865	20.9	0.63 (0.57-0.69)	-[c]	1705 (12.5)	260	15.2	0.69 (0.60-0.80)	-[c]	600 (11.0)	95	15.8	0.65 (0.51-0.83)	-[c]

[a]Adjusted models included all variables in the table as covariates

[b]Includes only mothers aged 19 at last menstrual period

[c]Estimates omitted due to multicollinearity

Note: numbers have been rounded to the nearest 5 in accordance with NHS Digital's statistical disclosure rules for sub-national analyses. NPD – National Pupil Database

Table S7. Predictors of enrolment in the Family Nurse Partnership by financial year, among mothers aged 13-19 at last menstrual period, living in a Local Authority with an active FNP site at the time of first antenatal appointment – England, births between April 2010-March 2017

	2010-11					2011-12					2012-13				
	N eligible mothers	N enrolled in FNP	% enrolled in FNP	Crude odds ratio (95% CI)	Adjusted[a] odds ratio (95% CI)	N eligible mothers	N enrolled in FNP	% enrolled in FNP	Crude odds ratio (95% CI)	Adjusted[a] odds ratio (95% CI)	N eligible mothers	N enrolled in FNP	% enrolled in FNP	Crude odds ratio (95% CI)	Adjusted[a] odds ratio (95% CI)
**Total**	13,360	2,770	20.7	-	-	13,820	1,605	11.6	-	-	16,225	3,305	20.4	-	-
**Maternal age at birth**															
13-15	270 (2.0)	115	42.6	3.63 (2.78-4.76)	2.23 (1.61-3.09)	320 (2.3)	110	34.4	6.46 (4.85-8.59)	2.92 (2.04-4.16)	390 (2.4)	205	52.6	6.17 (4.96-7.68)	3.55 (2.70-4.67)
16-17	2900 (21.7)	920	31.7	2.01 (1.81-2.23)	1.52 (1.34-1.73)	2960 (21.4)	655	22.1	3.24 (2.85-3.69)	2.06 (1.76-2.42)	3450 (21.3)	1,290	37.4	3.19 (2.90-3.50)	2.21 (1.98-2.48)
18-19	7345 (55.0)	1,455	19.8	1 (ref)	1 (ref)	7500 (54.3)	705	9.4	1 (ref)	1 (ref)	8875 (54.7)	1,560	17.6	1 (ref)	1 (ref)
20[b]	2845 (21.3)	280	9.8	0.42 (0.37-0.49)	0.49 (0.42-0.56)	3040 (22.0)	135	4.4	0.42 (0.34-0.51)	0.52 (0.42-0.64)	3510 (21.6)	255	7.3	0.35 (0.31-0.41)	0.44 (0.38-0.51)
**Ethnicity**															
White	11485 (86.0)	2,330	20.3	1 (ref)	1 (ref)	11880 (86.0)	1,305	11	1 (ref)	1 (ref)	14065 (86.7)	2,890	20.5	1 (ref)	1 (ref)
South Asian	365 (2.7)	50	13.7	0.42 (0.30-0.58)	0.58 (0.41-0.83)	315 (2.3)	40	12.7	0.69 (0.47-1.02)	0.81 (0.53-1.26)	415 (2.6)	65	15.7	0.57 (0.42-0.77)	0.84 (0.60-1.17)
Black	585 (4.4)	190	32.5	1.32 (1.06-1.64)	1.47 (1.17-1.85)	625 (4.5)	125	20	1.24 (0.97-1.58)	1.29 (0.98-1.68)	570 (3.5)	130	22.8	1.10 (0.87-1.40)	1.31 (1.01-1.70)
Mixed/other	640 (4.8)	150	23.4	0.91 (0.73-1.12)	0.98 (0.79-1.23)	695 (5.0)	95	13.7	0.90 (0.70-1.16)	0.95 (0.73-1.25)	745 (4.6)	160	21.5	1.05 (0.86-1.27)	1.09 (0.88-1.35)
Unknown	280 (2.1)	50	17.9	0.75 (0.55-1.04)	0.90 (0.64-1.27)	310 (2.2)	35	11.3	0.87 (0.60-1.27)	1.06 (0.71-1.60)	430 (2.7)	60	14	0.63 (0.48-0.84)	0.74 (0.54-1.00)
**Index of Multiple Deprivation (quintile)**															
Least deprived	560 (4.2)	95	17	0.76 (0.58-1.00)	0.78 (0.59-1.03)	555 (4.0)	40	7.2	0.52 (0.36-0.77)	0.52 (0.35-0.79)	770 (4.7)	110	14.3	0.83 (0.65-1.05)	0.90 (0.70-1.16)
2	875 (6.5)	155	17.7	0.95 (0.75-1.19)	0.94 (0.74-1.19)	950 (6.9)	95	10	0.97 (0.74-1.28)	0.95 (0.71-1.28)	1310 (8.1)	230	17.6	1.01 (0.84-1.22)	1.09 (0.89-1.33)
3	1570 (11.8)	290	18.5	1 (ref)	1 (ref)	1770 (12.8)	195	11	1 (ref)	1 (ref)	2195 (13.5)	390	17.8	1 (ref)	1 (ref)
4	3260 (24.4)	695	21.3	1.27 (1.08-1.50)	1.19 (1.01-1.41)	3505 (25.4)	425	12.1	1.21 (0.99-1.47)	1.10 (0.89-1.35)	4010 (24.7)	825	20.6	1.27 (1.10-1.46)	1.24 (1.07-1.45)
Most deprived	7085 (53.0)	1,520	21.5	1.45 (1.24-1.69)	1.33 (1.13-1.56)	7035 (50.9)	840	11.9	1.17 (0.97-1.42)	0.97 (0.79-1.19)	7930 (48.9)	1,740	21.9	1.49 (1.30-1.70)	1.36 (1.17-1.58)
Unknown	10 (0.1)	10	100	-	-	-	-	-	-	-	10 (0.1)	10	100	-	-
**Admission with diagnoses within 2 years before 20 weeks gestation**															
Mental health (excluding substance misuse and self-harm)	155 (1.2)	50	32.3	1.91 (1.33-2.74)	1.35 (0.88-2.06)	210 (1.5)	35	16.7	1.74 (1.17-2.60)	0.98 (0.60-1.58)	295 (1.8)	90	30.5	1.71 (1.31-2.22)	1.06 (0.76-1.47)
Adversity-related	485 (3.6)	150	30.9	1.90 (1.54-2.34)	0.97 (0.71-1.31)	515 (3.7)	105	20.4	2.46 (1.93-3.14)	1.54 (1.07-2.22)	620 (3.8)	220	35.5	2.42 (2.02-2.89)	1.30 (1.00-1.70)
Any chronic condition	930 (7.0)	265	28.5	1.64 (1.40-1.92)	1.25 (0.99-1.58)	1090 (7.9)	180	16.5	1.67 (1.39-2.01)	1.13 (0.85-1.50)	1350 (8.3)	385	28.5	1.70 (1.49-1.94)	1.18 (0.97-1.45)
A&E visits	7600 (56.9)	1,775	23.4	1.50 (1.37-1.64)	1.37 (1.24-1.51)	8340 (60.3)	1,075	12.9	1.34 (1.19-1.51)	1.20 (1.05-1.37)	10005 (61.7)	2,275	22.7	1.54 (1.42-1.68)	1.38 (1.25-1.52)
**Gestational age at antenatal booking appointment**															
Before 10 weeks	2985 (22.3)	615	20.6	1 (ref)	1 (ref)	3585 (25.9)	390	10.9	1 (ref)	1 (ref)	4555 (28.1)	905	19.9	1 (ref)	1 (ref)
10-20 weeks	5325 (39.9)	1,100	20.7	0.95 (0.84-1.07)	0.94 (0.83-1.07)	5055 (36.6)	590	11.7	1.06 (0.91-1.24)	1.04 (0.89-1.23)	6150 (37.9)	1,290	21	0.96 (0.87-1.06)	0.88 (0.78-0.98)
20 weeks or more	910 (6.8)	170	18.7	0.75 (0.61-0.93)	0.65 (0.53-0.81)	815 (5.9)	145	17.8	1.48 (1.17-1.88)	1.10 (0.85-1.42)	920 (5.7)	200	21.7	0.93 (0.78-1.12)	0.68 (0.56-0.84)
Unknown	4135 (31.0)	880	21.3	0.87 (0.75-1.01)	0.81 (0.69-0.95)	4365 (31.6)	475	10.9	1.02 (0.85-1.23)	0.94 (0.77-1.14)	4600 (28.4)	910	19.8	1.01 (0.89-1.15)	0.82 (0.71-0.94)
**Linked to NPD**															
Linked to NPD	10995 (82.3)	2,400	21.8	1 (ref)	1 (ref)	11495 (83.2)	1,410	12.3	1 (ref)	1 (ref)	13595 (83.8)	2,985	22	1 (ref)	1 (ref)
Not linked to NPD	2175 (16.3)	330	15.2	0.52 (0.45-0.59)	0.79 (0.67-0.94)	2190 (15.8)	175	8	0.44 (0.37-0.53)	0.88 (0.69-1.11)	2515 (15.5)	300	11.9	0.44 (0.38-0.50)	0.80 (0.67-0.96)
Linked to NPD but not to NPD census	190 (1.4)	35	18.4	0.71 (0.48-1.04)	1.11 (0.74-1.65)	135 (1.0)	15	11.1	0.59 (0.32-1.07)	1.04 (0.55-1.97)	120 (0.7)	20	16.7	0.59 (0.35-0.99)	0.96 (0.55-1.67)
**Ever had a child protection plan or was looked after before 20 weeks pregnancy**															

	2010-11					2011-12					2012-13				
	**N eligible mothers**	**N enrolled in FNP**	**% enrolled in FNP**	**Crude odds ratio (95% CI)**	**Adjusted[a] odds ratio (95% CI)**	**N eligible mothers**	**N enrolled in FNP**	**% enrolled in FNP**	**Crude odds ratio (95% CI)**	**Adjusted[a] odds ratio (95% CI)**	**N eligible mothers**	**N enrolled in FNP**	**% enrolled in FNP**	**Crude odds ratio (95% CI)**	**Adjusted[a] odds ratio (95% CI)**
No CPP or looked after	10690 (80.0)	2,260	21.1	1 (ref)	1 (ref)	11030 (79.8)	1,260	11.4	1 (ref)	1 (ref)	12865 (79.3)	2,660	20.7	1 (ref)	1 (ref)
Looked after (CPP)	480 (3.6)	175	36.5	2.00 (1.63-2.46)	1.47 (1.18-1.83)	545 (3.9)	150	27.5	3.07 (2.46-3.84)	2.33 (1.83-2.98)	730 (4.5)	295	40.4	2.95 (2.51-3.48)	2.24 (1.87-2.68)
Child protection plan, but not looked after	10 (0.1)	-	-	2.72 (0.79-9.30)	1.40 (0.39-5.04)	50 (0.4)	15	30	3.02 (1.58-5.80)	1.18 (0.59-2.33)	120 (0.7)	50	41.7	3.18 (2.17-4.66)	1.42 (0.94-2.14)
Not linked to NPD	2175 (16.3)	330	15.2	0.55 (0.48-0.62)	-[c]	2190 (15.8)	175	8	0.48 (0.40-0.58)	-[c]	2515 (15.5)	300	11.9	0.48 (0.42-0.55)	-[c]
**Ever recorded as having Special Educational Needs before 20 weeks pregnancy**															
No	6955 (52.1)	1,320	19	1 (ref)	1 (ref)	6555 (47.4)	665	10.1	1 (ref)	1 (ref)	6955 (42.9)	1,200	17.3	1 (ref)	1 (ref)
Yes	4040 (30.2)	1,085	26.9	1.55 (1.41-1.71)	1.14 (1.03-1.27)	4935 (35.7)	745	15.1	1.66 (1.47-1.87)	1.07 (0.93-1.23)	6640 (40.9)	1,785	26.9	1.76 (1.61-1.92)	1.25 (1.13-1.38)
Not linked to NPD	2175 (16.3)	330	15.2	0.63 (0.54-0.72)	-[c]	2190 (15.8)	175	8	0.56 (0.46-0.68)	-[c]	2515 (15.5)	300	11.9	0.59 (0.51-0.69)	-[c]
Linked to NPD but not to NPD census	190 (1.4)	35	18.4	0.85 (0.58-1.25)	-[c]	135 (1.0)	15	11.1	0.75 (0.41-1.37)	-[c]	120 (0.7)	20	16.7	0.80 (0.47-1.35)	-[c]
**Ever recorded as receiving free school meals before 20 weeks of pregnancy**															
No	6730 (50.4)	1,275	18.9	1 (ref)	1 (ref)	6385 (46.2)	550	8.6	1 (ref)	1 (ref)	6835 (42.1)	1,165	17	1 (ref)	1 (ref)
Yes	4265 (31.9)	1,125	26.4	1.43 (1.30-1.58)	1.01 (0.91-1.13)	5105 (36.9)	860	16.8	2.18 (1.92-2.48)	1.34 (1.16-1.55)	6760 (41.7)	1,820	26.9	1.85 (1.70-2.02)	1.11 (1.00-1.23)
Not linked to NPD	2175 (16.3)	330	15.2	0.61 (0.53-0.70)	-[c]	2190 (15.8)	175	8	0.68 (0.55-0.82)	-[c]	2515 (15.5)	300	11.9	0.61 (0.53-0.71)	-[c]
Linked to NPD but not to NPD census	190 (1.4)	35	18.4	0.83 (0.56-1.22)	-[c]	135 (1.0)	15	11.1	0.87 (0.47-1.59)	-[c]	120 (0.7)	20	16.7	0.82 (0.48-1.39)	-[c]
**Ever in IDACI bottom decile before 20 weeks of pregnancy**															
No	7475 (56.0)	1,530	20.5	1 (ref)	1 (ref)	7700 (55.7)	820	10.6	1 (ref)	1 (ref)	9075 (55.9)	1,845	20.3	1 (ref)	1 (ref)
Yes	3520 (26.3)	870	24.7	1.31 (1.17-1.46)	1.05 (0.93-1.18)	3795 (27.5)	595	15.7	1.41 (1.23-1.62)	1.15 (0.99-1.34)	4520 (27.9)	1,145	25.3	1.33 (1.21-1.46)	0.99 (0.89-1.11)
Not linked to NPD	2175 (16.3)	330	15.2	0.58 (0.50-0.67)	-[c]	2190 (15.8)	175	8	0.52 (0.43-0.63)	-[c]	2515 (15.5)	300	11.9	0.49 (0.42-0.56)	-[c]
Linked to NPD but not to NPD census	190 (1.4)	35	18.4	0.78 (0.53-1.15)	-[c]	135 (1.0)	15	11.1	0.67 (0.36-1.22)	-[c]	120 (0.7)	20	16.7	0.65 (0.39-1.10)	-[c]
**Educational attainment before 20 weeks of pregnancy**															
Attempted but did not achieve 5 A*-C GCSEs	8845 (66.2)	1,830	20.7	1 (ref)	1 (ref)	9015 (65.2)	985	10.9	1 (ref)	1 (ref)	10285 (63.4)	2,090	20.3	1 (ref)	1 (ref)
5 A*-C GCSEs	1470 (11.0)	255	17.3	0.84 (0.72-0.97)	1.06 (0.90-1.24)	1730 (12.5)	150	8.7	0.78 (0.64-0.95)	0.99 (0.80-1.22)	2360 (14.5)	380	16.1	0.75 (0.66-0.85)	1.00 (0.87-1.15)
Not linked to NPD	2175 (16.3)	330	15.2	0.56 (0.49-0.65)	-[c]	2190 (15.8)	175	8	0.51 (0.42-0.61)	-[c]	2515 (15.5)	300	11.9	0.48 (0.42-0.56)	-[c]
Had not attempted GCSEs prior to 20 weeks pregnancy	870 (6.5)	350	40.2	2.87 (2.46-3.36)	1.43 (1.18-1.74)	885 (6.4)	290	32.8	4.89 (4.09-5.86)	1.70 (1.35-2.13)	1065 (6.6)	535	50.2	4.50 (3.92-5.16)	1.53 (1.28-1.82)
**Ever excluded, in pupil referral unit, or alternative provision**															
No	8625 (64.6)	1,705	19.8	1 (ref)	1 (ref)	8450 (61.1)	925	10.9	1 (ref)	1 (ref)	9655 (59.5)	1,905	19.7	1 (ref)	1 (ref)
Yes	2560 (19.2)	735	28.7	1.63 (1.46-1.81)	1.06 (0.95-1.20)	3180 (23.0)	505	15.9	1.55 (1.36-1.76)	0.94 (0.81-1.08)	4060 (25.0)	1,100	27.1	1.57 (1.43-1.71)	1.01 (0.91-1.12)
Not linked to NPD	2175 (16.3)	330	15.2	0.59 (0.52-0.68)	-[c]	2190 (15.8)	175	8	0.51 (0.42-0.61)	-[c]	2515 (15.5)	300	11.9	0.51 (0.44-0.58)	-[c]
**Ever persistently absent in a term (>=10% possible sessions)**															
No	6520 (48.8)	1,050	16.1	1 (ref)	1 (ref)	6990 (50.6)	525	7.5	1 (ref)	1 (ref)	8290 (51.1)	1,130	13.6	1 (ref)	1 (ref)
Yes	4665 (34.9)	1,390	29.8	2.36 (2.14-2.60)	1.38 (1.22-1.55)	4635 (33.5)	900	19.4	3.62 (3.19-4.11)	1.67 (1.42-1.96)	5420 (33.4)	1,875	34.6	3.73 (3.41-4.08)	1.74 (1.56-1.94)
Not linked to NPD	2175 (16.3)	330	15.2	0.77 (0.67-0.89)	-[c]	2190 (15.8)	175	8	0.80 (0.66-0.98)	-[c]	2515 (15.5)	300	11.9	0.79 (0.68-0.91)	-[c]

Table S7. Predictors of enrolment in the Family Nurse Partnership by financial year, among mothers aged 13-19 at last menstrual period, living in a Local Authority with an active FNP site at the time of first antenatal appointment – England, births between April 2010-March 2017 (continued)

	2013-14					2014-15					2015-16				
	N eligible mothers	N enrolled in FNP	% enrolled in FNP	Crude odds ratio (95% CI)	Adjusted[a] odds ratio (95% CI)	N eligible mothers	N enrolled in FNP	% enrolled in FNP	Crude odds ratio (95% CI)	Adjusted[a] odds ratio (95% CI)	N eligible mothers	N enrolled in FNP	% enrolled in FNP	Crude odds ratio (95% CI)	Adjusted[a] odds ratio (95% CI)
**Total**	16,910	4,070	24.1	-	-	16,275	3,740	23	-	-	18,415	5,590	30.4	-	-
**Maternal age at birth**															
13-15	400 (2.4)	230	57.5	5.71 (4.61-7.08)	3.38 (2.59-4.42)	340 (2.1)	180	52.9	5.47 (4.31-6.93)	3.10 (2.31-4.16)	400 (2.2)	240	60	4.54 (3.67-5.61)	2.40 (1.85-3.12)
16-17	3530 (20.9)	1,425	40.4	2.68 (2.46-2.93)	1.86 (1.67-2.07)	3340 (20.5)	1,270	38	2.67 (2.43-2.93)	1.98 (1.77-2.22)	3665 (19.9)	1,735	47.3	2.50 (2.30-2.71)	1.75 (1.58-1.94)
18-19	9295 (55.0)	2,010	21.6	1 (ref)	1 (ref)	9010 (55.4)	1,870	20.8	1 (ref)	1 (ref)	10280 (55.8)	2,900	28.2	1 (ref)	1 (ref)
20[b]	3685 (21.8)	405	11	0.44 (0.39-0.49)	0.52 (0.46-0.59)	3590 (22.1)	415	11.6	0.47 (0.42-0.53)	0.53 (0.47-0.61)	4065 (22.1)	715	17.6	0.50 (0.46-0.56)	0.58 (0.52-0.64)
**Ethnicity**															
White	14525 (85.9)	3,565	24.5	1 (ref)	1 (ref)	13665 (84.0)	3,135	22.9	1 (ref)	1 (ref)	15380 (83.5)	4,780	31.1	1 (ref)	1 (ref)
South Asian	495 (2.9)	70	14.1	0.44 (0.33-0.58)	0.65 (0.48-0.87)	545 (3.3)	80	14.7	0.48 (0.37-0.63)	0.66 (0.50-0.87)	555 (3.0)	130	23.4	0.58 (0.47-0.72)	0.79 (0.63-0.99)
Black	545 (3.2)	155	28.4	1.16 (0.93-1.44)	1.37 (1.08-1.75)	550 (3.4)	180	32.7	1.17 (0.95-1.45)	1.27 (1.01-1.59)	590 (3.2)	215	36.4	1.18 (0.98-1.43)	1.34 (1.09-1.65)
Mixed/other	845 (5.0)	195	23.1	0.89 (0.75-1.07)	1.08 (0.89-1.30)	895 (5.5)	220	24.6	0.88 (0.74-1.05)	0.96 (0.79-1.15)	1005 (5.5)	255	25.4	0.69 (0.59-0.81)	0.73 (0.62-0.87)
Unknown	500 (3.0)	85	17	0.54 (0.42-0.69)	0.62 (0.47-0.81)	625 (3.8)	125	20	0.65 (0.52-0.80)	0.81 (0.64-1.01)	885 (4.8)	205	23.2	0.63 (0.53-0.75)	0.78 (0.65-0.93)
**Index of Multiple Deprivation (quintile)**															
Least deprived	890 (5.3)	210	23.6	0.81 (0.67-0.98)	0.94 (0.76-1.15)	815 (5.0)	145	17.8	0.78 (0.63-0.98)	0.82 (0.65-1.04)	1060 (5.8)	265	25	0.70 (0.59-0.84)	0.70 (0.59-0.84)
2	1345 (8.0)	300	22.3	0.83 (0.70-0.98)	0.90 (0.76-1.08)	1265 (7.8)	265	20.9	0.92 (0.77-1.10)	0.95 (0.78-1.14)	1500 (8.1)	405	27	0.86 (0.74-0.99)	0.89 (0.76-1.04)
3	2345 (13.9)	575	24.5	1 (ref)	1 (ref)	2145 (13.2)	465	21.7	1 (ref)	1 (ref)	2590 (14.1)	790	30.5	1 (ref)	1 (ref)
4	4130 (24.4)	1,015	24.6	1.01 (0.89-1.14)	0.98 (0.86-1.12)	4135 (25.4)	960	23.2	1.05 (0.91-1.20)	1.00 (0.87-1.16)	4640 (25.2)	1,365	29.4	0.94 (0.84-1.05)	0.91 (0.81-1.02)
Most deprived	8185 (48.4)	1,955	23.9	1.09 (0.97-1.22)	1.01 (0.89-1.15)	7890 (48.5)	1,880	23.8	1.19 (1.05-1.35)	1.08 (0.94-1.24)	8595 (46.7)	2,745	31.9	1.09 (0.98-1.21)	1.02 (0.91-1.14)
Unknown	15 (0.1)	15	100	-	-	25 (0.2)	25	100	-	-	25 (0.1)	25	100	-	-
**Admission with diagnoses within 2 years before 20 weeks gestation**															
Mental health (excluding substance misuse and self-harm)	310 (1.8)	125	40.3	2.27 (1.79-2.88)	1.88 (1.39-2.53)	370 (2.3)	145	39.2	2.49 (1.99-3.12)	1.61 (1.21-2.13)	555 (3.0)	260	46.8	2.16 (1.81-2.58)	1.37 (1.09-1.73)
Adversity-related	605 (3.6)	225	37.2	2.06 (1.73-2.45)	1.33 (1.03-1.72)	670 (4.1)	270	40.3	2.59 (2.19-3.07)	1.38 (1.08-1.77)	825 (4.5)	415	50.3	2.62 (2.26-3.04)	1.30 (1.06-1.61)
Any chronic condition	1375 (8.1)	415	30.2	1.46 (1.29-1.66)	0.89 (0.74-1.08)	1430 (8.8)	465	32.5	1.80 (1.59-2.03)	1.09 (0.90-1.32)	1790 (9.7)	755	42.2	1.83 (1.65-2.03)	1.18 (1.01-1.38)
A&E visits	10615 (62.8)	2,775	26.1	1.37 (1.27-1.48)	1.25 (1.14-1.36)	10380 (63.8)	2,635	25.4	1.47 (1.36-1.60)	1.25 (1.14-1.37)	11865 (64.4)	3,960	33.4	1.51 (1.41-1.63)	1.31 (1.21-1.42)
**Gestational age at antenatal booking appointment**															
Before 10 weeks	4470 (26.4)	1,060	23.7	1 (ref)	1 (ref)	4350 (26.7)	995	22.9	1 (ref)	1 (ref)	4940 (26.8)	1,550	31.4	1 (ref)	1 (ref)
10-20 weeks	5865 (34.7)	1,430	24.4	0.96 (0.87-1.06)	0.91 (0.82-1.01)	5670 (34.8)	1,365	24.1	0.91 (0.82-1.01)	0.87 (0.78-0.96)	6715 (36.5)	2,105	31.3	0.92 (0.84-1.00)	0.89 (0.81-0.97)
20 weeks or more	955 (5.6)	295	30.9	1.13 (0.96-1.34)	0.88 (0.73-1.06)	800 (4.9)	195	24.4	0.76 (0.62-0.92)	0.61 (0.49-0.75)	940 (5.1)	265	28.2	0.76 (0.64-0.89)	0.65 (0.54-0.77)
Unknown	5620 (33.2)	1,280	22.8	0.88 (0.78-0.99)	0.75 (0.66-0.86)	5455 (33.5)	1,180	21.6	0.84 (0.75-0.95)	0.73 (0.64-0.83)	5815 (31.6)	1,670	28.7	0.83 (0.75-0.92)	0.76 (0.68-0.85)
**Linked to NPD**															
Linked to NPD	14075 (83.2)	3,665	26	1 (ref)	1 (ref)	13815 (84.9)	3,385	24.5	1 (ref)	1 (ref)	15435 (83.8)	5,035	32.6	1 (ref)	1 (ref)
Not linked to NPD	2720 (16.1)	390	14.3	0.41 (0.37-0.47)	0.78 (0.67-0.91)	2360 (14.5)	340	14.4	0.42 (0.37-0.48)	0.77 (0.65-0.92)	2870 (15.6)	540	18.8	0.45 (0.40-0.50)	0.79 (0.68-0.91)
Linked to NPD but not to NPD census	115 (0.7)	15	13	0.32 (0.17-0.57)	0.54 (0.29-1.00)	105 (0.6)	15	14.3	0.40 (0.22-0.70)	0.57 (0.31-1.06)	110 (0.6)	10	9.1	0.23 (0.13-0.43)	0.33 (0.18-0.63)
**Ever had a child protection plan or was looked after before 20 weeks pregnancy**															

	2013-14					2014-15					2015-16				
	**N eligible mothers**	**N enrolled in FNP**	**% enrolled in FNP**	**Crude odds ratio (95% CI)**	**Adjusted[a] odds ratio (95% CI)**	**N eligible mothers**	**N enrolled in FNP**	**% enrolled in FNP**	**Crude odds ratio (95% CI)**	**Adjusted[a] odds ratio (95% CI)**	**N eligible mothers**	**N enrolled in FNP**	**% enrolled in FNP**	**Crude odds ratio (95% CI)**	**Adjusted[a] odds ratio (95% CI)**
No CPP or looked after	13160 (77.8)	3,220	24.5	1 (ref)	1 (ref)	12715 (78.1)	2,900	22.8	1 (ref)	1 (ref)	13970 (75.9)	4,195	30	1 (ref)	1 (ref)
Looked after (CPP)	815 (4.8)	360	44.2	2.57 (2.21-2.99)	2.01 (1.71-2.37)	925 (5.7)	380	41.1	2.68 (2.31-3.10)	1.99 (1.69-2.33)	1095 (5.9)	610	55.7	3.07 (2.70-3.50)	2.36 (2.05-2.72)
Child protection plan, but not looked after	215 (1.3)	95	44.2	2.69 (2.02-3.57)	1.37 (1.01-1.86)	280 (1.7)	120	42.9	3.09 (2.39-3.99)	1.70 (1.30-2.23)	475 (2.6)	240	50.5	2.69 (2.22-3.26)	1.52 (1.23-1.87)
Not linked to NPD	2720 (16.1)	390	14.3	0.45 (0.40-0.51)	-[c]	2360 (14.5)	340	14.4	0.47 (0.41-0.54)	-[c]	2870 (15.6)	540	18.8	0.51 (0.46-0.57)	-[c]
**Ever recorded as having Special Educational Needs before 20 weeks pregnancy**															
No	6690 (39.6)	1,430	21.4	1 (ref)	1 (ref)	6080 (37.4)	1,265	20.8	1 (ref)	1 (ref)	6625 (36.0)	1,875	28.3	1 (ref)	1 (ref)
Yes	7385 (43.7)	2,235	30.3	1.63 (1.50-1.76)	1.25 (1.14-1.37)	7735 (47.5)	2,120	27.4	1.49 (1.37-1.62)	1.17 (1.07-1.29)	8810 (47.8)	3,160	35.9	1.46 (1.36-1.57)	1.15 (1.06-1.25)
Not linked to NPD	2720 (16.1)	390	14.3	0.54 (0.48-0.62)	-[c]	2360 (14.5)	340	14.4	0.54 (0.47-0.62)	-[c]	2870 (15.6)	540	18.8	0.56 (0.50-0.63)	-[c]
Linked to NPD but not to NPD census	115 (0.7)	15	13	0.42 (0.23-0.76)	-[c]	105 (0.6)	15	14.3	0.51 (0.29-0.90)	-[c]	110 (0.6)	10	9.1	0.29 (0.16-0.54)	-[c]
**Ever recorded as receiving free school meals before 20 weeks of pregnancy**															
No	6120 (36.2)	1,265	20.7	1 (ref)	1 (ref)	5230 (32.1)	1,025	19.6	1 (ref)	1 (ref)	5640 (30.6)	1,565	27.7	1 (ref)	1 (ref)
Yes	7955 (47.0)	2,400	30.2	1.75 (1.61-1.90)	1.22 (1.11-1.34)	8580 (52.7)	2,360	27.5	1.54 (1.41-1.68)	1.13 (1.02-1.25)	9795 (53.2)	3,470	35.4	1.45 (1.35-1.56)	1.08 (0.99-1.18)
Not linked to NPD	2720 (16.1)	390	14.3	0.58 (0.51-0.66)	-[c]	2360 (14.5)	340	14.4	0.56 (0.49-0.65)	-[c]	2870 (15.6)	540	18.8	0.57 (0.51-0.64)	-[c]
Linked to NPD but not to NPD census	115 (0.7)	15	13	0.44 (0.24-0.81)	-[c]	105 (0.6)	15	14.3	0.53 (0.30-0.94)	-[c]	110 (0.6)	10	9.1	0.30 (0.16-0.55)	-[c]
**Ever in IDACI bottom decile before 20 weeks of pregnancy**															
No	9290 (54.9)	2,330	25.1	1 (ref)	1 (ref)	8670 (53.3)	1,895	21.9	1 (ref)	1 (ref)	9635 (52.3)	2,975	30.9	1 (ref)	1 (ref)
Yes	4785 (28.3)	1,335	27.9	1.22 (1.12-1.33)	0.95 (0.86-1.05)	5140 (31.6)	1,490	29	1.42 (1.30-1.56)	1.12 (1.01-1.24)	5800 (31.5)	2,060	35.5	1.22 (1.13-1.32)	0.98 (0.90-1.07)
Not linked to NPD	2720 (16.1)	390	14.3	0.45 (0.39-0.51)	-[c]	2360 (14.5)	340	14.4	0.50 (0.43-0.57)	-[c]	2870 (15.6)	540	18.8	0.49 (0.44-0.54)	-[c]
Linked to NPD but not to NPD census	115 (0.7)	15	13	0.34 (0.19-0.62)	-[c]	105 (0.6)	15	14.3	0.47 (0.26-0.83)	-[c]	110 (0.6)	10	9.1	0.26 (0.14-0.47)	-[c]
**Educational attainment before 20 weeks of pregnancy**															
Attempted but did not achieve 5 A*-C GCSEs	10480 (62.0)	2,520	24	1 (ref)	1 (ref)	10035 (61.7)	2,360	23.5	. (.- .)	1 (ref)	11200 (60.8)	3,550	31.7	. (.- .)	1 (ref)
5 A*-C GCSEs	2575 (15.2)	545	21.2	0.82 (0.74-0.92)	1.12 (0.99-1.26)	2935 (18.0)	565	19.3	0.76 (0.68-0.85)	1.02 (0.90-1.15)	3200 (17.4)	810	25.3	0.70 (0.64-0.77)	0.99 (0.89-1.09)
Not linked to NPD	2720 (16.1)	390	14.3	0.46 (0.41-0.52)	-[c]	2360 (14.5)	340	14.4	0.45 (0.39-0.51)	-[c]	2870 (15.6)	540	18.8	0.47 (0.42-0.52)	-[c]
Had not attempted GCSEs prior to 20 weeks pregnancy	1135 (6.7)	610	53.7	4.05 (3.55-4.61)	1.51 (1.28-1.79)	950 (5.8)	470	49.5	3.94 (3.40-4.56)	1.63 (1.35-1.96)	1145 (6.2)	690	60.3	3.71 (3.25-4.22)	1.74 (1.48-2.06)
**Ever excluded, in pupil referral unit, or alternative provision**															
No	9680 (57.2)	2,245	23.2	1 (ref)	1 (ref)	9465 (58.2)	2,090	22.1	1 (ref)	1 (ref)	10645 (57.8)	3,180	29.9	1 (ref)	1 (ref)
Yes	4510 (26.7)	1,430	31.7	1.55 (1.43-1.69)	1.07 (0.98-1.17)	4455 (27.4)	1,310	29.4	1.50 (1.38-1.63)	1.04 (0.95-1.15)	4900 (26.6)	1,865	38.1	1.49 (1.39-1.61)	1.02 (0.94-1.11)
Not linked to NPD	2720 (16.1)	390	14.3	0.49 (0.43-0.55)	-[c]	2360 (14.5)	340	14.4	0.49 (0.43-0.56)	-[c]	2870 (15.6)	540	18.8	0.52 (0.46-0.58)	-[c]
**Ever persistently absent in a term (>=10% possible sessions)**															
No	8765 (51.8)	1,575	18	1 (ref)	1 (ref)	8810 (54.1)	1,615	18.3	1 (ref)	1 (ref)	9885 (53.7)	2,475	25	1 (ref)	1 (ref)
Yes	5425 (32.1)	2,105	38.8	3.13 (2.89-3.40)	1.62 (1.47-1.80)	5110 (31.4)	1,785	34.9	2.67 (2.45-2.90)	1.36 (1.23-1.52)	5660 (30.7)	2,575	45.5	2.79 (2.60-3.01)	1.54 (1.40-1.68)
Not linked to NPD	2720 (16.1)	390	14.3	0.68 (0.60-0.77)	-[c]	2360 (14.5)	340	14.4	0.63 (0.55-0.72)	-[c]	2870 (15.6)	540	18.8	0.67 (0.60-0.75)	-[c]

Table S7. Predictors of enrolment in the Family Nurse Partnership by financial year, among mothers aged 13-19 at last menstrual period, living in a Local Authority with an active FNP site at the time of first antenatal appointment – England, births between April 2010-March 2017 (continued)

	2016-17				
	**N eligible mothers**	**N enrolled in FNP**	**% enrolled in FNP**	**Crude odds ratio (95% CI)**	**Adjusted[a] odds ratio (95% CI)**
**Total**	15,470	4,605	29.8	-	-
**Maternal age at birth**					
13-15	260 (1.7)	165	63.5	5.31 (4.06-6.95)	2.77 (2.02-3.81)
16-17	2865 (18.5)	1,425	49.7	2.92 (2.66-3.21)	2.06 (1.84-2.31)
18-19	8750 (56.6)	2,375	27.1	1 (ref)	1 (ref)
20[b]	3590 (23.2)	635	17.7	0.53 (0.48-0.58)	0.61 (0.55-0.68)
**Ethnicity**					
White	12695 (82.1)	3,840	30.2	1 (ref)	1 (ref)
South Asian	480 (3.1)	105	21.9	0.61 (0.48-0.77)	0.84 (0.65-1.08)
Black	500 (3.2)	200	40	1.38 (1.12-1.70)	1.58 (1.27-1.98)
Mixed/other	870 (5.6)	255	29.3	0.93 (0.79-1.10)	1.04 (0.87-1.25)
Unknown	920 (5.9)	200	21.7	0.57 (0.48-0.67)	0.74 (0.61-0.88)
**Index of Multiple Deprivation (quintile)**					
Least deprived	895 (5.8)	265	29.6	1.00 (0.83-1.20)	1.00 (0.82-1.21)
2	1315 (8.5)	365	27.8	0.98 (0.83-1.14)	0.97 (0.82-1.15)
3	2210 (14.3)	630	28.5	1 (ref)	1 (ref)
4	3825 (24.7)	1,145	29.9	1.10 (0.98-1.25)	1.06 (0.94-1.21)
Most deprived	7165 (46.3)	2,145	29.9	1.17 (1.04-1.32)	1.07 (0.94-1.21)
Unknown	60 (0.4)	60	100	-	-
**Admission with diagnoses within 2 years before 20 weeks gestation**					
Mental health (excluding substance misuse and self-harm)	515 (3.3)	250	48.5	2.50 (2.08-3.00)	1.37 (1.08-1.74)
Adversity-related	735 (4.8)	380	51.7	2.78 (2.38-3.25)	1.27 (1.01-1.58)
Any chronic condition	1615 (10.4)	705	43.7	2.05 (1.83-2.29)	1.33 (1.13-1.57)
A&E visits	10130 (65.5)	3,325	32.8	1.61 (1.49-1.75)	1.36 (1.24-1.48)
**Gestational age at antenatal booking appointment**					
Before 10 weeks	4490 (29.0)	1,295	28.8	1 (ref)	1 (ref)
10-20 weeks	5835 (37.7)	1,655	28.4	0.92 (0.83-1.00)	0.88 (0.80-0.97)
20 weeks or more	760 (4.9)	235	30.9	0.96 (0.80-1.14)	0.85 (0.70-1.03)
Unknown	4385 (28.3)	1,420	32.4	1.15 (1.03-1.29)	1.00 (0.89-1.13)
**Linked to NPD**					
Linked to NPD	12820 (82.9)	4,095	31.9	1 (ref)	1 (ref)
Not linked to NPD	2570 (16.6)	490	19.1	0.46 (0.41-0.51)	0.87 (0.75-1.02)
Linked to NPD but not to NPD census	85 (0.5)	20	23.5	0.52 (0.30-0.90)	0.92 (0.51-1.64)
**Ever had a child protection plan or was looked after before 20 weeks pregnancy**					
No CPP or looked after	11425 (73.9)	3,370	29.5	1 (ref)	1 (ref)
Looked after (CPP)	950 (6.1)	475	50	2.52 (2.19-2.90)	1.76 (1.51-2.06)
Child protection plan, but not looked after	530 (3.4)	270	50.9	2.65 (2.20-3.18)	1.45 (1.19-1.77)

	2016-17				
	**N eligible mothers**	**N enrolled in FNP**	**% enrolled in FNP**	**Crude odds ratio (95% CI)**	**Adjusted[a] odds ratio (95% CI)**
Not linked to NPD	2570 (16.6)	490	19.1	0.52 (0.46-0.58)	-[c]
**Ever recorded as having Special Educational Needs before 20 weeks pregnancy**					
No	5395 (34.9)	1,435	26.6	1 (ref)	1 (ref)
Yes	7425 (48.0)	2,665	35.9	1.61 (1.49-1.75)	1.29 (1.18-1.42)
Not linked to NPD	2570 (16.6)	490	19.1	0.61 (0.54-0.69)	-[c]
Linked to NPD but not to NPD census	85 (0.5)	20	23.5	0.70 (0.40-1.22)	-[c]
**Ever recorded as receiving free school meals before 20 weeks of pregnancy**					
No	4500 (29.1)	1,200	26.7	1 (ref)	1 (ref)
Yes	8320 (53.8)	2,895	34.8	1.50 (1.38-1.63)	1.10 (1.00-1.21)
Not linked to NPD	2570 (16.6)	490	19.1	0.60 (0.53-0.68)	-[c]
Linked to NPD but not to NPD census	85 (0.5)	20	23.5	0.69 (0.39-1.21)	-[c]
**Ever in IDACI bottom decile before 20 weeks of pregnancy**					
No	7900 (51.1)	2,370	30	1 (ref)	1 (ref)
Yes	4920 (31.8)	1,730	35.2	1.34 (1.23-1.46)	1.03 (0.93-1.14)
Not linked to NPD	2570 (16.6)	490	19.1	0.52 (0.46-0.58)	-[c]
Linked to NPD but not to NPD census	85 (0.5)	20	23.5	0.60 (0.35-1.06)	-[c]
**Educational attainment before 20 weeks of pregnancy**					
Attempted but did not achieve 5 A*-C GCSEs	9455 (61.1)	3,025	32	. (.- .)	1 (ref)
5 A*-C GCSEs	2680 (17.3)	610	22.8	0.60 (0.54-0.66)	0.91 (0.81-1.02)
Not linked to NPD	2570 (16.6)	490	19.1	0.46 (0.41-0.51)	-[c]
Had not attempted GCSEs prior to 20 weeks pregnancy	765 (4.9)	480	62.7	4.11 (3.50-4.83)	1.72 (1.41-2.09)
**Ever excluded, in pupil referral unit, or alternative provision**					
No	8705 (56.3)	2,485	28.5	1 (ref)	1 (ref)
Yes	4195 (27.1)	1,630	38.9	1.62 (1.49-1.75)	1.06 (0.97-1.17)
Not linked to NPD	2570 (16.6)	490	19.1	0.54 (0.48-0.61)	-[c]
**Ever persistently absent in a term (>=10% possible sessions)**					
No	8480 (54.8)	2,095	24.7	1 (ref)	1 (ref)
Yes	4420 (28.6)	2,020	45.7	2.86 (2.63-3.10)	1.44 (1.30-1.60)
Not linked to NPD	2570 (16.6)	490	19.1	0.67 (0.60-0.76)	-[c]

[a]Adjusted models included all variables in the table as covariates

[b]Includes only mothers aged 19 at last menstrual period

[c]Estimates omitted due to multicollinearity

Note: numbers have been rounded to the nearest 5 in accordance with NHS Digital’s statistical disclosure rules for sub-national analyses. NPD – National Pupil Database
