# Supplementary material for: Characteristics of enrolment in an intensive home-visiting programme among eligible first-time adolescent mothers in England: a linked administrative data cohort study
Source: J Epidemiol Community Health. 2022 Oct 5;76(12):991–8. doi: 10.1136/jech-2021-217986 (PMC9664100; doi:10.1136/jech-2021-217986)
Supplement: Supplementary data [file jech-2021-217986supp002.pdf]

Supplementary Materials 2: secondary analysis – mothers aged 20-24 at last menstrual period

Methods

We used the FNP data to identify Local Authorities (LAs) that had extended their eligibility criteria to allow for recruitment of older mothers as those where at least 10 mothers aged 20-24 at last menstrual period and giving birth up to 31 March 2019 were enrolled in the FNP. We restricted the 20-24 year old cohort to mothers aged 20-24 at last menstrual period living in one of these LAs. We included mothers whose first antenatal appointment (or estimated date of 28 weeks gestation, if date missing) occurred from the month of enrolment of the first mother aged 20-24 in the local site (Figure). All FNP mothers aged 20-24 were linked to at least one HES inpatient hospital record.

Figure. Flow diagram of cohort selection

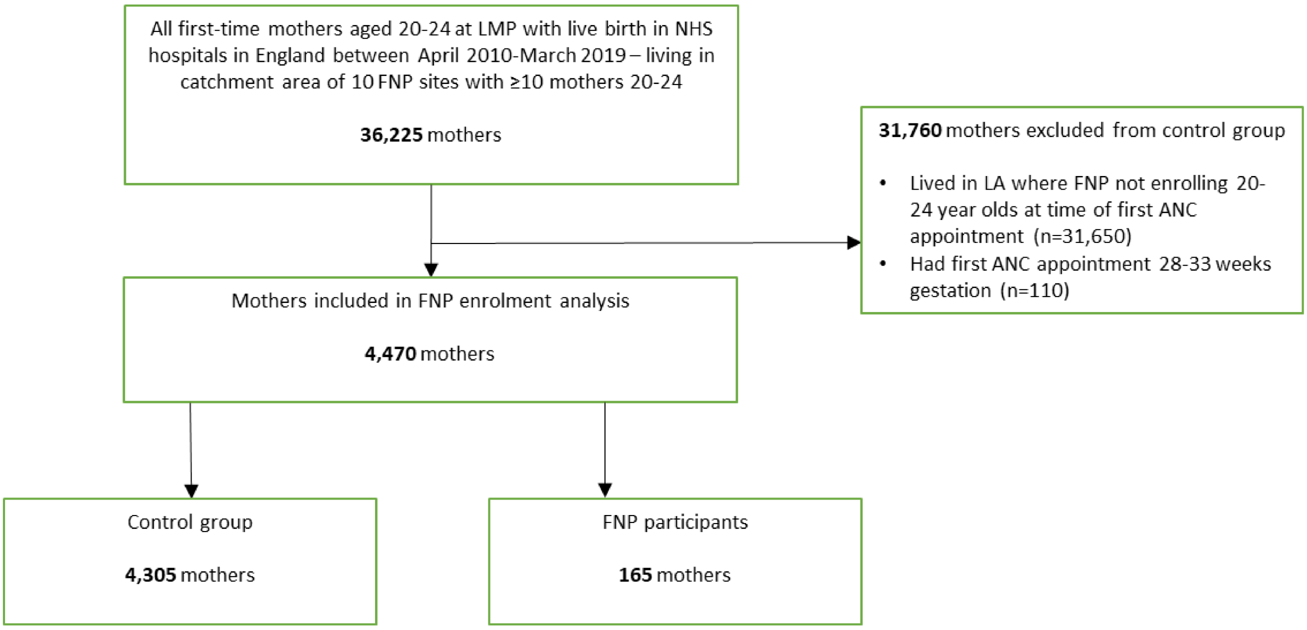

Note: numbers have been rounded to the nearest 5 in accordance with NHS Digital's statistical disclosure rules for sub-national analyses; totals may not be equal to the sum of component categories. ANC – antenatal care; FNP – Family Nurse Partnership; HES – Hospital Episode Statistics; LA – Local authority; LMP – last menstrual period.

We calculated the percentage enrolment as the percentage of FNP participants among the eligible study cohort, by site and across all sites. Multi-level logistic regression models with mothers nested within FNP sites were used to calculate crude and adjusted odds ratios (OR) of enrolment (adjusting for all predictors). The two least deprived quintiles were grouped to account for smaller numbers. Sample size of FNP participants was too small for analyses stratified by time, region and high-/low-enrolment.

Results - Enrolment and predictors of enrolment among mothers aged 20-24

Of the 4,470 mothers aged 20-24 at last menstrual period living in a LA where 20-24 year old mothers were recruited at the time of their first antenatal appointment, 165 were enrolled in the FNP, accounting for 3.7% [95% CI: 3.1%-4.2%] of eligible mothers (Table B.1). This percentage varied between 2.2% in Cornwall and 10.9% in Haringey.

Similar to the 13-19 age group, older mothers were less likely to be enrolled than younger ones. Women with recent A&E visits were also more likely to be enrolled than those without recent A&E visits (OR=2.4 [1.6-3.7]). Mothers who were looked after had 6.6 [3.6-12.2] times higher odds of being enrolled in the FNP,

and mothers ever recorded as having Special Educational Needs and receiving free school meals had 2-3 times higher odds of being enrolled in the FNP.

Table. Predictors of FNP enrolment among mothers aged 20-24 at last menstrual period, living in a Local Authority with an active FNP site at the time of first antenatal appointment, giving birth between November 2016 and March 2019

	N eligible mothers	N enrolled in FNP	% enrolled in FNP	Crude odds ratio (95% CI)	Adjusted[a] odds ratio (95% CI)
**Total**	**4,470**	**165**	**3.7**	**-**	**-**
**Maternal age at birth**					
20-21	1095 (24.5)	80	7.31	1 (ref)	1 (ref)
22-25[b]	3375 (75.6)	85	2.52	0.32 (0.23-0.43)	0.41 (0.29-0.58)
**Ethnicity**					
White	2460 (55.1)	85	3.46	1 (ref)	1 (ref)
South Asian	510 (11.4)	15	2.94	0.87 (0.47-1.63)	1.20 (0.62-2.33)
Black	395 (8.8)	25	6.33	2.07 (1.24-3.45)	1.71 (0.97-3.01)
Mixed/other	405 (9.1)	20	4.94	1.54 (0.91-2.62)	1.57 (0.90-2.77)
Unknown	695 (15.6)	15	2.16	0.71 (0.41-1.22)	1.15 (0.64-2.07)
**Index of Multiple Deprivation (quintile)**					
Least deprived or 2[b]	410 (9.2)	10	2.44	1.04 (0.49-2.17)	1.20 (0.55-2.63)
3	835 (18.7)	20	2.4	1 (ref)	1 (ref)
4	1345 (30.1)	40	2.97	1.29 (0.75-2.23)	1.06 (0.60-1.87)
Most deprived	1875 (42.0)	85	4.53	2.08 (1.24-3.47)	1.45 (0.83-2.52)
**Admission with diagnoses within 2 years before 20 weeks gestation**					
Mental health (excluding substance misuse and self-harm)	85 (1.9)	10	11.76	4.51 (2.37-8.58)	0.76 (0.31-1.89)
Adversity-related	60 (1.3)	10	16.67	6.64 (3.43-12.88)	2.20 (0.90-5.35)
Any chronic condition	315 (7.1)	30	9.52	3.44 (2.25-5.26)	1.86 (1.02-3.39)
A&E visit	2465 (55.2)	130	5.27	3.57 (2.40-5.30)	2.41 (1.57-3.68)
**Gestational age at antenatal booking appointment**					
Before 10 weeks	815 (18.3)	30	3.7	1 (ref)	1 (ref)
10-20 weeks	1240 (27.8)	50	4	1.03 (0.64-1.65)	0.98 (0.60-1.61)
20 weeks or more	165 (3.7)	10	6.1	1.53 (0.72-3.23)	1.90 (0.84-4.32)
Unknown	2245 (50.3)	70	3.1	0.82 (0.51-1.32)	0.77 (0.46-1.28)
**Linked to NPD**					
Linked to NPD	2610 (58.5)	110	4.2	1 (ref)	1 (ref)
Not linked to NPD	1810 (40.5)	50	2.8	0.54 (0.38-0.77)	2.41 (1.22-4.75)
Linked to NPD but not to NPD census	50 (1.1)	-	-	0.72 (0.17-3.08)	2.79 (0.58-13.35)
**Ever had a child protection plan or was looked after**					
No CPP or looked after before 20wks pregnancy	2560 (57.3)	90	3.5	1 (ref)	1 (ref)
Looked after before 20wks pregnancy (CPP)	85 (1.9)	25	29.4	10.53 (6.12-18.10)	6.60 (3.58-12.18)
Child protection plan before 20wks pregnancy, but not looked after	15 (0.3)	-	-	4.52 (0.99-20.68)	1.78 (0.35-9.01)
Not linked to NPD	1810 (40.5)	50	2.8	0.71 (0.49-1.03)	-[d]
**Ever recorded as having Special Educational Needs**					
No	1430 (32.0)	30	2.1	1 (ref)	1 (ref)
Yes	1180 (26.4)	85	7.2	3.81 (2.46-5.90)	2.04 (1.24-3.35)
Not linked to NPD	1810 (40.5)	50	2.8	1.24 (0.76-2.03)	-[d]
Linked to NPD but not to NPD census	50 (1.1)	-	-	1.66 (0.37-7.39)	-[d]
**Ever recorded as receiving free school meals**					
No	1370 (30.7)	25	1.8	1 (ref)	1 (ref)
Yes	1240 (27.8)	90	7.3	4.37 (2.74-6.96)	2.86 (1.72-4.76)

	N eligible mothers	N enrolled in FNP	% enrolled in FNP	Crude odds ratio (95% CI)	Adjusted[a] odds ratio (95% CI)
Not linked to NPD	1810 (40.5)	50	2.8	1.44 (0.86-2.40)	-[d]
Linked to NPD but not to NPD census	50 (1.1)	-	-	1.92 (0.43-8.59)	-[d]
**Ever in IDACI bottom decile**					
No	1710 (38.3)	60	3.5	1 (ref)	1 (ref)
Yes	900 (20.2)	50	5.6	1.64 (1.09-2.45)	0.77 (0.48-1.22)
Not linked to NPD	1810 (40.5)	50	2.8	0.68 (0.45-1.02)	-[d]
Linked to NPD but not to NPD census	50 (1.1)	-	-	0.91 (0.21-3.95)	-[d]
**Educational attainment**					
Attempted but did not acheive 5 A*-C GCSEs prior to 20 weeks	1740 (39.0)	90	5.2	1 (ref)	1 (ref)
5 A*-C GCSEs gained prior to 20 weeks	915 (20.5)	20	2.2	0.43 (0.27-0.69)	0.97 (0.56-1.67)
Unlinked	1810 (40.5)	50	2.8	0.43 (0.30-0.63)	-[d]
**Ever excluded, in pupil referral unit, or alternative provision**					
No	2155 (48.3)	75	3.5	1 (ref)	1 (ref)
Yes	505 (11.3)	40	7.9	2.14 (1.43-3.22)	1.03 (0.65-1.65)
Not linked to NPD	1810 (40.5)	50	2.8	0.67 (0.46-0.99)	-[d]

[a]Adjusted models included all variables in the table as covariates

[b]Includes only mothers aged 24 at last menstrual period

[c]The two least deprived quintiles were grouped due to small numbers

[d]Estimates omitted due to multicollinearity

Note: numbers have been rounded to the nearest 5 in accordance with NHS Digital's statistical disclosure rules for sub-national analyses. NPD – National Pupil Database
